# Supplementary material for: Bayesian Lesion Estimation with a Structured Spike-and-Slab Prior
Source: J Am Stat Assoc. 2023 Nov 16;119(545):66–80. doi: 10.1080/01621459.2023.2278201 (PMC11315456; doi:10.1080/01621459.2023.2278201)
Supplement: Supplemental Material [file UASA_A_2278201_SM3041.pdf]

Bayesian Lesion Estimation with a Structured Spike-and-Slab Prior

SUPPLEMENTARY MATERIAL

1 BLESS Model Hierarchy

$$
\text{(1) Probit Model}
\begin{cases}
[y_i(s_j)|p_i(s_j)] \sim \text{Bernoulli}(p_i(s_j)) \\
\Phi^{-1}\left\{\mathbb{E}[y_i(s_j)|p_i(s_j)]\right\} = \eta_i(s_j) = \boldsymbol{x}_i^{\mathrm{T}}\boldsymbol{\beta}(s_j) + \beta_0(s_j) \\
\Phi^{-1}\left\{\Pr[y_i(s_j) = 1|\eta_i(s_j)]\right\} = \boldsymbol{x}_i^{\mathrm{T}}\boldsymbol{\beta}(s_j) + \beta_0(s_j)
\end{cases}
$$

$$
\text{(2) Latent Model}
\begin{cases}
\Pr[y_i(s_j)|z_i(s_j)] = \begin{cases} 1, & z_i(s_j) > 0, \\ 0, & z_i(s_j) \leq 0, \end{cases} \\
z_i(s_j) \sim \mathcal{N}(\boldsymbol{x}_i^{\mathrm{T}}\boldsymbol{\beta}(s_j) + \beta_0(s_j),\ 1)
\end{cases}
$$

$$
\text{(3) Spike-and-Slab Prior}
\begin{cases}
\beta_p(s_j) \sim \mathcal{N}(0,\ \nu_0(1-\gamma_p(s_j)) + \nu_1\gamma_p(s_j)) \\
\gamma_p(s_j) \sim \text{Bernoulli}(\sigma(\theta_p(s_j)))
\end{cases}
$$

$$
\text{(4) MCAR Prior}
\begin{cases}
[\boldsymbol{\theta}(s_j) \mid \boldsymbol{\theta}(-s_j), \boldsymbol{\Sigma}] \sim \mathcal{N}\left(\frac{\sum_{s_r \in \partial s_j} \boldsymbol{\theta}(s_r)}{n(s_j)}, \frac{\boldsymbol{\Sigma}}{n(s_j)}\right) \\
\boldsymbol{\Sigma}^{-1} \sim \text{Wishart}(\nu,\ \boldsymbol{I})
\end{cases}
$$

Figure 1: BLESS Model Hierarchy. (1) Bayesian Generalised Linear Model (GLM) with probit link function which relates the lesion probability $p_i(s_j)$ for every subject i at every voxel s_j to the linear predictor $\eta_i(s_j) = \boldsymbol{x}_i^{\mathrm{T}}\boldsymbol{\beta}(s_j)$, where $\boldsymbol{x}_i$ contains the subject specific data and $\boldsymbol{\beta}(s_j)$ is a spatially-varying parameter. (2) The latent model describes the commonly used data augmentation approach for Bayesian probit regression which assumes that the binary outcomes $y_i(s_j)$ have an underlying normal regression structure on latent continuous variables $\boldsymbol{z}_i(s_j)$. (3) Spatially-varying, continuous version of the spike-and-slab prior on the parameters $\boldsymbol{\beta}(s_j)$ in the form of a mixture of normal distributions where $0 < \nu_0 < \nu_1$ (4) Parameter $\boldsymbol{\theta}(s_j)$ introduces the spatial structure within the probability of inclusion/exclusion $\sigma(\boldsymbol{\theta}(s_j))$, where $\sigma(\cdot)$ is the logistic function, with a multivariate conditional autoregressive (MCAR) prior.

2 Derivation of Variational Inference Algorithm

2.1 Joint Distribution

$$
\begin{aligned}
Q &= p(\boldsymbol{y}, \boldsymbol{z}, \boldsymbol{\beta}, \boldsymbol{\beta}_0, \boldsymbol{\gamma}, \boldsymbol{\theta}, \boldsymbol{\Sigma}) \\
&= p(\boldsymbol{y}|\boldsymbol{z})p(\boldsymbol{z}|\boldsymbol{\beta}, \boldsymbol{\beta}_0)p(\boldsymbol{\beta}_0)p(\boldsymbol{\beta}|\boldsymbol{\gamma})p(\boldsymbol{\gamma}|\boldsymbol{\theta})p(\boldsymbol{\theta}|\boldsymbol{\Sigma})p(\boldsymbol{\Sigma}^{-1}) \\
&= \prod_{i=1}^{N}\prod_{j=1}^{M} \Pr(y_i(s_j)|z_i(s_j)) \\
&\times \prod_{i=1}^{N}\prod_{j=1}^{M} \mathcal{N}(z_i(s_j); \boldsymbol{x}_i^{\mathrm{T}}\boldsymbol{\beta}(s_j) + \beta_0(s_j), 1) \\
&\times \prod_{j=1}^{M} \mathcal{N}(\beta_0(s_j); \mu_{\beta_0}, \sigma_{\beta_0}^2) \\
&\times \prod_{j=1}^{M} \mathcal{N}(\boldsymbol{\beta}(s_j); \boldsymbol{0}, \mathrm{diag}\{\nu_0(1-\gamma_p(s_j)) + \nu_1\gamma_p(s_j)\}_{p=1}^{P}) \\
&\times \prod_{p=1}^{P}\prod_{j=1}^{M} Bernoulli(\gamma_p(s_j); \sigma(\theta_p(s_j))) \\
&\times \mathcal{N}(\boldsymbol{\theta}; \boldsymbol{0}, \boldsymbol{\Sigma}(\boldsymbol{D}-\boldsymbol{W})^{-1}) \\
&\times Wishart(\boldsymbol{\Sigma}^{-1}; P, \boldsymbol{I})
\end{aligned}
$$

$$
\begin{aligned}
Q^* \propto\ & \mathbb{E}_{q(z,\beta,\beta_0,\gamma,\theta,\Sigma^{-1})}\left[-\frac{1}{2}\sum_{i=1}^{N}\sum_{j=1}^{M}(z_i(s_j)-\boldsymbol{x}_i^{\mathrm{T}}\boldsymbol{\beta}(s_j)-\beta_0(s_j))^2-\frac{1}{2\sigma_{\beta_0}^2}\sum_{j=1}^{M}(\beta_0(s_j)-\mu_{\beta_0})^2\right.\\
&-\frac{1}{2}\sum_{j=1}^{M}\log(|\mathrm{diag}\{\nu_0(1-\gamma_p(s_j))+\nu_1\gamma_p(s_j)\}_{p=1}^{P}|)\\
&-\frac{1}{2}\sum_{j=1}^{M}\boldsymbol{\beta}(s_j)^{\mathrm{T}}\mathrm{diag}\{\nu_0(1-\gamma_p(s_j))+\nu_1\gamma_p(s_j)\}_{p=1}^{P}\boldsymbol{\beta}(s_j)\\
&+\sum_{p=1}^{P}\sum_{j=1}^{M}\gamma_p(s_j)\log(\sigma(\theta_p(s_j))+\sum_{p=1}^{P}\sum_{j=1}^{M}(1-\gamma_p(s_j)\log(1-\sigma(\theta_p(s_j)))\\
&+\frac{1}{2}\sum_{j=1}^{M}\log\left\{|\boldsymbol{\Sigma}^{-1}|\right\}-\frac{1}{2}\sum_{s_i\sim s_j}[\boldsymbol{\theta}(s_i)-\boldsymbol{\theta}(s_j)]^{\mathrm{T}}\boldsymbol{\Sigma}^{-1}[\boldsymbol{\theta}(s_i)-\boldsymbol{\theta}(s_j)]\\
&\left.+\frac{1}{2}\left\{(\nu-P-1)\log\{|\boldsymbol{\Sigma}^{-1}|\}-\mathrm{tr}(\boldsymbol{\Sigma}^{-1})\right\}\right].\\
\geq\ & \mathbb{E}_{q(z,\beta,\beta_0,\gamma,\theta,\Sigma^{-1})}\left[-\frac{1}{2}\sum_{i=1}^{N}\sum_{j=1}^{M}(z_i(s_j)-\boldsymbol{x}_i^{\mathrm{T}}\boldsymbol{\beta}(s_j)-\beta_0(s_j))^2-\frac{1}{2\sigma_{\beta_0}^2}\sum_{j=1}^{M}(\beta_0(s_j)-\mu_{\beta_0})^2\right.\\
&-\frac{1}{2}\sum_{j=1}^{M}\log(|\mathrm{diag}\{\nu_0(1-\gamma_p(s_j))+\nu_1\gamma_p(s_j)\}_{p=1}^{P}|)\\
&-\frac{1}{2}\sum_{j=1}^{M}\boldsymbol{\beta}(s_j)^{\mathrm{T}}\mathrm{diag}\{\nu_0(1-\gamma_p(s_j))+\nu_1\gamma_p(s_j)\}_{p=1}^{P}\boldsymbol{\beta}(s_j)\\
&+\sum_{p=1}^{P}\sum_{j=1}^{J}\left[\log(\sigma(\xi_p(s_j)))+\theta_p(s_j)\gamma_p(s_j)-\frac{(\theta_p(s_j)+\xi_p(s_j))}{2}-\lambda(\xi_p(s_j))(\theta_p(s_j)^2-\xi_p(s_j)^2)\right]\\
&+\frac{1}{2}\sum_{j=1}^{M}\log\left\{|\boldsymbol{\Sigma}^{-1}|\right\}-\frac{1}{2}\sum_{s_i\sim s_j}[\boldsymbol{\theta}(s_i)-\boldsymbol{\theta}(s_j)]^{\mathrm{T}}\boldsymbol{\Sigma}^{-1}[\boldsymbol{\theta}(s_i)-\boldsymbol{\theta}(s_j)]\\
&\left.+\frac{1}{2}\left\{(\nu-P-1)\log\{|\boldsymbol{\Sigma}^{-1}|\}-\mathrm{tr}(\boldsymbol{\Sigma}^{-1})\right\}\right].
\end{aligned}
$$

2.2 Variational Approximations

2.2.1 Update $z_i(s_j)$

Assume $y_i(s_j) = 1$ and $\boldsymbol{\eta}(s_j) = \boldsymbol{x}_i^T \mathbb{E}_{q(\beta)}[\boldsymbol{\beta}(s_j)] + \mathbb{E}_{q(\beta_0)}[\beta_0(s_j)]$:

$$\begin{aligned}
\ln(q^*(z_i(s_j))) &\propto \mathbb{E}_{q(\beta,\beta_0,\gamma,\theta,\Sigma)}[\ln\{p(y_i(s_j), z_i(s_j), \boldsymbol{\beta}(s_j), \beta_0(s_j), \boldsymbol{\gamma}(s_j), \boldsymbol{\theta}(s_j), \boldsymbol{\Sigma}|\boldsymbol{x}_i)\}] \\
&\propto \ln\{p(y_i(s_j)|z_i(s_j))\} + \mathbb{E}_{q(\beta,\beta_0)}\left[\ln\{p(z_i(s_j)|\boldsymbol{x}_i, \boldsymbol{\beta}(s_j), \beta_0(s_j))\}\right] \\
&\propto y_i(s_j)\ln\{\mathbb{1}(z_i(s_j) > 0)\} + (1 - y_i(s_j))\ln\{\mathbb{1}(z_i(s_j) \leq 0)\} \\
&- \frac{1}{2}\mathbb{E}_{q(\beta,\beta_0)}[(z_i(s_j) - \boldsymbol{x}_i^{\mathrm{T}}\boldsymbol{\beta}(s_j) - \beta_0(s_j))^2] \\
&\propto \ln\{\mathbb{1}(z_i(s_j) > 0)\} - \frac{1}{2}z_i(s_j)^2 + z_i(s_j)[\mathbb{E}_{q(\beta)}[\boldsymbol{\beta}(s_j)]^{\mathrm{T}}\boldsymbol{x}_i + \mathbb{E}_{q(\beta_0)}[\beta_0(s_j)]] \\
&\propto \ln\{\mathbb{1}(z_i(s_j) > 0)\} - \frac{1}{2}z_i(s_j)^2 + z_i(s_j)\eta_i(s_j)
\end{aligned}$$

$$q^*(z_i(s_j)) = \begin{cases} \mathcal{TN}_+(z_i(s_j); \eta_i(s_j), 1), & \text{if } y_i(s_j) = 1, \\ \mathcal{TN}_-(z_i(s_j); \eta_i(s_j), 1), & \text{if } y_i(s_j) = 0. \end{cases}$$

2.2.2 Update $\beta(s_j)$

$$\begin{aligned}
\ln(q^*(\boldsymbol{\beta}(s_j))) &\propto \mathbb{E}_{q(z,\beta_0,\gamma,\theta,\Sigma)}[\ln\{p(y_i(s_j), z_i(s_j), \boldsymbol{\beta}(s_j), \beta_0(s_j), \boldsymbol{\gamma}(s_j), \boldsymbol{\theta}(s_j), \boldsymbol{\Sigma}|\boldsymbol{x}_i)\}] \\
&\propto \mathbb{E}_{q(\gamma)}[\ln\{p(\boldsymbol{\beta}(s_j)|\boldsymbol{\gamma}(s_j))\}] + \mathbb{E}_{q(z,\beta_0)}[\ln\{p(\boldsymbol{z}(s_j)|X, \boldsymbol{\beta}(s_j), \beta_0(s_j))\}] \\
&\propto -\frac{1}{2}\boldsymbol{\beta}(s_j)^{\mathrm{T}}\mathbb{E}_{q(\gamma)}[\mathrm{diag}\{\nu_0(1-\gamma_p(s_j)) + \nu_1\gamma_p(s_j)\}_{p=1}^{P}]^{-1}\boldsymbol{\beta}(s_j) \\
&- \frac{1}{2}\mathbb{E}_{q(z,\beta_0)}\left[[\boldsymbol{z}(s_j) - X\boldsymbol{\beta}(s_j) - \beta_0(s_j)]^{\mathrm{T}}[\boldsymbol{z}(s_j) - X\boldsymbol{\beta}(s_j) - \beta_0(s_j)]\right] \\
&\propto \mathbb{E}_{q(z,\beta_0)}\left[\boldsymbol{\beta}(s_j)^T X^T[\boldsymbol{z}(s_j) - \beta_0(s_j)] - \frac{1}{2}\boldsymbol{\beta}(s_j)^T X^T X\boldsymbol{\beta}(s_j)\right] \\
&- \frac{1}{2}\mathrm{tr}(\mathbb{E}_{q(\gamma)}[\mathrm{diag}\{\nu_0(1-\gamma_p(s_j)) + \nu_1\gamma_p(s_j)\}_{p=1}^{P}]^{-1}\boldsymbol{\beta}(s_j)\boldsymbol{\beta}(s_j)^T) \\
&\propto \boldsymbol{\beta}(s_j)^T X^T[\mathbb{E}_{q(z}[\boldsymbol{z}(s_j)] - \mathbb{E}_{q(\beta_0)}[\beta_0(s_j)] \\
&- \frac{1}{2}\mathrm{tr}([X^T X + \mathbb{E}_{q(\gamma)}[\mathrm{diag}\{\nu_0(1-\gamma_p(s_j)) + \nu_1\gamma_p(s_j)\}_{p=1}^{P}]^{-1}]\boldsymbol{\beta}(s_j)\boldsymbol{\beta}(s_j)^T)
\end{aligned}$$

$$\begin{aligned}
q^*(\boldsymbol{\beta}(s_j)) &= \mathcal{N}(\boldsymbol{\beta}(s_j); \mu_{\beta(s_j)}, \Sigma_{\beta(s_j)}) \\
\mu_{\beta(s_j)} &= \Sigma_{\beta(s_j)}X^{\mathrm{T}}\left[\mathbb{E}_{q(z)}[\boldsymbol{z}(s_j)] - \mathbb{E}_{q(\beta_0)}[\beta_0(s_j)]\right] \\
\Sigma_{\beta(s_j)} &= \left[X^{\mathrm{T}}X + \mathbb{E}_{q(\gamma)}[\mathrm{diag}\{\nu_0(1-\gamma_p(s_j)) + \nu_1\gamma_p(s_j)\}_{p=1}^{P}]^{-1}\right]^{-1}
\end{aligned}$$

2.2.3 Update $\beta_0(s_j)$

$$
\begin{aligned}
\ln(q^*(\beta_0(s_j))) &\propto \mathbb{E}_{q(z,\beta,\gamma,\theta,\Sigma)}[\ln\{p(y_i(s_j), z_i(s_j), \boldsymbol{\beta}(s_j), \beta_0(s_j), \boldsymbol{\gamma}(s_j), \boldsymbol{\theta}(s_j), \boldsymbol{\Sigma}|\boldsymbol{x}_i)\}] \\
&\propto \mathbb{E}_{q(z,\beta)}[\ln p(\boldsymbol{z}(s_j)|X, \beta_0(s_j), \boldsymbol{\beta}(s_j))] + \ln p(\beta_0(s_j)) \\
&\propto \mathbb{E}_{q(z,\beta)}[\ln \mathcal{N}(\boldsymbol{z}(s_j); X\boldsymbol{\beta}(s_j) + \beta_0(s_j), I)] + \ln \mathcal{N}(\beta_0(s_j); 0, \sigma^2_{\beta_0}) \\
&\propto \sum_{i=1}^{N} \beta_0(s_j)[\mathbb{E}_{q(z)}[z_i(s_j)] - \boldsymbol{x}_i^T \mathbb{E}_{q(\beta)}[\boldsymbol{\beta}(s_j)]] - \frac{N}{2}\beta_0(s_j)^2 - \frac{1}{2\sigma^2_{\beta_0}}\beta_0(s_j)^2
\end{aligned}
$$

$$
\begin{aligned}
q^*(\beta_0(s_j)) &= \mathcal{N}(\beta_0(s_j); \mu_{\beta_0(s_j)}, \sigma^2_{\beta_0(s_j)}) \\
\mu_{\beta_0(s_j)} &= [N + \frac{1}{\sigma^2_{\beta_0}}]^{-1}[\sum_{i=1}^{N} \mathbb{E}_{q(z)}[z_i(s_j)] - \boldsymbol{x}_i^T \mathbb{E}_{q(\beta)}[\boldsymbol{\beta}(s_j)]] \\
\sigma^2_{\beta_0(s_j)} &= [N + \frac{1}{\sigma^2_{\beta_0}}]^{-1}
\end{aligned}
$$

2.2.4 Update $\gamma_p(\mathbf{s}_j)$

$$
\begin{aligned}
\ln(q^*(\gamma_p(s_j))) &\propto \mathbb{E}_{q(z,\beta,\beta_0,\theta,\Sigma)}[\ln\{p(y_i(s_j), z_i(s_j), \boldsymbol{\beta}(s_j), \beta_0(s_j), \boldsymbol{\gamma}(s_j), \boldsymbol{\theta}(s_j), \boldsymbol{\Sigma}|\boldsymbol{x}_i)\}] \\
&\propto \mathbb{E}_{q(\theta)}[\ln p(\gamma_p(s_j)|\theta_p(s_j))] + \mathbb{E}_{q(\beta)}[\ln p(\beta_p(s_j)|\gamma_p(s_j))] \\
&\propto \mathbb{E}_{q(\beta)}[\ln \mathcal{N}(\beta_p(s_j); 0, \nu_0(1-\gamma_p(s_j)) + \nu_1\gamma_p(s_j))] \\
&+ \mathbb{E}_{q(\theta)}[\ln Bernoulli(\gamma_p(s_j); \sigma(\theta_p(s_j)))] \\
&\geq \mathbb{E}_{q(\beta)}[-\frac{1}{2}\ln(\nu_0(1-\gamma_p(s_j)) + \nu_1\gamma_p(s_j)) - \frac{1}{2}\frac{\beta_p(s_j)^2}{\nu_0(1-\gamma_p(s_j)) + \nu_1\gamma_p(s_j)}] \\
&+ \mathbb{E}_{q(\theta)}[\gamma_p(s_j)[\ln\sigma(\xi_p(s_j)) + \frac{(\theta_p(s_j) - \xi_p(s_j))}{2} - \lambda(\xi_p(s_j))[\theta_p(s_j)^2 - \xi_p(s_j)^2]]] \\
&+ \mathbb{E}_{q(\theta)}[(1-\gamma_p(s_j))[\ln\sigma(\xi_p(s_j)) - \frac{(\theta_p(s_j) + \xi_p(s_j))}{2} \\
&- \lambda(\xi_p(s_j))[\theta_p(s_j)^2 - \xi_p(s_j)^2]]] \\
&= \mathbb{E}_{q(\beta)}[-\frac{1}{2}\ln(\nu_0(1-\gamma_p(s_j)) + \nu_1\gamma_p(s_j)) - \frac{1}{2}\frac{\beta_p(s_j)^2}{\nu_0(1-\gamma_p(s_j)) + \nu_1\gamma_p(s_j)}] \\
&+ \mathbb{E}_{q(\theta)}[\gamma_p(s_j)\theta_p(s_j) + \ln\sigma(\xi_p(s_j)) - \frac{(\theta_p(s_j) + \xi_p(s_j))}{2} \\
&- \lambda(\xi_p(s_j))[\theta_p(s_j)^2 - \xi_p(s_j)^2]] \\
&\propto \mathbb{E}_{q(\beta)}[-\frac{1}{2}\ln(\nu_0(1-\gamma_p(s_j)) + \nu_1\gamma_p(s_j)) - \frac{1}{2}\frac{\beta_p(s_j)^2}{\nu_0(1-\gamma_p(s_j)) + \nu_1\gamma_p(s_j)}] \\
&+ \mathbb{E}_{q(\theta)}[\gamma_p(s_j)\theta_p(s_j)]
\end{aligned}
$$

$$
\begin{aligned}
q^*(\gamma_p(s_j)) &= Bernoulli(\gamma_p(s_j); \mu_{\gamma_p(s_j)}) \\
\mu_{\gamma_p(s_j)} &= \frac{q(\gamma_p(s_j)=1)}{\sum_{k=\{0,1\}} q(\gamma_p(s_j)=k)} \\
q(\gamma_p(s_j)=1) &= \exp\left\{-\frac{1}{2}\ln\nu_1 - \frac{\mathbb{E}_{q(\beta)}[\beta_p(s_j)^2]}{2\nu_1} + \mathbb{E}_{q(\theta)}[\theta_p(s_j)]\right\} \\
q(\gamma_p(s_j)=0) &= \exp\left\{-\frac{1}{2}\ln\nu_0 - \frac{\mathbb{E}_{q(\beta)}[\beta_p(s_j)^2]}{2\nu_0}\right\}
\end{aligned}
$$

2.2.5 Update $\theta_p(\mathbf{s}_j)$

$$
\begin{aligned}
\ln(q(\boldsymbol{\theta})) &\propto \mathbb{E}_{q(z,\beta,\beta_0,\gamma,\Sigma)}[\ln\{p(y_i(s_j), z_i(s_j), \boldsymbol{\beta}(s_j), \beta_0(s_j), \boldsymbol{\gamma}(s_j), \boldsymbol{\theta}(s_j), \boldsymbol{\Sigma}|\boldsymbol{x}_i)\}] \\
&\propto \mathbb{E}_{q(\gamma)}[\ln p(\boldsymbol{\gamma}(s_j)|\boldsymbol{\theta}(s_j))] + \mathbb{E}_{q(\Sigma^{-1})}[\ln p(\boldsymbol{\theta}|\boldsymbol{\Sigma}^{-1})] \\
&\geq \mathbb{E}_{q(\gamma)}[\sum_{p=1}^{P}\{\theta_p(s_j)\gamma_p(s_j) + \ln\sigma(\xi_p(s_j)) - \frac{(\theta_p(s_j)+\xi_p(s_j))}{2} \\
&- \lambda(\xi_p(s_j))[\theta_p(s_j)^2 - \xi_p(s_j)^2]\}] \\
&- \mathbb{E}_{q(\Sigma^{-1})}[\frac{1}{2}\sum s_i \sim s_j[\boldsymbol{\theta}(s_i) - \boldsymbol{\theta}(s_j)]^T\Sigma^{-1}[\boldsymbol{\theta}(s_i) - \boldsymbol{\theta}(s_j)]] \\
&\propto \sum_{p=1}^{P}\left\{\theta_p(s_j)\mathbb{E}_{q(\gamma)}[\gamma_p(s_j)] - \frac{1}{2}\theta_p(s_j) - \lambda(\xi_p(s_j))\theta_p(s_j)^2\right\} \\
&- \frac{1}{2}tr(\mathbb{E}_{q(\Sigma^{-1})}[\Sigma^{-1}]\sum_{s_i\sim s_j}[\boldsymbol{\theta}(s_i) - \boldsymbol{\theta}(s_j)][\boldsymbol{\theta}(s_i) - \boldsymbol{\theta}(s_j)]^T) \\
&\propto \left[\mathbb{E}_{q(\gamma)}[\boldsymbol{\gamma}(s_j)] - \frac{1}{2} + \mathbb{E}_{q(\Sigma^{-1})}[\Sigma^{-1}]\sum_{s_i\sim s_j}\boldsymbol{\theta}(s_i)\right]^T \boldsymbol{\theta}(s_j) \\
&- \frac{1}{2}\boldsymbol{\theta}(s_j)^T\left[n(s_j)\mathbb{E}_{q(\Sigma^{-1})}[\Sigma^{-1}] + 2diag\{\lambda(\xi_p(s_j)\}_{p=1}^{P}\right]\boldsymbol{\theta}(s_j)
\end{aligned}
$$

$$
\begin{aligned}
q^*(\boldsymbol{\theta}(s_j)) &= \mathcal{N}(\boldsymbol{\theta}(s_j); \mu_{\theta(s_j)}, \Sigma_{\theta(s_j)}) \\
\mu_{\theta(s_j)} &= \Sigma_{\theta(s_j)}\left[\mathbb{E}_{q(\Sigma^{-1})}[\Sigma^{-1}]\sum_{s_i\sim s_j}\boldsymbol{\theta}(s_i) + \mathbb{E}_{q(\gamma)}[\boldsymbol{\gamma}(s_j)] - \frac{1}{2}\right] \\
\Sigma_{\theta(s_j)} &= \left[n(s_j)\mathbb{E}_{q(\Sigma^{-1})}[\Sigma^{-1}] + 2\text{diag}\{\lambda(\xi_p(s_j))\}_{p=1}^{P}\right]^{-1}
\end{aligned}
$$

2.2.6 Update Σ^{-1}

$$\begin{aligned}
\ln(q(\boldsymbol{\Sigma}^{-1})) &\propto \mathbb{E}_{q(z,\beta,\beta_0,\gamma,\theta)}[\ln\{p(y_i(s_j), z_i(s_j), \boldsymbol{\beta}(s_j), \beta_0(s_j), \boldsymbol{\gamma}(s_j), \boldsymbol{\theta}(s_j), \boldsymbol{\Sigma}|\boldsymbol{x}_i)\}] \\
&\propto \mathbb{E}_{q(\theta}[\ln p(\boldsymbol{\theta}|\boldsymbol{\Sigma}^{-1})] + \ln p(\boldsymbol{\Sigma}^{-1}) \\
&\propto \frac{M}{2}\ln|\boldsymbol{\Sigma}^{-1}| - \frac{1}{2}\mathrm{tr}(\mathbb{E}_{q(\theta)}[\sum_{s_i\sim s_j}[\boldsymbol{\theta}(s_i)-\boldsymbol{\theta}(s_j)][\boldsymbol{\theta}(s_i)-\boldsymbol{\theta}(s_j)]^{\mathrm{T}}\boldsymbol{\Sigma}^{-1}]) \\
&+ \frac{\nu-P-1}{2}\ln|\boldsymbol{\Sigma}^{-1}| - \frac{1}{2}\mathrm{tr}(\boldsymbol{\Sigma}^{-1})
\end{aligned}$$

$$\begin{aligned}
q^*(\boldsymbol{\Sigma}^{-1}) &= Wishart(\boldsymbol{\Sigma}^{-1}; \nu_\Sigma, I_\Sigma) \\
\nu_\Sigma &= M + \nu \\
I_\Sigma &= \left[\mathbb{E}_{q(\theta)}[\sum_{s_i\sim s_j}[\boldsymbol{\theta}(s_i)-\boldsymbol{\theta}(s_j)][\boldsymbol{\theta}(s_i)-\boldsymbol{\theta}(s_j)]^{\mathrm{T}}] + \boldsymbol{I}\right]^{-1}
\end{aligned}$$

2.2.7 Update $\xi_p(s_j)$

$$\begin{aligned}
p(\gamma_p(s_j)|\theta_p(s_j)) &= \sigma(\theta_p(s_j))^{\gamma_p(s_j)}(1-\sigma(\theta_p(s_j)))^{1-\gamma_p(s_j)} = \exp(\theta_p(s_j)\gamma_p(s_j))\sigma(-\theta_p(s_j)) \\
h(\theta_p(s_j), \gamma_p(s_j), \xi_p(s_j)) &= \sigma(\theta_p(s_j))\exp\left(\theta_p(s_j)\gamma_p(s_j) - \frac{\xi_p(s_j)}{2} - \lambda(\xi_p(s_j))(\theta_p(s_j)^2 - \xi_p(s_j)^2)\right) \\
Q &\propto \sum_{p=1}^{P}\sum_{j=1}^{M}\mathbb{E}_{q(\theta)}[\log h(\theta_p(s_j), \gamma_p(s_j), \xi_p(s_j))] \\
&\propto \sum_{p=1}^{P}\sum_{j=1}^{M}\left[\log\sigma(\theta_p(s_j)) - \frac{\xi_p(s_j)}{2} - \lambda(\xi_p(s_j))(\theta_p(s_j)^2 - \xi_p(s_j)^2)\right] \\
\frac{\partial Q}{\partial\xi_p(s_j)} &= \frac{1}{\sigma(\theta_p(s_j))}\sigma(\theta_p(s_j))(1-\sigma(\theta_p(s_j))) - \frac{1}{2} \\
&- \lambda'(\xi_p(s_j))[\mathbb{E}_{q(\theta)}[\theta_p(s_j)^2 - \xi_p(s_j)^2] + 2\lambda(\xi_p(s_j))\xi_p(s_j) \overset{!}{=} 0 \\
\xi_p(s_j)^{2(t+1)} &= \mathbb{E}_{q(\theta)}[\theta_p(s_j)^2] \\
&= Var(\theta_p(s_j)) + \mathbb{E}_{q(\theta)}[\theta_p(s_j)]^2
\end{aligned}$$

2.3 Evidence Lower Bound (ELBO)

$$
\begin{aligned}
\mathcal{L}(q) &= \ln\left\{\int\int\int\int\int\int q(z,\beta,\beta_0,\gamma,\theta,\Sigma^{-1})\frac{p(y,z,\beta,\beta_0,\gamma,\theta,\Sigma^{-1}|X)}{q(z,\beta,\beta_0,\gamma,\theta,\Sigma^{-1})}dzd\beta d\beta_0 d\gamma d\theta d\Sigma^{-1}\right\} \\
&= \ln\left\{\mathbb{E}_{q(z,\beta,\beta_0,\gamma,\theta,\Sigma^{-1})}\left[\frac{p(y,z,\beta,\beta_0,\gamma,\theta,\Sigma^{-1}|X)}{q(z,\beta,\beta_0,\gamma,\theta,\Sigma^{-1})}\right]\right\} \\
&\geq \mathbb{E}_{q(z,\beta,\beta_0,\gamma,\theta,\Sigma^{-1})}\left[\ln\left\{p(y,z,\beta,\beta_0,\gamma,\theta,\Sigma^{-1}|X)\right\}\right] - \\
&\quad \mathbb{E}_{q(z,\beta,\beta_0,\gamma,\theta,\Sigma^{-1})}\left[\ln\left\{q(z,\beta,\beta_0,\gamma,\theta,\Sigma^{-1})\right\}\right] \\
&= \mathbb{E}_{q(z)}\left[\ln\left\{p(y|z)\right\}\right] + \mathbb{E}_{q(z,\beta,\beta_0)}\left[\ln\left\{p(z|X,\beta,\beta_0)\right\}\right] + \mathbb{E}_{q(\beta,\gamma)}\left[\ln\left\{p(\beta|\gamma)\right\}\right] + \\
&\quad \mathbb{E}_{q(\gamma|\theta)}\left[\ln\left\{p(\gamma|\theta)\right\}\right] + \mathbb{E}_{q(\theta,\Sigma^{-1})}\left[\ln\left\{p(\theta|\Sigma^{-1})\right\}\right] + \mathbb{E}_{q(\Sigma^{-1})}\left[\ln\left\{p(\Sigma^{-1})\right\}\right] - \\
&\quad \mathbb{E}_{q(z)}\left[\ln\left\{q(z)\right\}\right] - \mathbb{E}_{q(\beta)}\left[\ln\left\{q(\beta)\right\}\right] - \mathbb{E}_{q(\beta_0)}\left[\ln\left\{q(\beta_0)\right\}\right] - \mathbb{E}_{q(\gamma)}\left[\ln\left\{q(\gamma)\right\}\right] - \\
&\quad \mathbb{E}_{q(\theta)}\left[\ln\left\{q(\theta)\right\}\right] - \mathbb{E}_{q(\Sigma^{-1})}\left[\ln\left\{q(\Sigma^{-1})\right\}\right]
\end{aligned}
$$

2.3.1 $\mathbb{E}_{q(z)}\left[\ln\left\{p(y(s_j)|z(s_j))\right\}\right]$

$$
\begin{aligned}
&\mathbb{E}_{q(z)}\left[\ln\left\{p(\boldsymbol{y}(s_j)|\boldsymbol{z}(s_j))\right\}\right] \\
&= \sum_{i=1}^{N}\mathbb{E}_{q(z_i(s_j))}\left[y_i(s_j)\ln\left\{\mathbb{1}(z_i(s_j)>0)\right\} + (1-y_i(s_j))\ln\left\{\mathbb{1}(z_i(s_j)\leq 0)\right\}\right] \\
&= \sum_{i=1}^{N}\int_{-\infty}^{\infty} q(z_i(s_j))\left\{y_i(s_j)\ln\left\{\mathbb{1}(z_i(s_j)>0)\right\} + (1-y_i(s_j))\ln\left\{\mathbb{1}(z_i(s_j)\leq 0)\right\}\right\}dz_i(s_j) \\
&= \begin{cases} \int_{-\infty}^{\infty} q(z_i(s_j))\ln\left\{\mathbb{1}(z_i(s_j)>0)\right\}dz_i(s_j), & \text{if } y_i(s_j)=1, \\ \int_{-\infty}^{\infty} q(z_i(s_j))\ln\left\{\mathbb{1}(z_i(s_j)\leq 0)\right\}dz_i(s_j), & \text{if } y_i(s_j)=0. \end{cases} \\
&= \begin{cases} \int_{-\infty}^{\infty} 0 dz_i(s_j), & \text{if } y_i(s_j)=1, \\ \int_{-\infty}^{\infty} 0 dz_i(s_j), & \text{if } y_i(s_j)=0. \end{cases} \\
&= 0
\end{aligned}
$$

2.3.2 $\mathbb{E}_{q(z,\beta,\beta_0)}[\ln\{p(z|X,\beta,\beta_0)\}]$

$$
\begin{aligned}
&\mathbb{E}_{q(z,\beta,\beta_0)}\left[\ln\left\{p(\boldsymbol{z}(s_j)|\boldsymbol{X},\boldsymbol{\beta}(s_j),\beta_0(s_j))\right\}\right] \\
&= \mathbb{E}_{q(z,\beta,\beta_0)}[\ln\{\mathcal{N}(\boldsymbol{z}(s_j);\ \boldsymbol{X}\boldsymbol{\beta}(s_j)+\beta_0(s_j),\mathrm{I})\}] \\
&= -\frac{N}{2}\ln\{2\pi\} - \frac{1}{2}\mathbb{E}_{q(z,\beta,\beta_0)}[[\boldsymbol{z}(s_j)-\boldsymbol{X}\boldsymbol{\beta}(s_j)-\beta_0(s_j)\mathbb{1}]^{\mathrm{T}}[\boldsymbol{z}(s_j)-\boldsymbol{X}\boldsymbol{\beta}(s_j)-\beta_0(s_j)\mathbb{1}]] \\
&= -\frac{N}{2}\ln\{2\pi\} - \frac{1}{2}\mathbb{E}_{q(z)}[\boldsymbol{z}(s_j)^{\mathrm{T}}\boldsymbol{z}(s_j)] + \mathbb{E}_{q(z)}[\mathbb{E}_{q(\beta)}[\boldsymbol{\beta}(s_j)^{\mathrm{T}}]\boldsymbol{X}^{\mathrm{T}}\boldsymbol{z}(s_j)] - \\
&\quad \frac{1}{2}\mathbb{E}_{q(\beta)}[\boldsymbol{\beta}(s_j)^{\mathrm{T}}\boldsymbol{X}^{\mathrm{T}}\boldsymbol{X}\boldsymbol{\beta}(s_j)] - \frac{1}{2}\mathbb{E}_{q(\beta_0)}[(\beta_0(s_j)\mathbb{1})^{\mathrm{T}}(\beta_0(s_j)\mathbb{1})] + \\
&\quad \mathbb{E}_{q(z)}[\boldsymbol{z}(s_j)^{\mathrm{T}}\mathbb{E}_{q(\beta_0)}[\beta_0(s_j)\mathbb{1}]] - \mathbb{E}_{q(\beta)}[\boldsymbol{\beta}(s_j)^{\mathrm{T}}]\boldsymbol{X}^{\mathrm{T}}\mathbb{E}_{q(\beta_0)}[\beta_0(s_j)\mathbb{1}] \\
&= -\frac{N}{2}\ln\{2\pi\} - \frac{1}{2}\mathbb{E}_{q(z)}[\boldsymbol{z}(s_j)^{\mathrm{T}}\boldsymbol{z}(s_j)] + \mathbb{E}_{q(z)}[\boldsymbol{\eta}(s_j)^{\mathrm{T}}\boldsymbol{z}(s_j)] - \\
&\quad \frac{1}{2}\mathrm{tr}(\boldsymbol{X}^{\mathrm{T}}\boldsymbol{X}\mathbb{E}_{q(\beta)}[\boldsymbol{\beta}(s_j)\boldsymbol{\beta}(s_j)^{\mathrm{T}}]) - \frac{1}{2}\mathbb{E}_{q(\beta_0)}[(\beta_0(s_j)\mathbb{1})^{\mathrm{T}}(\beta_0(s_j)\mathbb{1})] - \\
&\quad \mathbb{E}_{q(\beta)}[\boldsymbol{\beta}(s_j)^{\mathrm{T}}]\boldsymbol{X}^{\mathrm{T}}\mathbb{E}_{q(\beta_0)}[\beta_0(s_j)\mathbb{1}] \\
&= -\frac{N}{2}\ln\{2\pi\} - \frac{1}{2}\mathbb{E}_{q(z)}[\boldsymbol{z}(s_j)^{\mathrm{T}}\boldsymbol{z}(s_j)] + \mathbb{E}_{q(z)}[\boldsymbol{\eta}(s_j)^{\mathrm{T}}\boldsymbol{z}(s_j)] - \\
&\quad \frac{1}{2}\mathrm{tr}(\boldsymbol{X}^{\mathrm{T}}\boldsymbol{X}[\boldsymbol{\Sigma}_{\beta(s_j)}+\boldsymbol{\mu}_{\beta(s_j)}\boldsymbol{\mu}_{\beta(s_j)}^{\mathrm{T}}]) - \frac{1}{2}\mathbb{E}_{q(\beta_0)}[(\beta_0(s_j)\mathbb{1})^{\mathrm{T}}(\beta_0(s_j)\mathbb{1})] - \\
&\quad \mathbb{E}_{q(\beta)}[\boldsymbol{\beta}(s_j)^{\mathrm{T}}]\boldsymbol{X}^{\mathrm{T}}\mathbb{E}_{q(\beta_0)}[\beta_0(s_j)\mathbb{1}]
\end{aligned}
$$

2.3.3 $\mathbb{E}_{q(\beta,\gamma)}[\ln\{p(\beta(s_j)|\gamma(s_j))\}]$

$$
\begin{aligned}
&= \mathbb{E}_{q(\beta,\gamma)}[\ln\{p(\boldsymbol{\beta}(s_j)|\boldsymbol{\gamma}(s_j))\}] \\
&= \mathbb{E}_{q(\beta,\gamma)}[\ln\{\mathcal{N}(\boldsymbol{\beta}(s_j);\ \mathbf{0},\ \mathrm{diag}\{\nu_0(1-\gamma_p(s_j))+\nu_1\gamma_p(s_j)\}_{p=1}^{P})\}] \\
&= -\frac{P}{2}\ln\{2\pi\} - \frac{1}{2}\mathbb{E}_{q(\gamma)}[\ln\{|\mathrm{diag}\{\nu_0(1-\gamma_p(s_j))+\nu_1\gamma_p(s_j)\}_{p=1}^{P})|\}] - \\
&\quad \frac{1}{2}\mathbb{E}_{q(\beta)}[\boldsymbol{\beta}(s_j)^{\mathrm{T}}\mathbb{E}_{q(\gamma)}[\mathrm{diag}\{\nu_0(1-\gamma_p(s_j))+\nu_1\gamma_p(s_j)\}_{p=1}^{P})]^{-1}\boldsymbol{\beta}(s_j)] \\
&= -\frac{P}{2}\ln\{2\pi\} - \frac{1}{2}\mathbb{E}_{q(\gamma)}[\ln\{|\mathrm{diag}\{\nu_0(1-\gamma_p(s_j))+\nu_1\gamma_p(s_j)\}_{p=1}^{P})|\}] - \\
&\quad \frac{1}{2}\mathrm{tr}(\mathbb{E}_{q(\beta)}[\boldsymbol{\beta}(s_j)\boldsymbol{\beta}(s_j)^{\mathrm{T}}]\mathbb{E}_{q(\gamma)}[\mathrm{diag}\{\nu_0(1-\gamma_p(s_j))+\nu_1\gamma_p(s_j)\}_{p=1}^{P}]^{-1}) \\
&= -\frac{P}{2}\ln\{2\pi\} - \frac{1}{2}\mathbb{E}_{q(\gamma)}[\ln\{|\mathrm{diag}\{\nu_0(1-\gamma_p(s_j))+\nu_1\gamma_p(s_j)\}_{p=1}^{P})|\}] - \\
&\quad \frac{1}{2}\mathrm{tr}([\boldsymbol{\Sigma}_{\beta(s_j)}+\boldsymbol{\mu}_{\beta(s_j)}\boldsymbol{\mu}_{\beta(s_j)}^{\mathrm{T}}]\mathbb{E}_{q(\gamma)}[\mathrm{diag}\{\nu_0(1-\gamma_p(s_j))+\nu_1\gamma_p(s_j)\}_{p=1}^{P}]^{-1})
\end{aligned}
$$

2.3.4 $\mathbb{E}_{q(\beta_0)}[\ln\{p(\beta_0(s_j))\}]$

$$
\begin{aligned}
&\mathbb{E}_{q(\beta_0)}\left[\ln\{p(\beta_0(s_j))\}\right] \\
&= \mathbb{E}_{q(\beta_0)}\left[\ln\left\{\frac{1}{\sqrt{2\pi\sigma^2_{\beta_0}}}\exp\left\{-\frac{1}{2\sigma^2_{\beta_0}}\beta_0(s_j)^2\right\}\right\}\right] \\
&= -\frac{1}{2}\ln\{2\pi\sigma^2_{\beta_0}\} - \frac{1}{2\sigma^2_{\beta_0}}\mathbb{E}_{q(\beta_0)}[\beta_0(s_j)^2] \\
&= -\frac{1}{2}\ln\{2\pi\sigma^2_{\beta_0}\} - \frac{1}{2\sigma^2_{\beta_0}}\left[\sigma^2_{\beta_0(s_j)} + \mu^2_{\beta_0(s_j)}\right]
\end{aligned}
$$

2.3.5 $\mathbb{E}_{q(\gamma,\theta)}[\ln\{p(\gamma(s_j)|\theta(s_j))\}]$

$$
\begin{aligned}
&\mathbb{E}_{q(\gamma,\theta)}[\ln\{p(\boldsymbol{\gamma}(s_j)|\boldsymbol{\theta}(s_j))\}] \\
&= \mathbb{E}_{q(\gamma,\theta)}\left[\ln\left\{\prod_{p=1}^{P}\sigma(\theta_p(s_j))^{\gamma_p(s_j)}(1-\sigma(\theta_p(s_j)))^{(1-\gamma_p(s_j))}\right\}\right] \\
&\geq \sum_{p=1}^{P}\mathbb{E}_{q(\gamma,\theta)}\bigg[\theta_p(s_j)\gamma_p(s_j) + \ln\{\sigma(\xi_p(s_j))\} - \frac{1}{2}(\theta_p(s_j)+\xi_p(s_j)) - \\
&\quad \lambda(\xi_p(s_j))[\theta_p(s_j)^2 - \xi_p(s_j)^2]\bigg] \\
&= \sum_{p=1}^{P}\bigg\{\mathbb{E}_{q(\gamma)}[\gamma_p(s_j)]\mathbb{E}_{q(\theta)}[\theta_p(s_j)] + \ln\{\sigma(\xi_p(s_j))\} - \frac{1}{2}\mathbb{E}_{q(\theta)}[\theta_p(s_j)] - \frac{1}{2}\xi_p(s_j) - \\
&\quad \lambda(\xi_p(s_j))\mathbb{E}_{q(\theta)}[\theta_p(s_j)^2] + \lambda(\xi_p(s_j))\xi_p(s_j)^2\bigg\} \\
&= \sum_{p=1}^{P}\bigg\{\mathbb{E}_{q(\gamma)}[\gamma_p(s_j)]\mu_{\theta_p(s_j)} + \ln\{\sigma(\xi_p(s_j))\} - \frac{1}{2}\mu_{\theta_p(s_j)} - \frac{1}{2}\xi_p(s_j) - \\
&\quad \lambda(\xi_p(s_j))[\Sigma_{\theta_p(s_j)} + \mu_{\theta_p(s_j)}\mu^{\mathrm{T}}_{\theta_p(s_j)}] + \lambda(\xi_p(s_j))\xi_p(s_j)^2\bigg\}
\end{aligned}
$$

2.3.6 $\mathbb{E}_{q(\theta,\Sigma^{-1})}[\ln\{p(\theta|\Sigma^{-1})\}]$

$$
\begin{aligned}
&\mathbb{E}_{q(\theta,\Sigma^{-1})}[\ln\{p(\boldsymbol{\theta}|\boldsymbol{\Sigma}^{-1})\}]\\
&= -\frac{MP}{2}\ln\{2\pi\} + \frac{M}{2}\mathbb{E}_{q(\Sigma^{-1})}[\ln\{|\boldsymbol{\Sigma}^{-1}|\}] - \\
&\quad \frac{1}{2}\sum_{s_i\sim s_j}\mathbb{E}_{q(\theta)}[[\boldsymbol{\theta}(s_i)-\boldsymbol{\theta}(s_j)]^{\mathrm{T}}\mathbb{E}_{q(\Sigma^{-1})}[\boldsymbol{\Sigma}^{-1}][\boldsymbol{\theta}(s_i)-\boldsymbol{\theta}(s_j)]]\\
&= -\frac{MP}{2}\ln\{2\pi\} + \frac{M}{2}\mathbb{E}_{q(\Sigma^{-1})}[\ln\{|\boldsymbol{\Sigma}^{-1}|\}] - \\
&\quad \frac{1}{2}\mathrm{tr}\left(\mathbb{E}_{q(\Sigma^{-1})}\left[\boldsymbol{\Sigma}^{-1}\right]\mathbb{E}_{q(\theta)}\left[\sum_{s_i\sim s_j}[\boldsymbol{\theta}(s_i)-\boldsymbol{\theta}(s_j)][\boldsymbol{\theta}(s_i)-\boldsymbol{\theta}(s_j)]^{\mathrm{T}}\right]\right)
\end{aligned}
$$

2.3.7 $\mathbb{E}_{q(\Sigma^{-1})}[\ln\{p(\Sigma^{-1})\}]$

$$
\begin{aligned}
&\mathbb{E}_{q(\Sigma^{-1})}[\ln\{p(\boldsymbol{\Sigma}^{-1})\}]\\
&= \mathbb{E}_{q(\Sigma^{-1})}\Bigg[\frac{(\nu-P-1)}{2}\ln\{|\boldsymbol{\Sigma}^{-1}|\} - \frac{1}{2}\mathrm{tr}(\boldsymbol{I}\boldsymbol{\Sigma}^{-1}) - \frac{\nu P}{2}\ln\{2\} - \frac{P(P-1)}{4}\ln(\pi) - \\
&\quad \sum_{p=1}^{P}\ln\left\{\Gamma\left(\frac{\nu+1-p}{2}\right)\right\} - \frac{\nu}{2}\ln(|\boldsymbol{I}|)\Bigg]\\
&= \frac{(\nu-P-1)}{2}\mathbb{E}_{q(\Sigma^{-1})}[\ln\{|\boldsymbol{\Sigma}^{-1}|\}] - \frac{1}{2}\mathrm{tr}(\boldsymbol{I}\mathbb{E}_{q(\Sigma^{-1})}[\boldsymbol{\Sigma}^{-1}]) - \frac{\nu P}{2}\ln\{2\} - \frac{P(P-1)}{4}\ln(\pi) - \\
&\quad \sum_{p=1}^{P}\ln\left\{\Gamma\left(\frac{\nu+1-p}{2}\right)\right\} - \frac{\nu}{2}\ln(P)
\end{aligned}
$$

2.3.8 $\mathbb{E}_{q(z)}[\ln\{q(z(s_j))\}]$

$$
\begin{aligned}
&\mathbb{E}_{q(z)}[\ln\{q(\boldsymbol{z}(s_j))\}] \\
&= \sum_{i=1}^{N} \mathbb{E}_{q(z)} \left[\ln\left\{\mathcal{TN}_+(z_i(s_j);\ \eta_i(s_j),\ 1)^{y_i(s_j)} \mathcal{TN}_-(z_i(s_j);\ \eta_i(s_j),\ 1)^{(1-y_i(s_j))}\right\}\right] \\
&= \sum_{i=1}^{N} \mathbb{E}_{q(z)} \left[\ln\left\{\mathcal{N}(z_i(s_j);\ \eta_i(s_j),\ 1)\left[\frac{1}{1-\Phi(-\eta_i(s_j))}\right]^{y_i(s_j)} \left[\frac{1}{\Phi(-\eta_i(s_j))}\right]^{(1-y_i(s_j))}\right\}\right] \\
&= \mathbb{E}_{q(z)}\left[\ln\left\{\mathcal{N}(\boldsymbol{z}(s_j);\ \boldsymbol{\eta}(s_j),\ \boldsymbol{I})\right\}\right] - \\
&\quad \sum_{i=1}^{N}\left\{y_i(s_j)\ln\left\{1-\Phi(-\eta_i(s_j))\right\} + (1-y_i(s_j))\ln\left\{\Phi(-\eta_i(s_j))\right\}\right\} \\
&= -\frac{N}{2}\ln\{2\pi\} - \frac{1}{2}\mathbb{E}_{q(z)}\left[[\boldsymbol{z}(s_j)-\boldsymbol{\eta}(s_j)]^{\mathrm{T}}[\boldsymbol{z}(s_j)-\boldsymbol{\eta}(s_j)]\right] - \\
&\quad \sum_{i=1}^{N}\left\{y_i(s_j)\ln\left\{1-\Phi(-\eta_i(s_j))\right\} + (1-y_i(s_j))\ln\left\{\Phi(-\eta_i(s_j))\right\}\right\} \\
&= -\frac{N}{2}\ln\{2\pi\} - \frac{1}{2}\mathbb{E}_{q(z)}[\boldsymbol{z}(s_j)^{\mathrm{T}}\boldsymbol{z}(s_j)] + \mathbb{E}_{q(z)}[\boldsymbol{\eta}(s_j)^{\mathrm{T}}\boldsymbol{z}(s_j)] - \frac{1}{2}\boldsymbol{\eta}(s_j)^{\mathrm{T}}\boldsymbol{\eta}(s_j) - \\
&\quad \sum_{i=1}^{N}\left\{y_i(s_j)\ln\left\{1-\Phi(-\eta_i(s_j))\right\} + (1-y_i(s_j))\ln\left\{\Phi(-\eta_i(s_j))\right\}\right\}
\end{aligned}
$$

2.3.9 $\mathbb{E}_{q(\beta)}[\ln\{q(\beta(s_j))\}]$

$$
\begin{aligned}
&\mathbb{E}_{q(\beta)}[\ln\{q(\boldsymbol{\beta}(s_j))\}] \\
&= \mathbb{E}_{q(\beta)}[\ln\{\mathcal{N}(\boldsymbol{\beta}(s_j);\ \boldsymbol{\mu}_{\beta(s_j)},\ \boldsymbol{\Sigma}_{\beta(s_j)})\}] \\
&= -\frac{P}{2}\ln\{2\pi\} - \frac{1}{2}\ln\{|\boldsymbol{\Sigma}_{\beta(s_j)}|\} - \frac{1}{2}\mathbb{E}_{q(\beta)}\left[[\boldsymbol{\beta}(s_j)-\boldsymbol{\mu}_{\beta(s_j)}]^{\mathrm{T}}\boldsymbol{\Sigma}_{\beta(s_j)}^{-1}[\boldsymbol{\beta}(s_j)-\boldsymbol{\mu}_{\beta(s_j)}]\right] \\
&= -\frac{P}{2}\ln\{2\pi\} - \frac{1}{2}\ln\{|\boldsymbol{\Sigma}_{\beta(s_j)}|\} - \frac{1}{2}\mathbb{E}_{q(\beta)}\left[\mathrm{tr}\left(\boldsymbol{\Sigma}_{\beta(s_j)}^{-1}[\boldsymbol{\beta}(s_j)-\boldsymbol{\mu}_{\beta(s_j)}][\boldsymbol{\beta}(s_j)-\boldsymbol{\mu}_{\beta(s_j)}]^{\mathrm{T}}\right)\right] \\
&= -\frac{P}{2}\ln\{2\pi\} - \frac{1}{2}\ln\{|\boldsymbol{\Sigma}_{\beta(s_j)}|\} - \frac{1}{2}\mathrm{tr}(\boldsymbol{\Sigma}_{\beta(s_j)}^{-1}\boldsymbol{\Sigma}_{\beta(s_j)}) \\
&= -\frac{P}{2}\ln\{2\pi\} - \frac{1}{2}\ln\{|\boldsymbol{\Sigma}_{\beta(s_j)}|\} - \frac{1}{2}\mathrm{tr}(\boldsymbol{I}) \\
&= -\frac{P}{2}\ln\{2\pi\} - \frac{1}{2}\ln\{|\boldsymbol{\Sigma}_{\beta(s_j)}|\} - \frac{P}{2}
\end{aligned}
$$

2.3.10 $\mathbb{E}_{q(\beta_0)}[\ln\{q(\beta_0(s_j))\}]$

$$
\begin{aligned}
&\mathbb{E}_{q(\beta_0)}[\ln\{q(\beta_0(s_j))\}] \\
&= \mathbb{E}_{q(\beta_0)}\left[-\frac{1}{2}\ln\left\{2\pi\sigma^2_{\beta_0(s_j)}\right\} - \frac{1}{2\sigma^2_{\beta_0(s_j)}}\left(\beta_0(s_j) - \mu_{\beta_0(s_j)}\right)^2\right] \\
&= -\frac{1}{2}\ln\left\{2\pi\sigma^2_{\beta_0(s_j)}\right\} - \frac{1}{2\sigma^2_{\beta_0(s_j)}}\sigma^2_{\beta_0(s_j)} \\
&= -\frac{1}{2}\ln\left\{2\pi\sigma^2_{\beta_0(s_j)}\right\} - \frac{1}{2}
\end{aligned}
$$

2.3.11 $\mathbb{E}_{q(\gamma)}[\ln\{q(\gamma(s_j))\}]$

$$
\begin{aligned}
&\mathbb{E}_{q(\gamma)}[\ln\{q(\boldsymbol{\gamma}(s_j))\}] \\
&= \sum_{p=1}^{P}\mathbb{E}_{q(\gamma)}[\gamma_p(s_j)\ln\{\mu_{\gamma_p(s_j)}\} + (1-\gamma_p(s_j))\ln\{1-\mu_{\gamma_p(s_j)}\}] \\
&= \sum_{p=1}^{P}\left\{\mu_{\gamma_p(s_j)}\ln\{\mu_{\gamma_p(s_j)}\} + (1-\mu_{\gamma_p(s_j)})\ln\{1-\mu_{\gamma_p(s_j)}\}\right\}
\end{aligned}
$$

2.3.12 $\mathbb{E}_{q(\theta)}[\ln\{q(\theta(s_j))\}]$

$$
\begin{aligned}
&\mathbb{E}_{q(\theta)}[\ln\{q(\boldsymbol{\theta}(s_j))\}] \\
&= \mathbb{E}_{q(\theta)}\left[\ln\left\{\mathcal{N}\left(\boldsymbol{\theta}(s_j);\ \boldsymbol{\mu}_{\theta(s_j)},\ \boldsymbol{\Sigma}_{\theta(s_j)}\right)\right\}\right] \\
&= -\frac{P}{2}\ln\{2\pi\} - \frac{1}{2}\ln\left\{|\boldsymbol{\Sigma}_{\theta(s_j)}|\right\} - \frac{1}{2}\mathbb{E}_{q(\theta)}\left[[\boldsymbol{\theta}(s_j) - \boldsymbol{\mu}_{\theta(s_j)}]^{\mathrm{T}}\boldsymbol{\Sigma}^{-1}_{\theta(s_j)}[\boldsymbol{\theta}(s_j) - \boldsymbol{\mu}_{\theta(s_j)}]\right] \\
&= -\frac{P}{2}\ln\{2\pi\} - \frac{1}{2}\ln\left\{|\boldsymbol{\Sigma}_{\theta(s_j)}|\right\} - \frac{1}{2}\mathrm{tr}\left(\boldsymbol{\Sigma}^{-1}_{\theta(s_j)}\mathbb{E}_{q(\theta)}\left[[\boldsymbol{\theta}(s_j) - \boldsymbol{\mu}_{\theta(s_j)}][\boldsymbol{\theta}(s_j) - \boldsymbol{\mu}_{\theta(s_j)}]^{\mathrm{T}}\right]\right) \\
&= -\frac{P}{2}\ln\{2\pi\} - \frac{1}{2}\ln\left\{|\boldsymbol{\Sigma}_{\theta(s_j)}|\right\} - \frac{1}{2}\mathrm{tr}\left(\boldsymbol{\Sigma}^{-1}_{\theta(s_j)}\boldsymbol{\Sigma}_{\theta(s_j)}\right) \\
&= -\frac{P}{2}\ln\{2\pi\} - \frac{1}{2}\ln\left\{|\boldsymbol{\Sigma}_{\theta(s_j)}|\right\} - \frac{1}{2}\mathrm{tr}(\boldsymbol{I}) \\
&= -\frac{P}{2}\ln\{2\pi\} - \frac{1}{2}\ln\left\{|\boldsymbol{\Sigma}_{\theta(s_j)}|\right\} - \frac{P}{2}
\end{aligned}
$$

2.3.13 $\mathbb{E}_{q(\Sigma^{-1})}[\ln\{q(\Sigma^{-1})\}]$

The posterior quantities of $\boldsymbol{\Sigma}^{-1}$ are denoted by the following: posterior matrix $\boldsymbol{I}_{\Sigma^{-1}}$ and the posterior degrees of freedom $\nu_{\Sigma^{-1}}$. For the expressions of those quantities see the derivations

above.

$$
\begin{aligned}
&\mathbb{E}_{q(\Sigma^{-1})}[\ln\{q(\boldsymbol{\Sigma}^{-1})\}] \\
&= \mathbb{E}_{q(\Sigma^{-1})}\left[\frac{(\nu_{\Sigma^{-1}} - P - 1)}{2}\ln\left\{|\boldsymbol{\Sigma}^{-1}|\right\} - \frac{1}{2}\mathrm{tr}\left(\boldsymbol{\Sigma}^{-1}\boldsymbol{I}_{\Sigma^{-1}}^{-1}\right) - \frac{\nu_{\Sigma^{-1}}P}{2}\ln\{2\}\right. \\
&\left. -\frac{\nu_{\Sigma^{-1}}}{2}\ln\left\{|\boldsymbol{I}_{\Sigma^{-1}}|\right\} - \frac{P(P-1)}{4}\ln\{\pi\} - \sum_{p=1}^{P}\ln\left\{\Gamma\left(\frac{\nu_{\Sigma^{-1}}+1-p}{2}\right)\right\}\right] \\
&= \frac{(\nu_{\Sigma^{-1}} - P - 1)}{2}\mathbb{E}_{q(\Sigma^{-1})}\ln\{|\boldsymbol{\Sigma}^{-1}|\}] - \frac{1}{2}\mathrm{tr}\left(\boldsymbol{I}_{\Sigma^{-1}}^{-1}\mathbb{E}_{q(\Sigma^{-1})}[\boldsymbol{\Sigma}^{-1}]\right) - \frac{\nu_{\Sigma^{-1}}P}{2}\ln\{2\} \\
&- \frac{\nu_{\Sigma^{-1}}}{2}\ln\left\{|\boldsymbol{I}_{\Sigma^{-1}}|\right\} - \frac{P(P-1)}{4}\ln\{\pi\} - \sum_{p=1}^{P}\ln\left\{\Gamma\left(\frac{\nu_{\Sigma^{-1}}+1-p}{2}\right)\right\} \\
&= \frac{(M+\nu-P-1)}{2}\mathbb{E}_{q(\Sigma^{-1})}\ln\{|\boldsymbol{\Sigma}^{-1}|\}] - \frac{(M+\nu)P}{2} - \frac{(M+\nu)P}{2}\ln\{2\} \\
&- \frac{(M+\nu)}{2}\ln\left\{\left|\left[\boldsymbol{I} + \mathbb{E}_{q(\theta)}\left[\sum_{s_i\sim s_j}[\boldsymbol{\theta}(s_i)-\boldsymbol{\theta}(s_j)][\boldsymbol{\theta}(s_i)-\boldsymbol{\theta}(s_j)]^{\mathrm{T}}\right]\right]^{-1}\right|\right\} \\
&- \frac{P(P-1)}{4}\ln\{\pi\} - \sum_{p=1}^{P}\ln\left\{\Gamma\left(\frac{\nu_{\Sigma^{-1}}+1-p}{2}\right)\right\}
\end{aligned}
$$

2.3.14 Total ELBO

The ELBO is an alternative optimization objective which consists of the negative KL-divergence and an added constant, the logarithm of the evidence $p(\boldsymbol{y})$. Therefore, minimizing the KL-divergence is equivalent to maximizing the ELBO (Blei et al., 2017).

The evidence lower bound for BLESS is defined via Jensen's inequality (Jordan et al., 1999) by

$$\mathcal{L}(q) \geq \mathbb{E}_{q(\boldsymbol{Z},\boldsymbol{\beta},\boldsymbol{\beta_0},\boldsymbol{\gamma},\boldsymbol{\theta},\boldsymbol{\Sigma}^{-1})}\left[\ln\left\{p(\boldsymbol{Y},\boldsymbol{Z},\boldsymbol{X},\boldsymbol{\beta},\boldsymbol{\beta_0},\boldsymbol{\gamma},\boldsymbol{\theta},\boldsymbol{\Sigma}^{-1})\right\}\right] - \quad (1)$$

$$\mathbb{E}_{q(\boldsymbol{Z},\boldsymbol{\beta},\boldsymbol{\beta_0},\boldsymbol{\gamma},\boldsymbol{\theta},\boldsymbol{\Sigma}^{-1})}\left[\ln\left\{q(\boldsymbol{Z},\boldsymbol{\beta},\boldsymbol{\beta_0},\boldsymbol{\gamma},\boldsymbol{\theta},\boldsymbol{\Sigma}^{-1})\right\}\right]. \quad (2)$$

$$
\begin{aligned}
\mathcal{L}(q) = \sum_{j=1}^{M} \Bigg\{ & -\frac{N}{2}\ln\{2\pi\} - \frac{1}{2}\mathbb{E}_{q(z)}[\boldsymbol{z}(s_j)^{\mathrm{T}}\boldsymbol{z}(s_j)] + \mathbb{E}_{q(z)}[\boldsymbol{\eta}(s_j)^{\mathrm{T}}\boldsymbol{z}(s_j)] - \\
& \frac{1}{2}\mathrm{tr}\left(\boldsymbol{X}^{\mathrm{T}}\boldsymbol{X}\left[\boldsymbol{\Sigma}_{\beta(s_j)} + \boldsymbol{\mu}_{\beta(s_j)}\boldsymbol{\mu}_{\beta(s_j)}^{\mathrm{T}}\right]\right) - \frac{1}{2}\mathbb{E}_{q(\beta_0)}\left[[\beta_0(s_j)\mathbb{1}]^{\mathrm{T}}[\beta_0(s_j)\mathbb{1}]\right] - \\
& \cdot\mathbb{E}_{q(\beta)}[\boldsymbol{\beta}(s_j)^{\mathrm{T}}]\boldsymbol{X}^{\mathrm{T}}\mathbb{E}_{q(\beta_0)}[\beta_0(s_j)\mathbb{1}]\Bigg\} + \\
& \sum_{j=1}^{M}\Bigg\{-\frac{P}{2}\ln\{2\pi\} - \frac{1}{2}\sum_{p=1}^{P}\left\{\mathbb{E}_{q(\gamma)}\left[\ln\{\nu_0(1-\gamma_p(s_j)) + \nu_1\gamma_p(s_j)\}\right]\right\} - \\
& \frac{1}{2}\mathrm{tr}\left(\mathbb{E}_{q(\gamma)}\left[\mathrm{diag}\left\{\nu_0(1-\gamma_p(s_j)) + \nu_1\gamma_p(s_j)\right\}_{p=1}^{P}\right]^{-1}\left[\boldsymbol{\Sigma}_{\beta(s_j)} + \boldsymbol{\mu}_{\beta(s_j)}\boldsymbol{\mu}_{\beta(s_j)}^{\mathrm{T}}\right]\right)\Bigg\} + \\
& \sum_{j=1}^{M}\left\{-\frac{1}{2}\ln\{2\pi\sigma_{\beta_0}^2\} - \frac{1}{2\sigma_{\beta_0}^2}\left[\sigma_{\beta_0(s_j)}^2 + \mu_{\beta_0(s_j)}^2\right]\right\} + \\
& \sum_{j=1}^{M}\sum_{p=1}^{P}\Bigg\{\mathbb{E}_{q(\gamma)}[\gamma_p(s_j)]\mu_{\theta_p(s_j)} + \ln\{\sigma(\xi_p(s_j))\} - \frac{1}{2}\mu_{\theta_p(s_j)} - \frac{1}{2}\xi_p(s_j) - \\
& \cdot\lambda(\xi_p(s_j))\left[\Sigma_{\theta_p(s_j)} + \mu_{\theta_p(s_j)}\mu_{\theta_p(s_j)}^{\mathrm{T}} - \xi_p(s_j)^2\right]\Bigg\} - \frac{MP}{2}\ln\{2\pi\} + \frac{M}{2}\mathbb{E}_{q(\Sigma^{-1})}[\ln\{|\Sigma^{-1}|\}] - \\
& \frac{1}{2}\mathrm{tr}\left(\mathbb{E}_{q(\Sigma^{-1})}[\boldsymbol{\Sigma}^{-1}]\sum_{s_i\sim s_j}\mathbb{E}_{q(\theta)}\left[[\boldsymbol{\theta}(s_i)-\boldsymbol{\theta}(s_j)][\boldsymbol{\theta}(s_i)-\boldsymbol{\theta}(s_j)]^{\mathrm{T}}\right]\right) + \\
& \frac{(\nu-P-1)}{2}\mathbb{E}_{q(\Sigma^{-1})}[\ln\{|\Sigma^{-1}|\}] - \frac{1}{2}\mathrm{tr}\left(\mathbb{E}_{q(\Sigma^{-1})}[\boldsymbol{\Sigma}^{-1}]\boldsymbol{I}^{-1}\right) - \frac{\nu P}{2}\ln\{2\} - \\
& \frac{P(P-1)}{4}\ln(\pi) - \sum_{p=1}^{P}\ln\left\{\Gamma\left(\frac{\nu+1-p}{2}\right)\right\} - \frac{\nu}{2}\ln\{P\} \\
& \sum_{j=1}^{M}\Bigg\{-\frac{N}{2}\ln\{2\pi\} - \frac{1}{2}\mathbb{E}_{q(z)}[\boldsymbol{z}(s_j)^{\mathrm{T}}\boldsymbol{z}(s_j)] + \mathbb{E}_{q(z)}[\boldsymbol{\eta}(s_j)^{\mathrm{T}}\boldsymbol{z}(s_j)] - \frac{1}{2}\boldsymbol{\eta}(s_j)^{\mathrm{T}}\boldsymbol{\eta} -
\end{aligned}
$$

$$
\begin{aligned}
&\sum_{i=1}^{N}\left\{y_i(s_j)\ln\left\{1-\Phi(-\eta_i(s_j))\right\}+(1-y_i(s_j))\ln\left\{\Phi(-\eta_i(s_j))\right\}\right\}\Bigg\}-\\
&\sum_{j=1}^{M}\left\{-\frac{P}{2}\ln\{2\pi\}-\frac{1}{2}\ln\{|\boldsymbol{\Sigma}_{\beta(s_j)}|\}-\frac{P}{2}\right\}-\sum_{j=1}^{M}\left\{-\frac{1}{2}\ln\left\{2\pi\sigma^2_{\beta_0(s_j)}\right\}-\frac{1}{2}\right\}-\\
&\sum_{j=1}^{M}\sum_{p=1}^{P}\left\{\mu_{\gamma_p(s_j)}\ln\{\mu_{\gamma_p(s_j)}\}+(1-\mu_{\gamma_p(s_j)})\ln\{1-\mu_{\gamma_p(s_j)}\}\right\}-\\
&\sum_{j=1}^{M}\left\{-\frac{P}{2}\ln\{2\pi\}-\frac{1}{2}\ln\left\{|\boldsymbol{\Sigma}_{\theta(s_j)}|\right\}-\frac{P}{2}\right\}-\\
&\frac{(M+\nu-P-1)}{2}\mathbb{E}_{q(\Sigma^{-1})}\ln\{|\boldsymbol{\Sigma}^{-1}|\}\Big]+\frac{(M+\nu)P}{2}+\frac{(M+\nu)P}{2}\ln\{2\}+\\
&\frac{(M+\nu)}{2}\ln\left\{\left|\left[\boldsymbol{I}+\mathbb{E}_{q(\theta)}\left[\sum_{s_i\sim s_j}[\boldsymbol{\theta}(s_i)-\boldsymbol{\theta}(s_j)][\boldsymbol{\theta}(s_i)-\boldsymbol{\theta}(s_j)]^{\mathrm{T}}\right]\right]^{-1}\right|\right\}+\\
&\frac{P(P-1)}{4}\ln\{\pi\}+\sum_{p=1}^{P}\ln\left\{\Gamma\left(\frac{\nu_{\Sigma^{-1}}+1-p}{2}\right)\right\}
\end{aligned}
$$

2.3.15 Simplified ELBO

$$
\begin{aligned}
\mathcal{L}(q) = &-\frac{1}{2}\sum_{j=1}^{M}\operatorname{tr}\left(\boldsymbol{X}^{\mathrm{T}}\boldsymbol{X}\left[\boldsymbol{\Sigma}_{\beta(s_j)}+\boldsymbol{\mu}_{\beta(s_j)}\boldsymbol{\mu}_{\beta(s_j)}^{\mathrm{T}}\right]\right)-\frac{1}{2}\sum_{j=1}^{M}\mathbb{E}_{q(\beta_0)}\left[[\beta_0(s_j)\mathbb{1}]^{\mathrm{T}}[\beta_0(s_j)\mathbb{1}]\right]- \\
&\sum_{j=1}^{M}\mathbb{E}_{q(\beta)}[\boldsymbol{\beta}(s_j)^{\mathrm{T}}]\boldsymbol{X}^{\mathrm{T}}\mathbb{E}_{q(\beta_0)}[\beta_0(s_j)\mathbb{1}]+\frac{1}{2}\sum_{j=1}^{M}\boldsymbol{\eta}(s_j)^{\mathrm{T}}\boldsymbol{\eta}(s_j)+ \\
&\sum_{j=1}^{M}\sum_{i=1}^{N}\left\{y_i(s_j)\ln\left\{1-\Phi(-\eta_i(s_j))\right\}+(1-y_i(s_j))\ln\left\{\Phi(-\eta_i(s_j))\right\}\right\}- \\
&\frac{1}{2}\sum_{j=1}^{M}\sum_{p=1}^{P}\mathbb{E}_{q(\gamma)}[\ln\{\nu_0(1-\gamma_p(s_j))+\nu_1\gamma_p(s_j)\}]- \\
&\frac{1}{2}\sum_{j=1}^{M}\operatorname{tr}\left(\mathbb{E}_{q(\gamma)}\left[\operatorname{diag}\left\{\nu_0(1-\gamma_p(s_j))+\nu_1\gamma_p(s_j)\right\}_{p=1}^{P}\right]^{-1}\left[\boldsymbol{\Sigma}_{\beta(s_j)}+\boldsymbol{\mu}_{\beta(s_j)}\boldsymbol{\mu}_{\beta(s_j)}^{\mathrm{T}}\right]\right)+ \\
&\frac{1}{2}\sum_{j=1}^{M}\ln\left\{|\boldsymbol{\Sigma}_{\beta(s_j)}|\right\}+\frac{PM}{2}-\frac{M}{2}\ln\left\{\sigma_{\beta_0}^2\right\}-\frac{1}{2\sigma_{\beta_0}^2}\sum_{j=1}^{M}\left[\sigma_{\beta_0(s_j)}^2+\mu_{\beta_0(s_j)}^2\right]+ \\
&\frac{1}{2}\sum_{j=1}^{M}\ln\left\{\sigma_{\beta_0(s_j)}^2\right\}+\frac{M}{2}+ \\
&\sum_{j=1}^{M}\sum_{p=1}^{P}\left\{\mu_{\gamma_p(s_j)}\mu_{\theta_p(s_j)}+\ln\{\sigma(\xi_p(s_j))\}-\frac{1}{2}(\mu_{\theta_p(s_j)}+\xi_p(s_j))-\right. \\
&\left.\lambda(\xi_p(s_j))\left[\Sigma_{\theta_p(s_j)}+\mu_{\theta_p(s_j)}\mu_{\theta_p(s_j)}^{\mathrm{T}}-\xi_p(s_j)^2\right]\right\}- \\
&\sum_{j=1}^{M}\sum_{p=1}^{P}\left\{\mu_{\gamma_p(s_j)}\ln\{\mu_{\gamma_p(s_j)}\}+(1-\mu_{\gamma_p(s_j)})\ln\{1-\mu_{\gamma_p(s_j)}\}-\right. \\
&\frac{1}{2}\operatorname{tr}\left(\mathbb{E}_{q(\Sigma^{-1})}\left[\boldsymbol{\Sigma}^{-1}\right]\mathbb{E}_{q(\theta)}\left[\sum_{s_i\sim s_j}[\boldsymbol{\theta}(s_i)-\boldsymbol{\theta}(s_j)][\boldsymbol{\theta}(s_i)-\boldsymbol{\theta}(s_j)]^{\mathrm{T}}\right]\right)+ \\
&\quad\frac{1}{2}\sum_{j=1}^{M}\ln\left\{|\boldsymbol{\Sigma}_{\theta(s_j)}|\right\}+\frac{PM}{2}-\frac{1}{2}\operatorname{tr}(\boldsymbol{I}\mathbb{E}_{q(\Sigma^{-1})}[\boldsymbol{\Sigma}^{-1}])-\frac{\nu P}{2}\ln\{2\}- \\
&\quad\sum_{p=1}^{P}\ln\left\{\Gamma\left(\frac{\nu+1-p}{2}\right)\right\}-\frac{\nu}{2}\ln\{P\}+ \\
&\quad\frac{(M+\nu)P}{2}+\frac{(M+\nu)P}{2}\ln\{2\}+\sum_{p=1}^{P}\ln\left\{\Gamma\left(\frac{\nu_{\Sigma^{-1}}+1-p}{2}\right)\right\}+ \\
&\quad\frac{(M+\nu)}{2}\ln\left\{\left|\left[\boldsymbol{I}+\mathbb{E}_{q(\theta)}\left[\sum_{s_i\sim s_j}[\boldsymbol{\theta}(s_i)-\boldsymbol{\theta}(s_j)][\boldsymbol{\theta}(s_i)-\boldsymbol{\theta}(s_j)]^{\mathrm{T}}\right]\right]^{-1}\right|\right\}
\end{aligned}
$$

2.4 Log Marginal Posterior of γ

$$
\begin{aligned}
\nu_0 &= 0 \\
\boldsymbol{X}_\gamma &= \begin{cases} \boldsymbol{x}_p, & \text{if } \gamma_p = 1, \\ 0, & \text{if } \gamma_p = 0. \end{cases} \\
\boldsymbol{\beta}_\gamma &= \begin{cases} \beta_p, & \text{if } \gamma_p = 1, \\ 0, & \text{if } \gamma_p = 0. \end{cases} \\
\gamma_p &= \begin{cases} 1, & \text{if } p(\gamma_p = 1|\boldsymbol{y}) > 0.5, \\ 0, & \text{if } p(\gamma_p = 1|\boldsymbol{y}) \leq 0.5. \end{cases} \\
\boldsymbol{\eta} &= \boldsymbol{X}_\gamma \mathbb{E}_{q(\beta)}[\boldsymbol{\beta}_\gamma] + \mathbb{E}_{q(\beta_0)}[\beta_0] \\
\boldsymbol{\Sigma}_\beta &= \left[\boldsymbol{X}_\gamma^T \boldsymbol{X}_\gamma + \text{diag}\left\{ \mathbb{E}_{q(\gamma)}[\gamma_p] \frac{1}{\nu_1} \right\} \right] \\
\boldsymbol{\mu}_\beta &= \mathbb{E}_{q(\beta)}[\boldsymbol{\beta}_\gamma] \\
Q &= \text{rank}(\boldsymbol{X}_\gamma) \\
\theta &= \mathbb{E}_{q(\theta)}[\theta] \\
\Sigma^{-1} &= \mathbb{E}_{q(\Sigma^{-1})}[\Sigma^{-1}]
\end{aligned}
$$

All rows/columns where $\gamma_p(s_j) = 0$ are set to 0. Therefore, forming a sub-matrices/sub-vectors. For example, $\boldsymbol{X} \in \mathbb{R}^{N \times P}$ is represented by $\boldsymbol{X}_\gamma \in \mathbb{R}^{N \times Q}$.

$$
\begin{aligned}
\ln\{p(\boldsymbol{\gamma}|\boldsymbol{y})\} &= \ln\left\{ \int\int\int q(z, \beta, \beta_0) \frac{p(y, z, \beta, \beta_0, \gamma, \theta, \Sigma^{-1}|X)}{q(z, \beta, \beta_0)} dz d\beta d\beta_0 \right\} \\
&= \ln\left\{ \mathbb{E}_{q(z,\beta,\beta_0)} \left[\frac{p(y, z, \beta, \beta_0, \gamma, \theta, \Sigma^{-1}|X)}{q(z, \beta, \beta_0)} \right] \right\} \\
&\geq \mathbb{E}_{q(z,\beta,\beta_0)} \left[\ln\left\{ p(y, z, \beta, \beta_0, \gamma, \theta, \Sigma^{-1}|X) \right\} \right] - \mathbb{E}_{q(z,\beta,\beta_0)} \left[\ln\left\{ q(z, \beta, \beta_0) \right\} \right] \\
&= \mathbb{E}_{q(z)}[\ln\{p(y|z)\}] + \mathbb{E}_{q(z,\beta,\beta_0)}[\ln\{p(z|X, \beta, \beta_0)\}] + \mathbb{E}_{q(\beta)}[\ln\{p(\beta|\gamma)\}] \\
&+ \mathbb{E}_{q(\beta_0)}[\ln\{p(\beta_0)\}] + \ln\{p(\gamma|\theta)\} + \ln\{p(\theta|\Sigma^{-1})\} + \ln\{p(\Sigma^{-1})\} \\
&- \mathbb{E}_{q(z)}[\ln\{q(z)\}] - \mathbb{E}_{q(\beta)}[\ln\{q(\beta)\}] - \mathbb{E}_{q(\beta_0)}[\ln\{q(\beta_0)\}] \\
&\equiv \ln\{g(\boldsymbol{\gamma})\}
\end{aligned}
$$

$$
\begin{aligned}
\ln\{g(\boldsymbol{\gamma})\} =& -\frac{1}{2}\sum_{j=1}^{M} \mathrm{tr}\left(\boldsymbol{X}_{\gamma}^{T}\boldsymbol{X}_{\gamma}\left[\boldsymbol{\Sigma}_{\beta(s_j)}+\boldsymbol{\mu}_{\beta(s_j)}\boldsymbol{\mu}_{\beta(s_j)}^{T}\right]\right) - \frac{N}{2}\sum_{j=1}^{M}\mathbb{E}_{q(\beta_0)}\left[\beta_0(s_j)^2\right] \\
& - \sum_{i=1}^{N}\sum_{j=1}^{M}\mathbb{E}_{q(\beta_0)}[\beta_0(s_j)]\boldsymbol{x}_{\gamma(s_j)}^{T}\boldsymbol{\mu}_{\beta(s_j)} - \sum_{j=1}^{M}\frac{Q(s_j)}{2}\ln\{\nu_1\} \\
& - \frac{1}{2}\sum_{j=1}^{M}\mathrm{tr}\left(\mathrm{diag}\left\{\frac{1}{\nu_1}\right\}_{q=1}^{Q(s_j)}\left[\boldsymbol{\Sigma}_{\beta(s_j)}+\boldsymbol{\mu}_{\beta(s_j)}\boldsymbol{\mu}_{\beta(s_j)}^{T}\right]\right) \\
& - \frac{M}{2}\ln\left\{\sigma_{\beta_0}^2\right\} - \frac{1}{2\sigma_{\beta_0}^2}\sum_{j=1}^{M}\left[\sigma_{\beta_0(s_j)}^2+\mu_{\beta_0(s_j)}^2\right] \\
& + \sum_{j=1}^{M}\sum_{p=1}^{P}\left\{\gamma_p(s_j)\ln\{\sigma(\theta_p(s_j))\} + (1-\gamma_p(s_j))\ln\{1-\sigma(\theta_p(s_j))\}\right\} \\
& - \frac{MP}{2}\ln\{2\pi\} + \frac{M}{2}\ln\left\{|\boldsymbol{\Sigma}^{-1}|\right\} - \frac{1}{2}\mathrm{tr}\left(\boldsymbol{\Sigma}^{-1}\left[\sum_{s_i\sim s_j}[\boldsymbol{\theta}(s_i)-\boldsymbol{\theta}(s_j)][\boldsymbol{\theta}(s_i)-\boldsymbol{\theta}(s_j)]^{T}\right]\right) \\
& + \frac{\nu-P-1}{2}\ln\{|\boldsymbol{\Sigma}^{-1}|\} - \frac{1}{2}\mathrm{tr}\left(\boldsymbol{I}\boldsymbol{\Sigma}^{-1}\right) - \frac{\nu P}{2}\ln\{2\} - \frac{P(P-1)}{4}\ln\{\pi\} \\
& - \sum_{p=1}^{P}\ln\left\{\Gamma\left(\frac{\nu+1-p}{2}\right)\right\} - \frac{\nu}{2}\ln\{P\} + \frac{1}{2}\sum_{j=1}^{M}\boldsymbol{\eta}(s_j)^{T}\boldsymbol{\eta}(s_j) \\
& + \sum_{i=1}^{N}\sum_{j=1}^{M}\left\{y_i(s_j)\ln\left\{1-\Phi(-\eta_i(s_j))\right\} + (1-y_i(s_j))\ln\left\{\Phi(-\eta_i(s_j))\right\}\right\} \\
& + \frac{1}{2}\ln\left\{|\boldsymbol{\Sigma}_{\beta(s_j)}|\right\} + \sum_{j=1}^{M}\frac{Q(s_j)}{2} + \frac{1}{2}\sum_{j=1}^{M}\ln\{\sigma_{\beta_0(s_j)}^2\} + \frac{M}{2}
\end{aligned}
$$

2.5 Relevant Expectations

2.5.1 $\mathbb{E}_{q(z)}[z_i(s_j)]$

$$\mathbb{E}_{q(z)}[z_i(s_j)] = \begin{cases} \eta_i(s_j) + \frac{\phi(-\eta_i(s_j))}{1-\Phi(-\eta_i(s_j))}, & \text{if } y_i(s_j) = 1, \\ \eta_i(s_j) - \frac{\phi(-\eta_i(s_j))}{\Phi(-\eta_i(s_j))}, & \text{if } y_i(s_j) = 0. \end{cases}$$

2.5.2 $\mathbb{E}_{q(\gamma)}\left[\ln\left\{\nu_0(1-\gamma_p(s_j)) + \nu_1\gamma_p(s_j)\right\}\right]$

$$\begin{aligned}
\mathbb{E}_{q(\gamma)}\left[\ln\left\{\nu_0(1-\gamma_p(s_j)) + \nu_1\gamma_p(s_j)\right\}\right] &= \sum_{\gamma_k} q(\gamma_k)\ln\{\nu_0(1-\gamma_k) + \nu_1\gamma_k\} \\
&= q(\gamma_k = 1)\ln\{\nu_1\} + q(\gamma_k = 0)\ln\{\nu_0\} \\
&= \mathbb{E}_{q(\gamma)}[\gamma_p(s_j)]\ln\{\nu_1\} + (1 - \mathbb{E}_{q(\gamma)}[\gamma_p(s_j)])\ln\{\nu_0\} \\
&= \mu_{\gamma_p(s_j)}\ln\{\nu_1\} + (1 - \mu_{\gamma_p(s_j)})\ln\{\nu_0\}
\end{aligned}$$

2.5.3 $\mathbb{E}_{q(\gamma)}\left[\frac{1}{\nu_0(1-\gamma_p(s_j))+\nu_1\gamma_p(s_j)}\right]$

$$\begin{aligned}
\mathbb{E}_{q(\gamma)}\left[\frac{1}{\nu_0(1-\gamma_p(s_j)) + \nu_1\gamma_p(s_j)}\right] &= \sum_{\gamma_k} q(\gamma_k)\frac{1}{\nu_0(1-\gamma_k) + \nu_1\gamma_k} \\
&= \frac{q(\gamma_k = 1)}{\nu_1} + \frac{q(\gamma_k = 0)}{\nu_0} \\
&= \frac{\mathbb{E}_{q(\gamma)}[\gamma_p(s_j)]}{\nu_1} + \frac{1 - \mathbb{E}_{q(\gamma)}[\gamma_p(s_j)]}{\nu_0} \\
&= \frac{\mu_{\gamma_p(s_j)}}{\nu_1} + \frac{1 - \mu_{\gamma_p(s_j)}}{\nu_0}
\end{aligned}$$

2.6 Initialization and Convergence of Variational Approximation

The ELBO continuously increases until the algorithm converges to a local optimum when the difference in ELBO between two consecutive steps reaches a pre-determined convergence threshold ϵ. It should be noted that variational inference is also sensitive to parameter initialization considering the highly non-convex nature of the optimization problem. Hence, we choose to initialize $\boldsymbol{\beta}$ and $\boldsymbol{\beta_0}$ with estimates obtained by the mass-univariate approach Firth regression, $\boldsymbol{\gamma}$ with the fixed value 0.5, $\boldsymbol{\theta}$ with the logit transformed fraction of active voxels for every covariate from Firth regression, and $\boldsymbol{\Sigma}^{-1}$ with an identity matrix in order to ensure convergence to a local optimum.

3 Derivation of Approximate Posterior Sampling Method for BB-BLESS

3.1 Model Specification

$$\text{(1) Probit Model}\begin{cases}[y_i(s_j)|p_i(s_j)] \sim \text{Bernoulli}[p_i(s_j)] \\ \Phi^{-1}\left\{\mathbb{E}[y_i(s_j)|p_i(s_j)]\right\} = \eta_i(s_j) = \boldsymbol{x}_i^{\mathrm{T}}\boldsymbol{\beta}(s_j) + \beta_0(s_j) \\ \Phi^{-1}\left\{\Pr[y_i(s_j) = 1|\eta_i(s_j)]\right\} = \boldsymbol{x}_i^{\mathrm{T}}\boldsymbol{\beta}(s_j) + \beta_0(s_j)\end{cases}$$

$$\text{(2) Latent Model}\begin{cases}\Pr[y_i(s_j)|z_i(s_j)] = \begin{cases}1, & z_i(s_j) > 0, \\ 0, & z_i(s_j) \leq 0,\end{cases} \\ z_i(s_j) \sim \mathcal{N}(\boldsymbol{x}_i^{\mathrm{T}}\boldsymbol{\beta}(s_j) + \beta_0(s_j),\ 1)\end{cases}$$

$$\text{(3) Jitter}\begin{cases}\mu_p(s_j) \sim \mathcal{N}(0, \nu_0)\end{cases}$$

$$\text{(4) Spike-and-Slab Prior}\begin{cases}\beta_p(s_j) \sim \mathcal{N}(\mu_p(s_j),\ \nu_0(1-\gamma_p(s_j)) + \nu_1\gamma_p(s_j)) \\ \gamma_p(s_j) \sim \text{Bernoulli}(\sigma(\theta_p(s_j)))\end{cases}$$

$$\text{(5) MCAR Prior}\begin{cases}[\boldsymbol{\theta}(s_j) \mid \boldsymbol{\theta}(-s_j), \boldsymbol{\Sigma}] \sim \mathcal{N}\left(\frac{\sum_{s_r \in \partial s_j}\boldsymbol{\theta}(s_r)}{n(s_j)}, \frac{\boldsymbol{\Sigma}}{n(s_j)}\right) \\ \boldsymbol{\Sigma}^{-1} \sim \text{Wishart}(\nu,\ \boldsymbol{I})\end{cases}$$

3.2 Joint Distribution

Re-weighting of the likelihood with Dirichlet weights $\boldsymbol{w}^{(b)} \sim N \times Dir(1, \ldots, 1)$ and jitter the spike-and-slab prior by applying the mean shift $\mu_p(s_j) \sim \mathcal{N}(0, \nu_0)$, for all $p = 1, \ldots, P$ and $j = 1, \ldots, M$.

$$
\begin{aligned}
Q = & \prod_{i=1}^{N} \prod_{j=1}^{M} \Pr(y_i(s_j) | z_i(s_j))^{w_i^{(b)}} \\
& \times \prod_{i=1}^{N} \prod_{j=1}^{M} \mathcal{N}(z_i(s_j); \boldsymbol{x}_i^{\mathrm{T}} \boldsymbol{\beta}(s_j) + \beta_0(s_j), 1)^{w_i^{(b)}} \\
& \times \prod_{j=1}^{M} \mathcal{N}(\beta_0(s_j); \mu_{\beta_0}, \sigma_{\beta_0}^2) \\
& \times \prod_{j=1}^{M} \mathcal{N}(\boldsymbol{\beta}(s_j); \boldsymbol{\mu}(s_j), \mathrm{diag}\{\nu_0(1 - \gamma_p(s_j)) + \nu_1 \gamma_p(s_j)\}_{p=1}^{P}) \\
& \times \prod_{p=1}^{P} \prod_{j=1}^{M} Bernoulli(\gamma_p(s_j); \sigma(\theta_p(s_j))) \\
& \times \mathcal{N}(\boldsymbol{\theta}; \mathbf{0}, \boldsymbol{\Sigma}(\boldsymbol{D} - \boldsymbol{W})^{-1}) \\
& \times Wishart(\boldsymbol{\Sigma}^{-1}; P, \boldsymbol{I})
\end{aligned}
$$

$$
\begin{aligned}
Q^* \geq \mathbb{E}_{q(z,\beta,\beta_0,\gamma,\theta,\Sigma^{-1})} & \Bigg[-\frac{1}{2} \sum_{i=1}^{N} \sum_{j=1}^{M} w_i^{(b)} (z_i(s_j) - \boldsymbol{x}_i^{\mathrm{T}} \boldsymbol{\beta}(s_j) - \beta_0(s_j))^2 - \frac{1}{2\sigma_{\beta_0}^2} \sum_{j=1}^{M} (\beta_0(s_j) - \mu_{\beta_0})^2 \\
& - \frac{1}{2} \sum_{j=1}^{M} \log(|\mathrm{diag}\{\nu_0(1 - \gamma_p(s_j)) + \nu_1 \gamma_p(s_j)\}_{p=1}^{P}|) \\
& - \frac{1}{2} \sum_{j=1}^{M} [\boldsymbol{\beta}(s_j) - \boldsymbol{\mu}(s_j)]^{\mathrm{T}} \mathrm{diag}\{\nu_0(1 - \gamma_p(s_j)) + \nu_1 \gamma_p(s_j)\}_{p=1}^{P} [\boldsymbol{\beta}(s_j) - \boldsymbol{\mu}(s_j)] \\
& + \sum_{p=1}^{P} \sum_{j=1}^{J} \left[\log(\sigma(\xi_p(s_j))) + \theta_p(s_j)\gamma_p(s_j) - \frac{(\theta_p(s_j) + \xi_p(s_j))}{2} - \lambda(\xi_p(s_j))(\theta_p(s_j)^2 - \xi_p(s_j)^2) \right] \\
& + \frac{1}{2} \sum_{j=1}^{M} \log\left\{|\boldsymbol{\Sigma}^{-1}|\right\} - \frac{1}{2} \sum_{s_i \sim s_j} [\boldsymbol{\theta}(s_i) - \boldsymbol{\theta}(s_j)]^{\mathrm{T}} \boldsymbol{\Sigma}^{-1} [\boldsymbol{\theta}(s_i) - \boldsymbol{\theta}(s_j)] \\
& + \frac{1}{2} \left\{ (\nu - P - 1) \log\{|\boldsymbol{\Sigma}^{-1}|\} - \mathrm{tr}(\boldsymbol{\Sigma}^{-1}) \right\} \Bigg].
\end{aligned}
$$

3.3 Variational Approximations

3.3.1 Update $z_i(s_j)$

Assume $y_i(s_j) = 1$ and $\boldsymbol{\eta}(s_j) = \boldsymbol{x}_i^T \mathbb{E}_{q(\beta)}[\boldsymbol{\beta}(s_j)] + \mathbb{E}_{q(\beta_0)}[\beta_0(s_j)]$:

$$
\begin{aligned}
\ln(q^*(z_i(s_j))) &\propto w_i^{(b)} \ln\{p(y_i(s_j)|z_i(s_j))\} + w_i^{(b)} \mathbb{E}_{q(\beta,\beta_0)}\left[\ln\{p(z_i(s_j)|\boldsymbol{x}_i, \boldsymbol{\beta}(s_j), \beta_0(s_j))\}\right] \\
&\propto w_i^{(b)} y_i(s_j) \ln\left\{\mathbb{1}(z_i(s_j) > 0)\right\} + w_i^{(b)}(1 - y_i(s_j)) \ln\left\{\mathbb{1}(z_i(s_j) \le 0)\right\} \\
&- \frac{1}{2} w_i^{(b)} \mathbb{E}_{q(\beta,\beta_0)}[(z_i(s_j) - \boldsymbol{x}_i^{\mathrm{T}} \boldsymbol{\beta}(s_j) - \beta_0(s_j))^2] \\
&\propto w_i^{(b)} \ln\left\{\mathbb{1}(z_i(s_j) > 0)\right\} - \frac{1}{2} w_i^{(b)} z_i(s_j)^2 + w_i^{(b)} z_i(s_j)[\mathbb{E}_{q(\beta)}[\boldsymbol{\beta}(s_j)]^{\mathrm{T}} \boldsymbol{x}_i + \mathbb{E}_{q(\beta_0)}[\beta_0(s_j)]] \\
&\propto w_i^{(b)} \ln\left\{\mathbb{1}(z_i(s_j) > 0)\right\} - \frac{1}{2} w_i^{(b)} z_i(s_j)^2 + w_i^{(b)} z_i(s_j) \eta_i(s_j)
\end{aligned}
$$

$$
q^*(z_i(s_j)) = \begin{cases} \mathcal{TN}_+(z_i(s_j); \eta_i(s_j), 1), & \text{if } y_i(s_j) = 1, \\ \mathcal{TN}_-(z_i(s_j); \eta_i(s_j), 1), & \text{if } y_i(s_j) = 0. \end{cases}
$$

3.3.2 Update $\beta(s_j)$

$$
\begin{aligned}
\ln(q^*(\boldsymbol{\beta}(s_j))) &\propto \mathbb{E}_{q(\gamma)}[\ln\{p(\boldsymbol{\beta}(s_j)|\boldsymbol{\gamma}(s_j))\}] + \sum_i^N w_i^{(b)} \mathbb{E}_{q(z,\beta_0)}[\ln\{p(z_i(s_j)|\boldsymbol{x}_i, \boldsymbol{\beta}(s_j), \beta_0(s_j))\}] \\
&\propto -\frac{1}{2} \left[\boldsymbol{\beta}(s_j) - \boldsymbol{\mu}(s_j)\right]^{\mathrm{T}} \mathbb{E}_{q(\gamma)}[\mathrm{diag}\{\nu_0(1 - \gamma_p(s_j)) + \nu_1 \gamma_p(s_j)\}_{p=1}^P]^{-1} \left[\boldsymbol{\beta}(s_j) - \boldsymbol{\mu}(s_j)\right] \\
&- \frac{1}{2} \mathbb{E}_{q(z,\beta_0)} \left[[\boldsymbol{z}(s_j) - X\boldsymbol{\beta}(s_j) - \beta_0(s_j)]^{\mathrm{T}} \mathrm{diag}\left\{w_i^{(b)}\right\}_i^N [\boldsymbol{z}(s_j) - X\boldsymbol{\beta}(s_j) - \beta_0(s_j)] \right] \\
&\propto \mathbb{E}_{q(z,\beta_0)} \left[\boldsymbol{\beta}(s_j)^T X^T \mathrm{diag}\left\{w_i^{(b)}\right\}_i^N [\boldsymbol{z}(s_j) - \beta_0(s_j)] \right] \\
&- \frac{1}{2} \boldsymbol{\beta}(s_j)^T X^T \mathrm{diag}\left\{w_i^{(b)}\right\}_i^N X\boldsymbol{\beta}(s_j) \\
&+ \boldsymbol{\beta}(s_j)^{\mathrm{T}} \mathbb{E}_{q(\gamma)}[\mathrm{diag}\{\nu_0(1 - \gamma_p(s_j)) + \nu_1 \gamma_p(s_j)\}_{p=1}^P]^{-1} \boldsymbol{\mu}(s_j) \\
&- \frac{1}{2} \mathrm{tr}(\mathbb{E}_{q(\gamma)}[\mathrm{diag}\{\nu_0(1 - \gamma_p(s_j)) + \nu_1 \gamma_p(s_j)\}_{p=1}^P]^{-1} \boldsymbol{\beta}(s_j) \boldsymbol{\beta}(s_j)^T) \\
&\propto \boldsymbol{\beta}(s_j)^T \left[X^T \mathrm{diag}\left\{w_i^{(b)}\right\}_i^N [\mathbb{E}_{q(z}[\boldsymbol{z}(s_j)] - \mathbb{E}_{q(\beta_0)}[\beta_0(s_j)] \right. \\
&\left. + \mathbb{E}_{q(\gamma)}[\mathrm{diag}\{\nu_0(1 - \gamma_p(s_j)) + \nu_1 \gamma_p(s_j)\}_{p=1}^P]^{-1} \boldsymbol{\mu}(s_j) \right] \\
&- \frac{1}{2} \mathrm{tr}([X^T \mathrm{diag}\left\{w_i^{(b)}\right\}_i^N X + \mathbb{E}_{q(\gamma)}[\mathrm{diag}\{\nu_0(1 - \gamma_p(s_j)) + \nu_1 \gamma_p(s_j)\}_{p=1}^P]^{-1}] \boldsymbol{\beta}(s_j) \boldsymbol{\beta}(s_j)^T)
\end{aligned}
$$

$$
\begin{aligned}
q^*(\boldsymbol{\beta}(s_j)) &= \mathcal{N}(\boldsymbol{\beta}(s_j); \mu_{\beta(s_j)}, \Sigma_{\beta(s_j)}) \\
\mu_{\beta(s_j)} &= \Sigma_{\beta(s_j)} \left[X^{\mathrm{T}} \mathrm{diag} \left\{ w_i^{(b)} \right\}_i^N \left[\mathbb{E}_{q(z)}[\boldsymbol{z}(s_j)] - \mathbb{E}_{q(\beta_0)}[\beta_0(s_j)] \right] \right. \\
&\quad \left. + \mathbb{E}_{q(\gamma)}[\mathrm{diag}\{\nu_0(1-\gamma_p(s_j)) + \nu_1 \gamma_p(s_j)\}_{p=1}^P]^{-1} \boldsymbol{\mu}(s_j) \right] \\
\Sigma_{\beta(s_j)} &= \left[X^{\mathrm{T}} \mathrm{diag} \left\{ w_i^{(b)} \right\}_i^N X + \mathbb{E}_{q(\gamma)}[\mathrm{diag}\{\nu_0(1-\gamma_p(s_j)) + \nu_1 \gamma_p(s_j)\}_{p=1}^P]^{-1} \right]^{-1}
\end{aligned}
$$

3.3.3 Update $\beta_0(s_j)$

$$
\begin{aligned}
\ln(q^*(\beta_0(s_j))) &\propto \mathbb{E}_{q(z,\beta)}[\sum_i^N w_i^{(b)} \ln p(z_i(s_j)|\boldsymbol{x}_i, \beta_0(s_j), \boldsymbol{\beta}(s_j))] + \ln p(\beta_0(s_j)) \\
&\propto \sum_i^N \mathbb{E}_{q(z,\beta)}[w_i^{(b)} \ln \mathcal{N}(z_i(s_j); \boldsymbol{x}_i^{\mathrm{T}} \boldsymbol{\beta}(s_j) + \beta_0(s_j), 1)] + \ln \mathcal{N}(\beta_0(s_j); 0, \sigma_{\beta_0}^2) \\
&\propto \sum_{i=1}^N \beta_0(s_j) w_i^{(b)} [\mathbb{E}_{q(z)}[z_i(s_j)] - \boldsymbol{x}_i^T \mathbb{E}_{q(\beta)}[\boldsymbol{\beta}(s_j)]] - \frac{1}{2} \sum_i^N w_i^{(b)} \beta_0(s_j)^2 - \frac{1}{2\sigma_{\beta_0}^2} \beta_0(s_j)^2
\end{aligned}
$$

$$
\begin{aligned}
q^*(\beta_0(s_j)) &= \mathcal{N}(\beta_0(s_j); \mu_{\beta_0(s_j)}, \sigma^2_{\beta_0(s_j)}) \\
\mu_{\beta_0(s_j)} &= [\sum_i^N w_i^{(b)} + \frac{1}{\sigma_{\beta_0}^2}]^{-1} [\sum_{i=1}^N w_i^{(b)} \left[\mathbb{E}_{q(z)}[z_i(s_j)] - \boldsymbol{x}_i^T \mathbb{E}_{q(\beta)}[\boldsymbol{\beta}(s_j)] \right]] \\
\sigma^2_{\beta_0(s_j)} &= [\sum_i^n w_i^{(b)} + \frac{1}{\sigma_{\beta_0}^2}]^{-1}
\end{aligned}
$$

3.4 Other variational updates

The variational updates for the parameters $\boldsymbol{\gamma}, \boldsymbol{\theta}, \boldsymbol{\xi}, \boldsymbol{\Sigma^{-1}}$ for BB-BLESS are identical to the derived variational distributions of the standard BLESS-VI approach described in the previous section as the updates for those parameters are unaffected by the re-weighting of the likelihood and the perturbation of the prior mean in the spike-and-slab prior.

4 Additional UK Biobank Results

4.1 Pre-processing of MRI Scans

Lesions can be detected via magnetic resonance imaging (MRI) which is a non-invasive neuroimaging technique commonly used to monitor disease progression in a clinical setting or to identify biomarkers for diseases at a population level. In T2-weighted, fluid attenuated inversion recovery (FLAIR) and proton density-weighted brain images white matter lesions appear as hyperintense areas in the brain. The MRI scans however also require pre-processing in order to be utilized for the quantitative analysis of lesions. Firstly, researchers create binary lesion masks for every subject which mark the presence or absence of a lesion at a location within the brain. Lesions segmentation can either be performed manually by radiologists or automatically via procedures, such as BIANCA (Griffanti et al., 2016). Due to our interest in large-scale lesion mapping studies, such as the UK Biobank (Miller et al., 2016), fully automated algorithms are preferable and allow for a fast and reproducible method of creating lesion maps for thousands of subjects. After performing manual or automatic lesion segmentation the masks are registered from native space to a common anatomical atlas for group analysis. Spatial alignment to a standard space therefore ensures that lesion localizations are comparable across subjects and analyses (Bordin et al., 2021).

4.2 Examples of a MRI Scans Overlaid with a Contour of a Lesion Mask from a Random UK Biobank Subject

(a) FLAIR MRI Scan (b) FLAIR + Lesion Mask (c) Zoomed in Scan

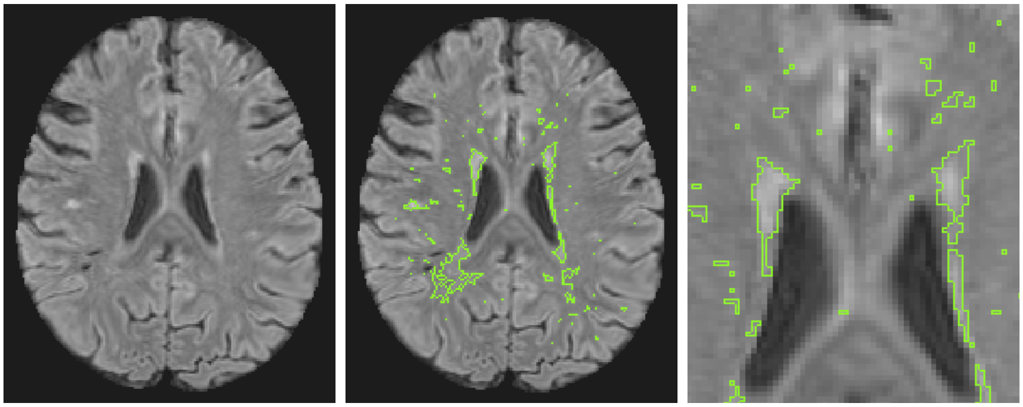

Figure 2: (a) T2-weighted FLAIR MRI scan in native space, (b) FLAIR MRI scan with overlaid contours of the respective lesion mask and (c) zoomed in version of FLAIR MRI scan with overlaid contours of a lesion mask from (b) to highlight the outlines of lesions.

4.3 Regularization and Marginal Plot for N=2,000

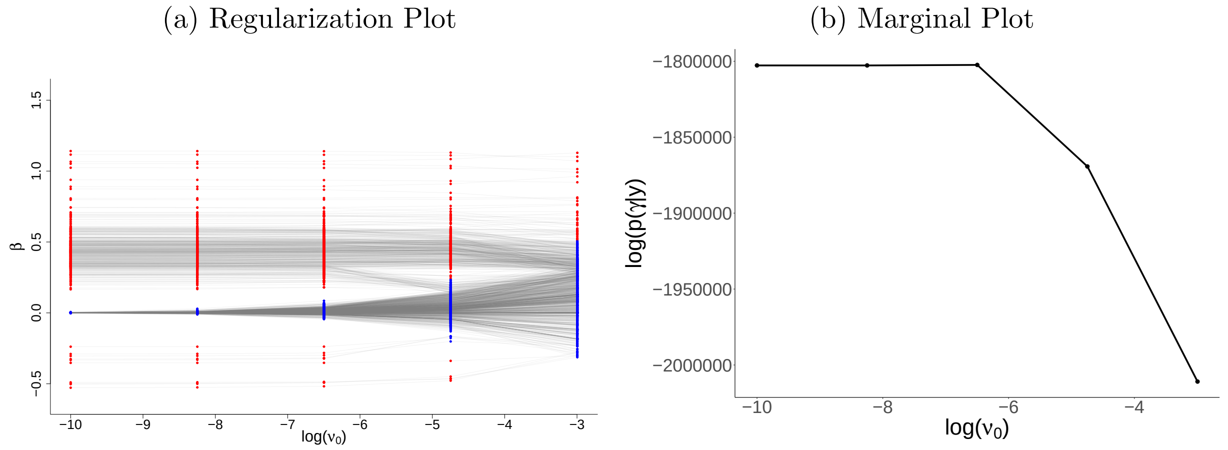

Figure 3: (a) Regularization plot for the age coefficients of an axial slice ($z = 45$, third dimension of the 3D image, plotting all $54,728$ coefficients would lead to severe overplotting) (active voxel: red, inactive voxel: blue) and (b) plot of marginal posterior of $\hat{\gamma}$ under $\nu_0 = 0$ over a sequence of equidistant $\nu_0 \in V$ within log-space ($\nu_0 = \{\exp(-10), \ldots, \exp(-3)\}$). Parameters stabilize across warm-start initializations. Small effects are shrunk to 0 (blue) and large effects are almost unregeluarized (red).

4.4 Posterior Predictive Checks

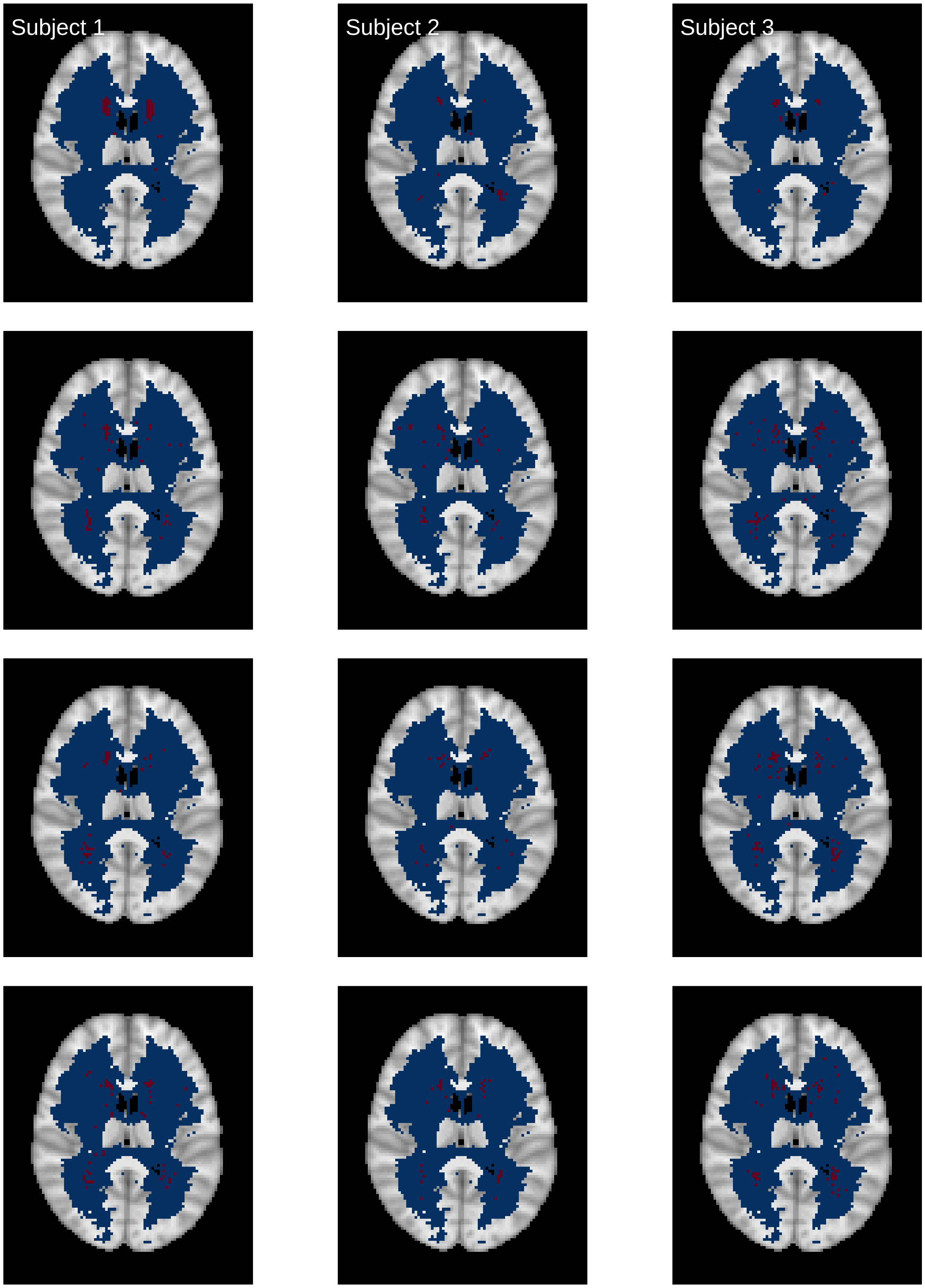

Figure 4: First row contains true lesion images and second to fourth row contains lesion masks generated via posterior predictive distribution $p(\hat{y}|y) = \int p(\hat{y}|\beta, \beta_0)p(\beta, \beta_0|y)d\beta d\beta_0$, where $\hat{y}(s_j) \sim Bernoulli(\Phi^{-1}\{\beta_0(s_j)^{(b)} + \boldsymbol{x}_i^T\boldsymbol{\beta}(s_j)^{(b)}\})$.

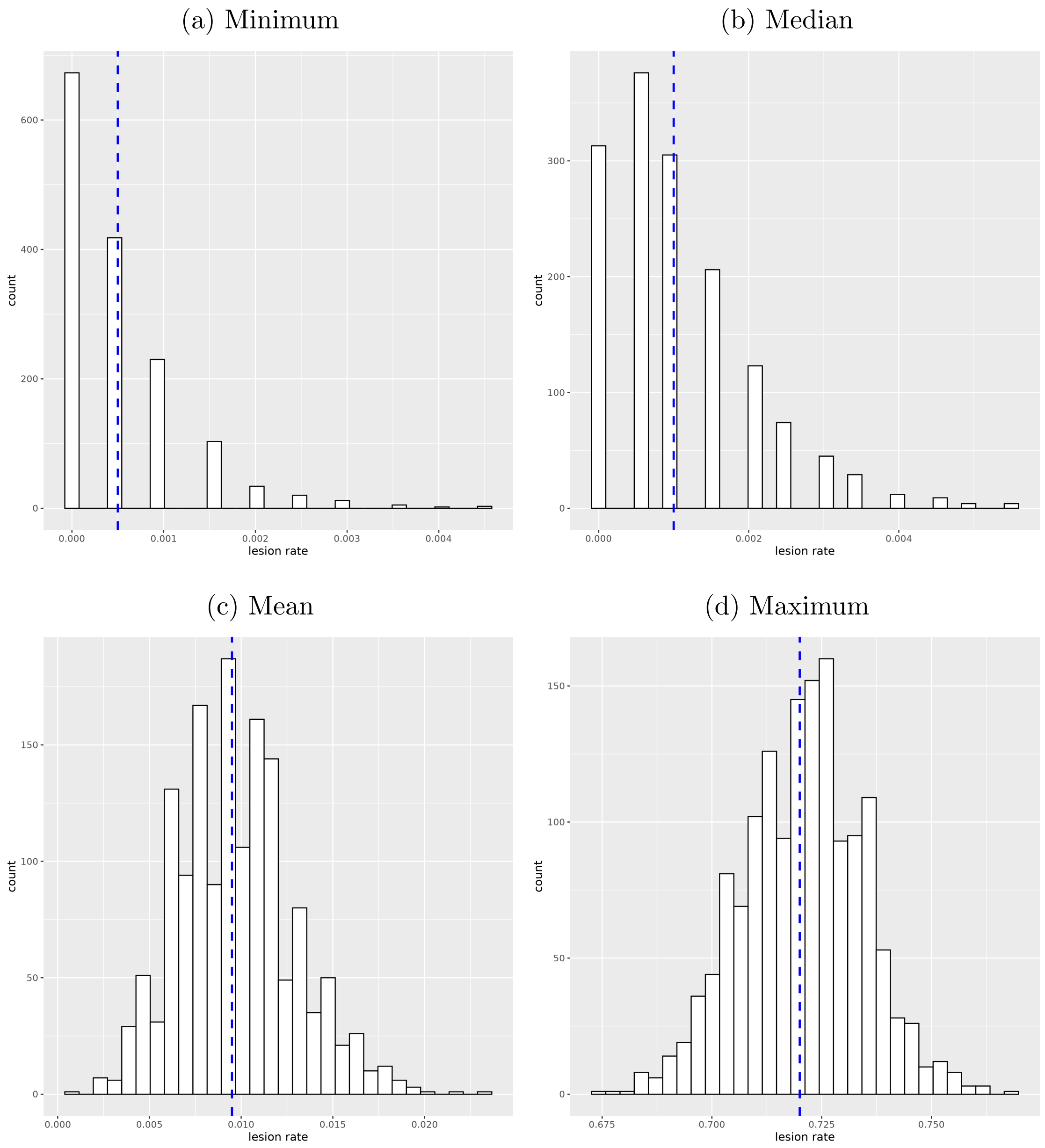

Figure 5: Lesion rate comparisons of a voxel location with the (a) minimum, (b) median, (c) mean and (d) maximum empirical lesion rate across all voxels. Blue line indicates true lesion rate at particular voxel based on dataset consisting of 2,000 subjects. Histogram is based on lesion rates based on datasets generated via posterior predictive distribution $\hat{y}(s_j) \sim Bernoulli(\Phi^{-1}\{\beta_0(s_j)^{(b)} + \boldsymbol{x}_i^T \boldsymbol{\beta}(s_j)^{(b)}\})$. The p-values $p = Pr(T(y_{rep}, \theta) \geq T(y, \theta)|y)$ are (a) 0.55133, (b) 0.54067, (c) 0.54267, and (d) 0.55, where values close to 0 or 1 indicate a poor fit. All voxel locations pass the posterior predictive check.

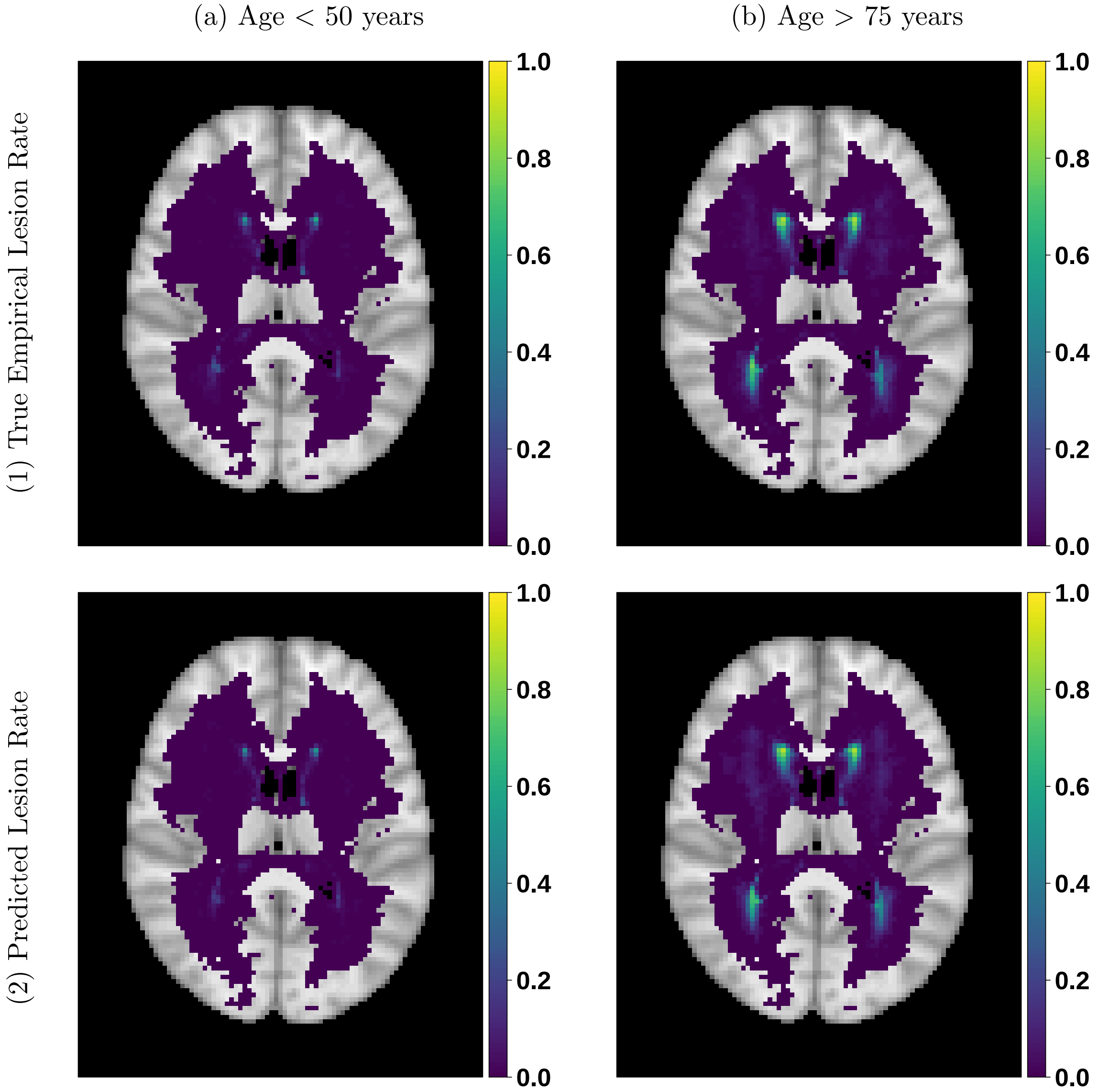

Figure 6: Comparison of (1) true empirical lesion rates to (2) predicted lesion rates (estimated via posterior predictive) of 100 held-out test subjects with an age (1) of lower than 50 years and (b) of higher than 75 years. We show that our model is able to reflect differences in lesion prevalence among different age groups on held-out test data.

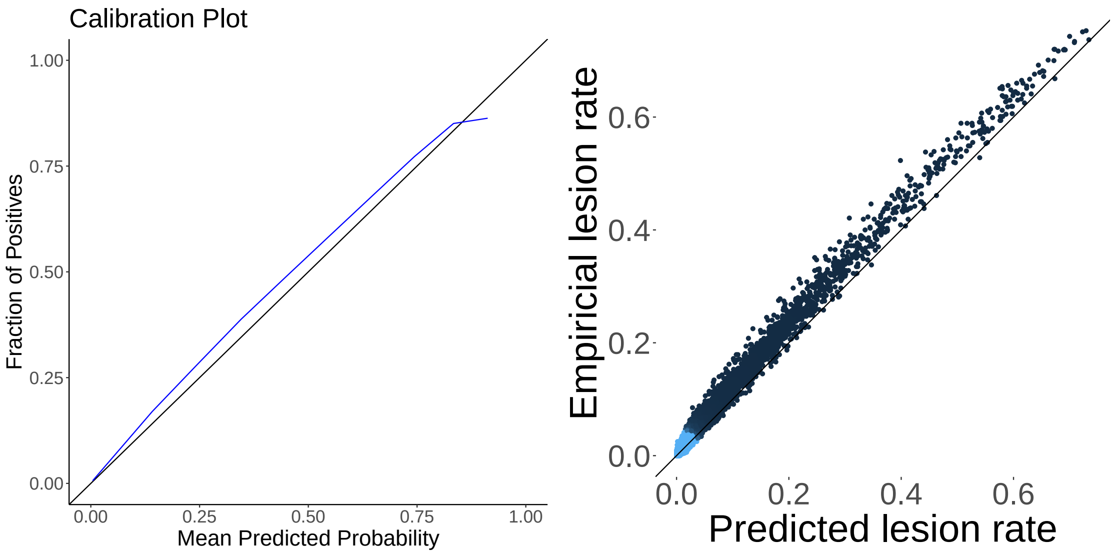

Figure 7: Calibration plot and histogram of predicted probabilities from BB-BLESS for all voxels within lesion mask for 1000 out of sample subjects. The calibration curve showcases how well the average predicted probability for binned predictions is aligned with true fraction of lesions within that bin. The scatterplot on the right of the predicted lesion probabilities by BB-BLESS against the true empirical lesion rates at each voxel highlights the same trend as the calibration plots by identifying a good alignment between predicted and empirical lesion rates with a slight overestimation of predicted lesion rates compared to the empirical lesion rates.

In this section, we evaluate the posterior predictive distribution and model calibration using out-of-sample data. We firstly assess the model fit of BLESS with a calibration plot derived from 1,000 out-of-sample subjects across all voxels within the lesion mask from the UK Biobank. Figure 7 shows that the mean predicted probability within each bin (calculated in steps of 0.1) over all voxels from BB-BLESS is well aligned with the fraction of subjects who truly exhibit a lesion over all out-of-sample subjects within a respective bin.

The scatterplot, on the right in Figure 7, of the predicted lesion probabilities by BB-BLESS against the true empirical lesion rates at each voxel for the out-of-sample dataset highlights the same trend as the calibration plot to the left. The predicted and empirical lesion rates are aligned well along the 45 degree line with a slight overestimation of predicted lesion rates compared to the empirical lesion rates. We therefore show that our model is robust to violations in model assumptions by emphasizing that BLESS is well calibrated in

its uncertainty for the finite out-of-sample predictive performance. Moreover, by ensuring that our Bayesian model is well calibrated, we show that our model has desirable frequentist properties with respect to the estimation of uncertainty for finite out-of-sample sample sizes.

4.5 Sample Size: N=40,000

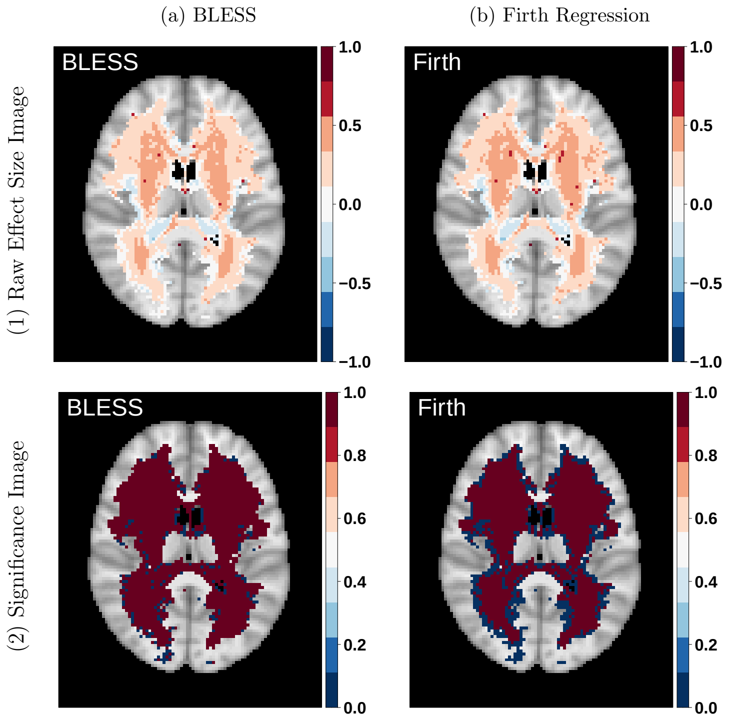

Figure 8: Comparison of results between (a) BLESS and (b) Firth Regression. (1) Spatially-varying coefficient maps of the covariate age. (2) Thresholded significance maps of the covariate age where the threshold for BLESS is determined via the probability of inclusion/exclusion $P(\gamma_p(s_j)|\hat{\boldsymbol{\beta}}, \hat{\boldsymbol{\theta}}) \geq 0.5$ and the threshold for Firth regression via the test statistic $t = |\hat{\beta}/\hat{\sigma}_{\hat{\beta}}| \geq 1.96$ (significant voxels are red, p-values have been adjusted via FDR-correction at 5%).

(a) Voxels from Slice=45 (b) Random Voxels

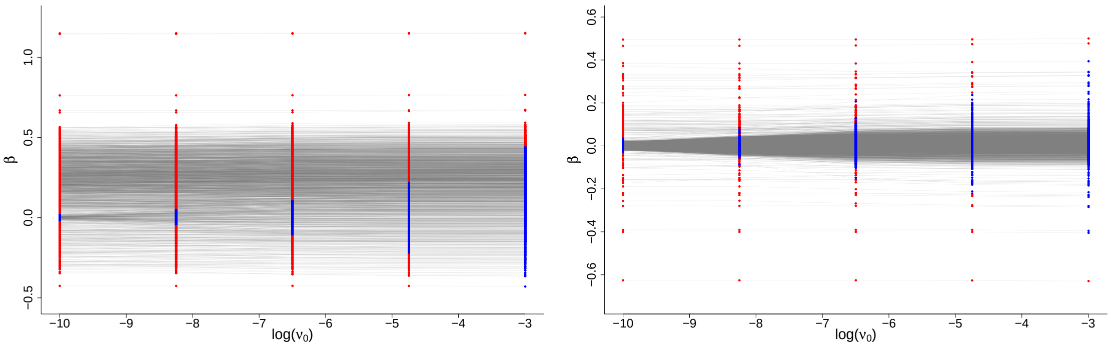

Figure 9: Regularization plots of parameters.

5 Simulation Study

5.1 Data Generating Process

The data generating mechanism is described by the following:

1) Draw the number of lesions n_ℓ for quadrant, $\ell \in \{I, II, III, IV\}$, within an image from a Poisson random variable $n_\ell \sim Poisson(\lambda c)$ where λ dictates the baserate number of lesion presence and c is the effect setting.

$$c = \begin{cases} (1,1,1,1), & \text{if } x_{gender} = 0 \text{ (male) and } x_{group} = 1, \\ (1,1,4,1), & \text{if } x_{gender} = 0 \text{ (male) and } x_{group} = 2, \\ (4,1,1,4), & \text{if } x_{gender} = 1 \text{ (female) and } x_{group} = 1, \\ (4,1,4,4), & \text{if } x_{gender} = 1 \text{ (female) and } x_{group} = 2. \end{cases}$$

2) Draw n_ℓ lesion locations uniformly within each quadrant.

3) Draw the lesion sizes from a discrete random variable, taking on lesion size $\{1, 3, 5\}$ with equal probabilities.

4) Create binary lesion masks $\boldsymbol{y}_i$ by combining the lesion locations from the 4 quadrants sampled above for every subject $i = 1, \ldots, N$.

Note that lesions are allowed to intersect with each other and merge into bigger lesion formations. In subsequent evaluations we exclude the outer two edge voxels for every quadrant in order to reduce edge effects. Figure 10 shows an example of the generated binary lesion masks for randomly selected subjects and the empirical lesion probabilities for a subset of $N = 250$ subjects for every configuration of sex and group membership at $\lambda = 3$. The overall maximum lesion intensity lies at approximately 0.25.

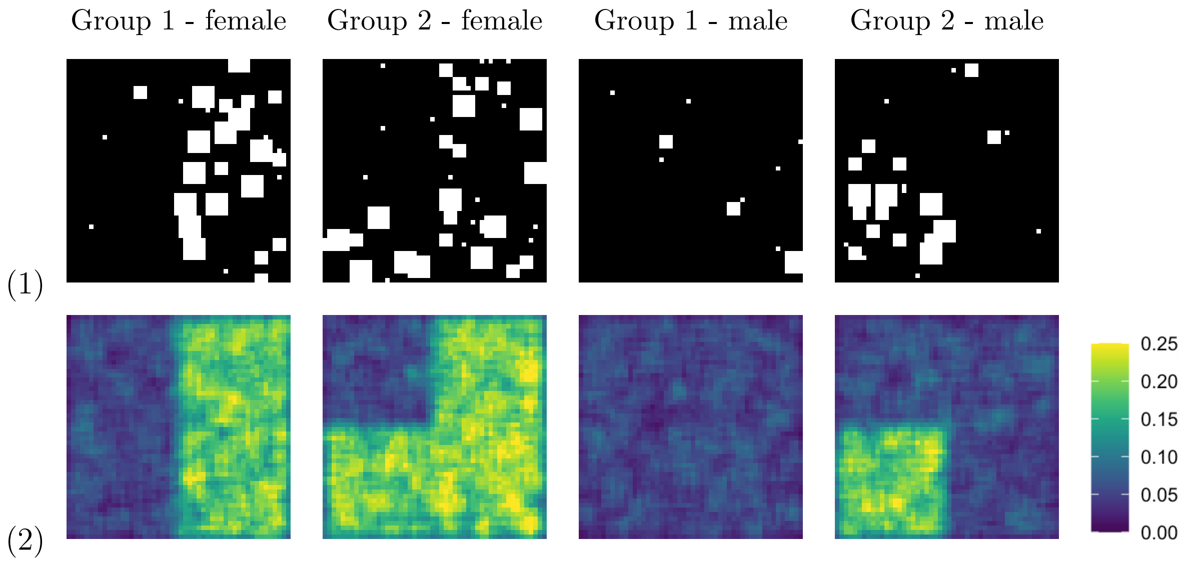

Figure 10: (1) Binary lesion masks indicating lesion presence $y_i(s_j) = 1$ and lesion absence $y_i(s_j) = 0$ for every subject $i = 1, \ldots, N$ and voxel location $j = 1, \ldots, M$ for the 4 different configurations of covariates of sex and group membership. (2) Aggregated empirical lesion maps for every combinations of male vs. female and group 1 vs. group 2.

5.2 True Parameter Estimates

We compare the point estimates of the parameter estimates $\hat{\beta}_0$, $\hat{\beta}_1$ and $\hat{\beta}_2$ to the truth where we determine truth by averaging the (homogeneous) rate within each quadrant for each of the four group-sex subject types, for very large sample size of 100,000 observations. We can acquire the true coefficient values by solving the following equation for each of the four group-sex combinations

$$\Phi^{-1}\left(p_i(s_j)\right) = \beta_0(s_j) + \beta_1(s_j)x_{i,1} + \beta_2(s_j)x_{i,2},$$

which leads to the ground truth parameter estimates $\beta_0 = -1.7933$, $\beta_1 = 0.6559$ and $\beta_2 = 0.6237$ for a base rate intensity $\lambda = 3$, for example. The other averaged true parameter values for $\hat{\beta}_0$, $\hat{\beta}_1$ and $\hat{\beta}_2$ for the intercept as well as the effects sex and group are reported in Table 1.

	β_0	β_1	β_2
$\lambda = 1$	-2.0868	0.5882	0.5586
$\lambda = 2$	-1.7933	0.6559	0.6237
$\lambda = 3$	-1.6089	0.7051	0.6685

Table 1: True parameter estimates for different base rate intensities $\lambda = 1, 2, 3$, which represent different magnitudes of lesion numbers and therefore small, medium, and large regression coefficients respectively.

5.3 Dynamic Posterior Exploration: Marginal Plot

(a) Marginal Plot ($\log\{\nu_0\} = \{-20, \ldots, -1\}$) (b) Marginal Plot ($\log\{\nu_0\} = \{-20, \ldots, -7\}$)

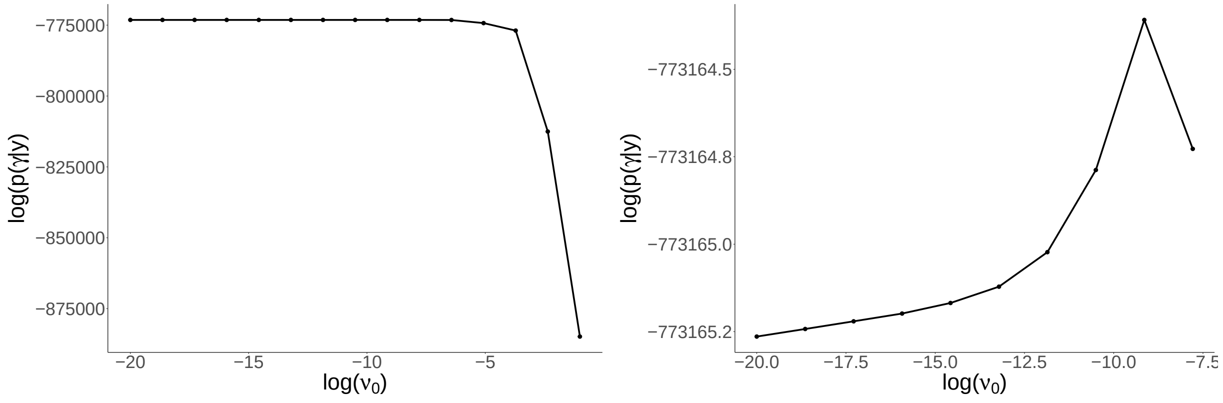

Figure 11: (a) Plot of marginal posterior of $\hat{\boldsymbol{\gamma}}$ under $\nu_0 = 0$ over a sequence of equidistant $\nu_0 \in V$ within log-space ($\nu_0 = \{\exp(-20), \ldots, \exp(-1)\}$). (b) Zoomed in range ($\nu_0 = \{\exp(-20), \ldots, \exp(-7)\}$) of the original marginal plot. Parameters stabilize across warm-start initializations and hence the marginal plot changes minimally once the coefficients have converged towards a local optimum.

6 BB-BLESS vs BLESS-Gibbs vs BLESS-VI

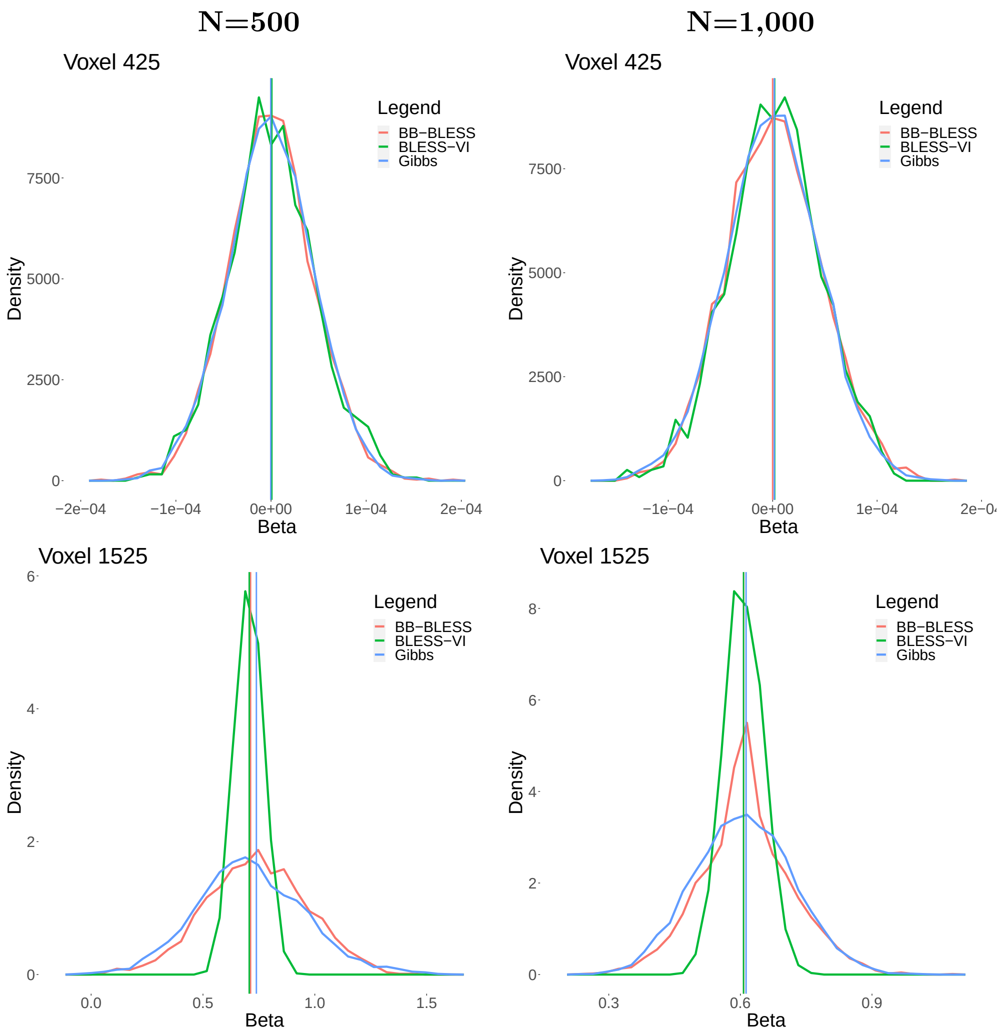

Figure 12: Comparison of marginal posterior distributions between BB-BLESS, BLESS-Gibbs and BLESS-VI where the posterior mean is indicated via a vertical line.

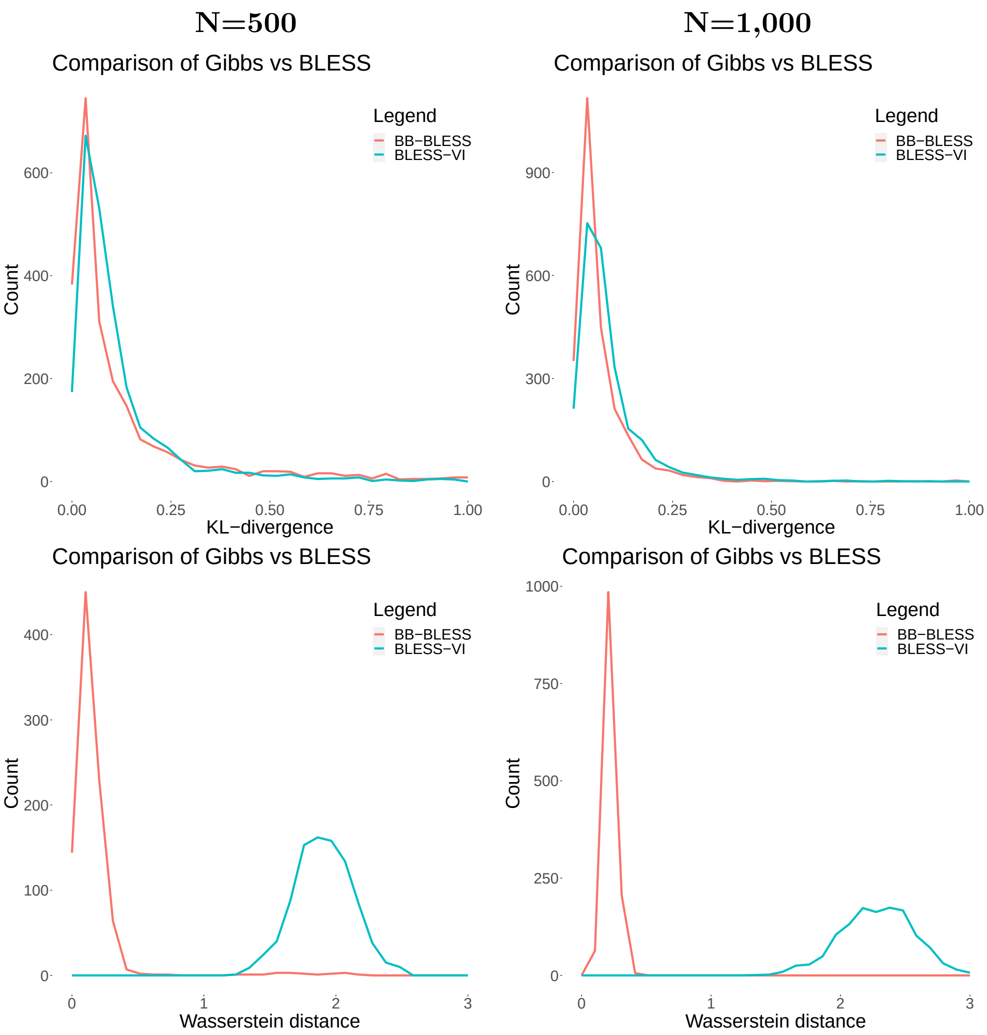

Figure 13: Evaluation of marginal posterior distribution between Gibbs and BB-BLESS and BLESS-VI via KL-divergence and Wasserstein distance.

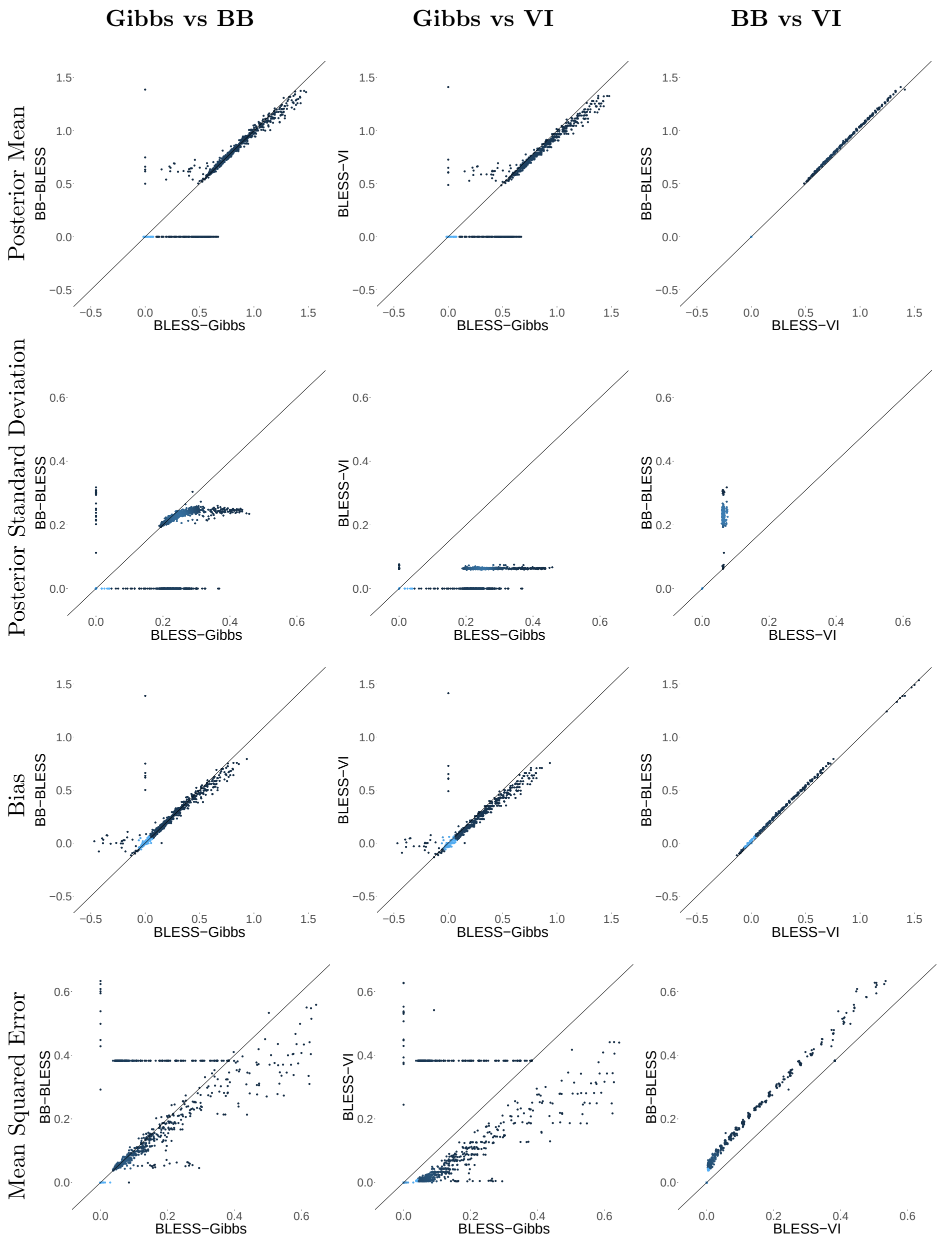

Figure 14: Comparison of posterior quantities, such as posterior mean and standard deviation, bias and mean squared error of the parameter estimates, between BB-BLESS, BLESS-Gibbs and BLESS-VI for N=500.

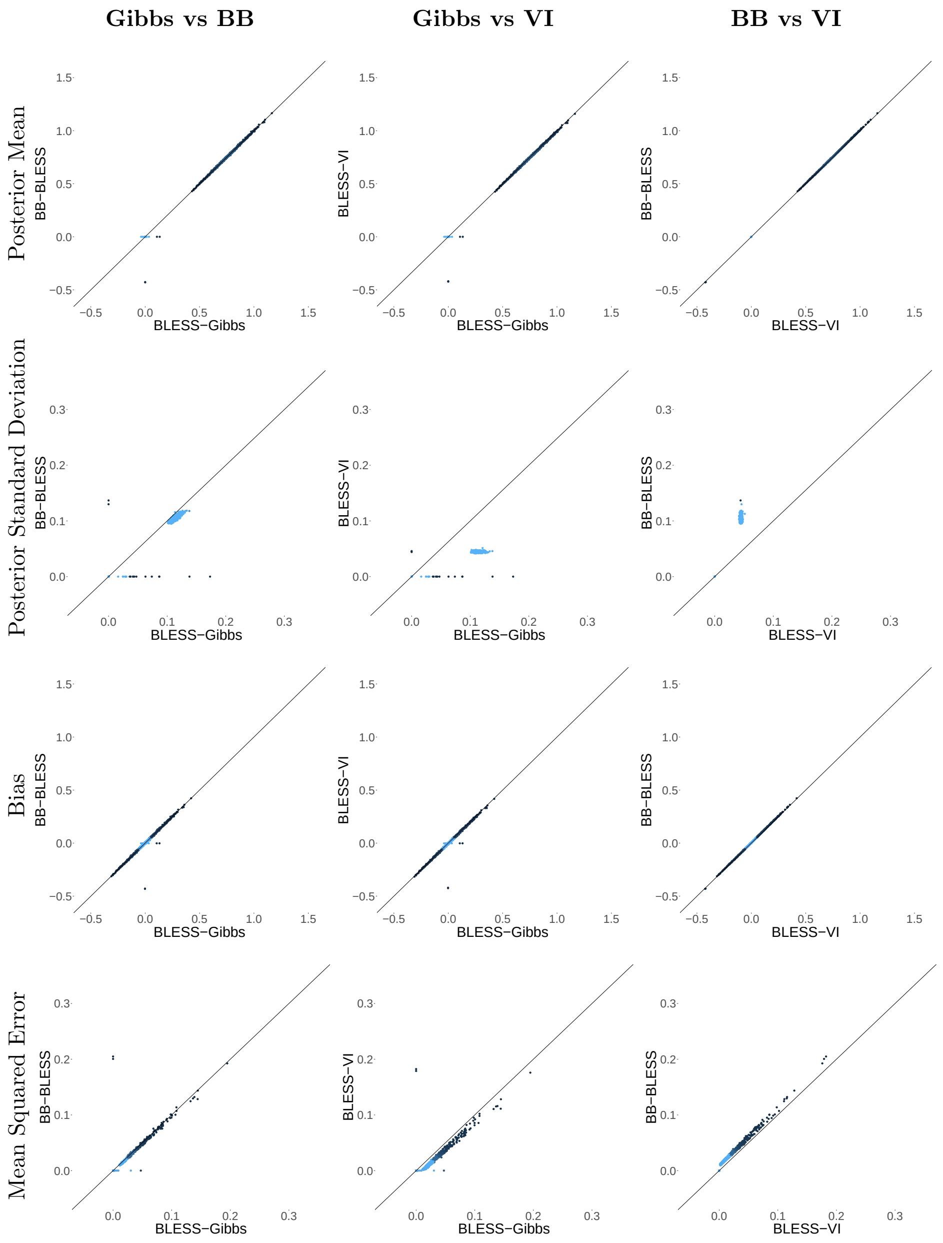

Figure 15: Comparison of posterior quantities, such as posterior mean and standard deviation, bias and mean squared error of the parameter estimates, between BB-BLESS, BLESS-Gibbs and BLESS-VI for N=$1,000$.

	$N = 500$ and $\lambda = 1$			$N = 1,000$ and $\lambda = 3$		
	β_0	β_1	β_2	β_0	β_1	β_2
Truth	-2.0868	0.5882	0.5586	-1.6089	0.7051	0.6685
BLESS-MCMC	-2.1461	0.5994	0.5802	-1.6102	0.7003	0.6517
BB-BLESS	-2.1037	0.4911	0.4848	-1.6148	0.7028	0.6622
BLESS-VI	-2.0777	0.4741	0.4685	-1.6089	0.6981	0.6576
BSGLMM	-2.1507	0.6156	0.5976	-1.6197	0.7030	0.6647
Firth	-2.1090	0.5952	0.5835	-1.6106	0.7006	0.6644

Table 2: Evaluation of parameter estimates $\hat{\boldsymbol{\beta}}_0$, $\hat{\boldsymbol{\beta}}_1$ and $\hat{\boldsymbol{\beta}}_2$ for BLESS (Gibbs, BB, VI), BSGLMM and Firth regression compared to the truth for $\boldsymbol{\beta}_0$, $\boldsymbol{\beta}_1$ and $\boldsymbol{\beta}_2$ for scenario 1 ($N = 500$ and $\lambda = 1$) and scenario 2 ($N = 1,000$ and $\lambda = 3$).

	$N = 500$ and $\lambda = 1$			$N = 1,000$ and $\lambda = 3$		
$\hat{\beta}_0$	**Bias**	**Variance**	**MSE**	**Bias**	**Variance**	**MSE**
BLESS-MCMC	-0.0701	0.0576	0.0625	-0.0094	0.0076	0.0077
BB-BLESS	-0.0210	0.0267	0.0271	-0.0122	0.0075	0.0077
BLESS-VI	0.0055	0.0020	0.0020	-0.0061	0.0010	0.0010
BSGLMM	-0.0658	0.0213	0.0256	-0.0115	0.0054	0.0055
Firth	-0.0182	0.0539	0.0542	-0.0027	0.0118	0.0118
$\hat{\beta}_1$	**Bias**	**Variance**	**MSE**	**Bias**	**Variance**	**MSE**
BLESS-MCMC	0.0288	0.0406	0.0414	0.0060	0.0065	0.0065
BB-BLESS	-0.0857	0.0171	0.0245	0.0078	0.0063	0.0064
BLESS-VI	-0.1037	0.0013	0.0121	0.0027	0.0010	0.0010
BSGLMM	0.0518	0.0120	0.0147	0.0126	0.0039	0.0040
Firth	-0.0182	0.0539	0.0542	-0.0027	0.0118	0.0118
$\hat{\beta}_2$	**Bias**	**Variance**	**MSE**	**Bias**	**Variance**	**MSE**
BLESS-MCMC	0.0441	0.0234	0.0253	0.0012	0.0032	0.0032
BB-BLESS	-0.0746	0.0092	0.0148	0.0038	0.0032	0.0032
BLESS-VI	-0.0929	0.0007	0.0093	-0.0013	0.0005	0.0005
BSGLMM	0.0364	0.0114	0.0127	-0.0038	0.0038	0.0038
Firth	-0.0182	0.0539	0.0542	-0.0027	0.0118	0.0118
$\hat{y}$	**Bias**	**Variance**	**MSE**	**Bias**	**Variance**	**MSE**
BLESS-MCMC	-0.0117	0.0007	0.0014	-0.0037	0.0007	0.0012
BB-BLESS	-0.0174	0.0011	0.0021	-0.0051	0.0007	0.0013
BLESS-VI	-0.0010	0.0011	0.0021	-0.0014	0.0007	0.0012
BSGLMM	-0.0058	0.0002	0.0002	-0.0025	0.0003	0.0003
Firth	0.0125	0.0009	0.0018	0.0051	0.0011	0.0022

Table 3: Evaluation of parameter estimates $\hat{\beta}_0$, $\hat{\beta}_1$ and $\hat{\beta}_2$ and the predictive perfomance of the estimated lesion probabilities $\hat{y}$ for BLESS (MCMC, BB, VI), BSGLMM and Firth regression via bias, variance and MSE.

	$N = 500$ and $\lambda = 1$				$N = 1,000$ and $\lambda = 3$			
$t_{\hat{\beta}_1}$	**TPR**	**TDR**	**FPR**	**FDR**	**TPR**	**TDR**	**FPR**	**FDR**
BLESS-MCMC	0.7319	0.9998	0.0001	0.0002	0.9999	0.9999	0.0001	0.0001
BB-BLESS	0.6263	0.9606	0.0257	0.0394	0.9999	0.9970	0.0031	0.0030
BLESS-VI	0.6263	0.9606	0.0257	0.0394	1.0000	0.9970	0.0031	0.0031
BSGLMM	0.9991	0.9004	0.1128	0.0996	1.0000	0.9027	0.1088	0.0973
Firth	0.6566	0.8953	0.0768	0.1047	1.0000	0.9637	0.0379	0.0363
$t_{\hat{\beta}_2}$	**TPR**	**TDR**	**FPR**	**FDR**	**TPR**	**TDR**	**FPR**	**FDR**
BLESS-MCMC	0.7376	0.9997	0.0001	0.0003	1.0000	0.9998	0.0001	0.0002
BB-BLESS	0.6478	0.9135	0.0204	0.0865	0.9998	0.9900	0.0034	0.0100
BLESS-VI	0.6479	0.9136	0.0204	0.0864	0.9998	0.9900	0.0034	0.0100
BSGLMM	0.9955	0.7664	0.1033	0.2336	1.0000	0.8093	0.0803	0.1907
Firth	0.4853	0.6625	0.0813	0.3375	1.0000	0.9181	0.0303	0.0819

Table 4: Evaluation of inference results for BLESS (MCMC, BB, VI), BSGLMM and Firth regression for two simulation study scenarios ($N = 500$, $\lambda = 1$ and $N = 1,000$, $\lambda = 3$) for the two sizes of effect wihtin the image.

7 Simulation Study: Varying Base Rate Intensities and Sample Sizes

	$\lambda = 1$			$\lambda = 2$			$\lambda = 3$		
**N=500**	β_0	β_1	β_2	β_0	β_1	β_2	β_0	β_1	β_2
Truth	-2.0868	0.5882	0.5586	-1.7933	0.6559	0.6237	-1.6089	0.7051	0.6685
BLESS	-2.0564	0.4799	0.3941	-1.7836	0.6202	0.5482	-1.6036	0.6910	0.6231
BSGLMM	-2.1314	0.6027	0.5732	-1.8168	0.6590	0.6306	-1.6233	0.7058	0.6661
Firth	-2.0864	0.5840	0.5599	-1.7925	0.6494	0.6243	-1.6059	0.6993	0.6625
**N=1,000**	β_0	β_1	β_2	β_0	β_1	β_2	β_0	β_1	β_2
Truth	-2.0868	0.5882	0.5586	-1.7933	0.6559	0.6237	-1.6089	0.7051	0.6685
BLESS	-2.0852	0.5725	0.5088	-1.7952	0.6520	0.6113	-1.6083	0.6982	0.6573
BSGLMM	-2.1066	0.5918	0.5547	-1.8056	0.6559	0.6238	-1.6182	0.7013	0.6668
Firth	-2.0836	0.5837	0.5492	-1.7925	0.6518	0.6219	-1.6091	0.6988	0.6664
**N=5,000**	β_0	β_1	β_2	β_0	β_1	β_2	β_0	β_1	β_2
Truth	-2.0868	0.5882	0.5586	-1.7933	0.6559	0.6237	-1.6089	0.7051	0.6685
BLESS	-2.0874	0.5826	0.5555	-1.7933	0.6516	0.6200	-1.6060	0.6982	0.6653
BSGLMM	-2.0913	0.5836	0.5573	-1.7965	0.6525	0.6222	-1.6087	0.6990	0.6672
Firth	-2.0861	0.5826	0.5574	-1.7934	0.6523	0.6228	-1.6065	0.6991	0.6679

Table 5: Comparison of parameter estimates from the methods, BLESS, BSGLMM and Firth Regression with true coefficient values.

**Parameter Estimate: $\hat{\boldsymbol{\beta}}_0$**	**Bias**			**Variance**			**MSE**		
**N=500**	$\lambda = 1$	$\lambda = 2$	$\lambda = 3$	$\lambda = 1$	$\lambda = 2$	$\lambda = 3$	$\lambda = 1$	$\lambda = 2$	$\lambda = 3$
BLESS	0.0242	0.0014	-0.0043	0.0020	0.0020	0.0020	0.0026	0.0020	0.0020
BSGLMM	-0.0464	-0.0243	-0.0160	0.0208	0.0131	0.0104	0.0229	0.0136	0.0106
Firth	-0.0032	-0.0001	0.0010	0.0523	0.0310	0.0238	0.0523	0.0310	0.0238
**N=1,000**	$\lambda = 1$	$\lambda = 2$	$\lambda = 3$	$\lambda = 1$	$\lambda = 2$	$\lambda = 3$	$\lambda = 1$	$\lambda = 2$	$\lambda = 3$
BLESS	-0.0054	-0.0090	-0.0059	0.0010	0.0010	0.0010	0.0010	0.0011	0.0010
BSGLMM	-0.0206	-0.0140	-0.0106	0.0102	0.0067	0.0054	0.0106	0.0069	0.0055
Firth	0.0020	-0.0014	-0.0018	0.0246	0.0152	0.0118	0.0246	0.0152	0.0118
**N=5,000**	$\lambda = 1$	$\lambda = 2$	$\lambda = 3$	$\lambda = 1$	$\lambda = 2$	$\lambda = 3$	$\lambda = 1$	$\lambda = 2$	$\lambda = 3$
BLESS	-0.0038	-0.0030	-0.0004	0.0002	0.0002	0.0002	0.0002	0.0002	0.0002
BSGLMM	-0.0056	-0.0044	-0.0015	0.0023	0.0016	0.0013	0.0023	0.0016	0.0013
Firth	-0.0006	-0.0015	0.0005	0.0048	0.0030	0.0023	0.0048	0.0030	0.0023

**Parameter Estimate: $\hat{\boldsymbol{\beta}}_1$**	**Bias**			**Variance**			**MSE**		
**N=500**	$\lambda = 1$	$\lambda = 2$	$\lambda = 3$	$\lambda = 1$	$\lambda = 2$	$\lambda = 3$	$\lambda = 1$	$\lambda = 2$	$\lambda = 3$
BLESS	-0.0961	-0.0237	-0.0009	0.0014	0.0019	0.0020	0.0106	0.0024	0.0020
BSGLMM	0.0280	0.0129	0.0130	0.0117	0.0080	0.0067	0.0125	0.0082	0.0068
Firth	0.0068	-0.0024	0.0017	0.0562	0.0348	0.0272	0.0563	0.0348	0.0272
**N=1,000**	$\lambda = 1$	$\lambda = 2$	$\lambda = 3$	$\lambda = 1$	$\lambda = 2$	$\lambda = 3$	$\lambda = 1$	$\lambda = 2$	$\lambda = 3$
BLESS	-0.0031	0.0082	0.0019	0.0010	0.0010	0.0010	0.0010	0.0011	0.0010
BSGLMM	0.0127	0.0106	0.0066	0.0063	0.0045	0.0039	0.0064	0.0046	0.0039
Firth	-0.0002	0.0026	0.0005	0.0271	0.0171	0.0135	0.0271	0.0171	0.0135
**N=5,000**	$\lambda = 1$	$\lambda = 2$	$\lambda = 3$	$\lambda = 1$	$\lambda = 2$	$\lambda = 3$	$\lambda = 1$	$\lambda = 2$	$\lambda = 3$
BLESS	0.0032	0.0039	-0.0011	0.0002	0.0002	0.0002	0.0002	0.0002	0.0002
BSGLMM	0.0057	0.0054	-0.0006	0.0018	0.0014	0.0012	0.0018	0.0014	0.0012
Firth	0.0022	0.0031	-0.0023	0.0053	0.0034	0.0027	0.0053	0.0034	0.0027

Table 6: Evaluation of parameter estimates from the methods, BLESS, BSGLMM and Firth Regression via bias, variance and MSE of the spatially-varying coefficients $\hat{\beta}_0$ and $\hat{\beta}_1$.

**Parameter Estimate: $\hat{\boldsymbol{\beta}}_2$**	**Bias**			**Variance**			**MSE**		
**N=500**	$\lambda = 1$	$\lambda = 2$	$\lambda = 3$	$\lambda = 1$	$\lambda = 2$	$\lambda = 3$	$\lambda = 1$	$\lambda = 2$	$\lambda = 3$
BLESS	-0.1661	-0.0584	-0.0284	0.0006	0.0009	0.0009	0.0282	0.0043	0.0018
BSGLMM	0.0168	0.0072	-0.0053	0.0110	0.0076	0.0064	0.0113	0.0077	0.0064
Firth	0.0040	-0.0009	-0.0102	0.0535	0.0335	0.0263	0.0535	0.0335	0.0265
**N=1,000**	$\lambda = 1$	$\lambda = 2$	$\lambda = 3$	$\lambda = 1$	$\lambda = 2$	$\lambda = 3$	$\lambda = 1$	$\lambda = 2$	$\lambda = 3$
BLESS	-0.0373	0.0058	0.0013	0.0005	0.0005	0.0005	0.0019	0.0005	0.0005
BSGLMM	-0.0034	0.0051	0.0005	0.0059	0.0043	0.0037	0.0059	0.0044	0.0037
Firth	-0.0104	0.0025	-0.0003	0.0258	0.0165	0.0131	0.0259	0.0165	0.0131
**N=5,000**	$\lambda = 1$	$\lambda = 2$	$\lambda = 3$	$\lambda = 1$	$\lambda = 2$	$\lambda = 3$	$\lambda = 1$	$\lambda = 2$	$\lambda = 3$
BLESS	-0.0002	0.0014	-0.0002	0.0001	0.0001	0.0001	0.0001	0.0001	0.0001
BSGLMM	-0.0032	-0.0002	-0.0010	0.0017	0.0013	0.0012	0.0017	0.0013	0.0012
Firth	-0.0034	0.0002	-0.0003	0.0051	0.0033	0.0026	0.0051	0.0033	0.0026

**Predictive Performance: $\hat{\boldsymbol{y}}$**	**Bias**			**Variance**			**MSE**		
**N=500**	$\lambda = 1$	$\lambda = 2$	$\lambda = 3$	$\lambda = 1$	$\lambda = 2$	$\lambda = 3$	$\lambda = 1$	$\lambda = 2$	$\lambda = 3$
BLESS	-0.0078	-0.0052	-0.0032	0.0011	0.0017	0.0018	0.0022	0.0031	0.0034
BSGLMM	-0.0027	-0.0025	-0.0020	0.0002	0.0004	0.0007	0.0002	0.0004	0.0007
Firth	0.0170	0.0140	0.0122	0.0009	0.0016	0.0022	0.0018	0.0032	0.0043
**N=1,000**	$\lambda = 1$	$\lambda = 2$	$\lambda = 3$	$\lambda = 1$	$\lambda = 2$	$\lambda = 3$	$\lambda = 1$	$\lambda = 2$	$\lambda = 3$
BLESS	-0.0015	0.0007	-0.0013	0.0004	0.0005	0.0007	0.0008	0.0010	0.0012
BSGLMM	-0.0010	-0.0018	-0.0020	0.0001	0.0002	0.0003	0.0001	0.0002	0.0003
Firth	0.0082	0.0082	0.0056	0.0005	0.0008	0.0011	0.0009	0.0016	0.0021
**N=5,000**	$\lambda = 1$	$\lambda = 2$	$\lambda = 3$	$\lambda = 1$	$\lambda = 2$	$\lambda = 3$	$\lambda = 1$	$\lambda = 2$	$\lambda = 3$
BLESS	-0.0006	-0.0000	0.0008	0.0001	0.0001	0.0001	0.0001	0.0002	0.0003
BSGLMM	-0.0004	-0.0008	-0.0001	0.0000	0.0001	0.0001	0.0000	0.0001	0.0001
Firth	0.0010	0.0015	0.0020	0.0001	0.0002	0.0002	0.0002	0.0003	0.0004

Table 7: Evaluation of parameter estimates $\hat{\beta}_2$ and the predictive performance $\hat{y}$ from the methods, BLESS, BSGLMM and Firth Regression via bias, variance and MSE.

	TPR			TDR		
**N=500**	$\lambda = 1$	$\lambda = 2$	$\lambda = 3$	$\lambda = 1$	$\lambda = 2$	$\lambda = 3$
BLESS	0.6635	0.9080	0.9770	0.9520	0.9808	0.9893
BSGLMM (uncorrected)	0.9983	1.0000	1.0000	0.8498	0.8617	0.8621
BSGLMM (corrected)	0.9972	1.0000	1.0000	0.8829	0.8941	0.8949
Firth (uncorrected)	0.8258	0.9822	0.9984	0.9553	0.9565	0.9550
Firth (corrected)	0.6600	0.9629	0.9963	0.8911	0.9368	0.9489
**N=1,000**	$\lambda = 1$	$\lambda = 2$	$\lambda = 3$	$\lambda = 1$	$\lambda = 2$	$\lambda = 3$
BLESS	0.9458	0.9983	1.0000	0.9713	0.9882	0.9936
BSGLMM (uncorrected)	1.0000	1.0000	1.0000	0.8578	0.8668	0.8773
BSGLMM (corrected)	1.0000	1.0000	1.0000	0.8906	0.8993	0.9092
Firth (uncorrected)	0.9876	0.9999	1.0000	0.9577	0.9553	0.9554
Firth (corrected)	0.9738	0.9999	1.0000	0.9406	0.9607	0.9644
**N=5,000**	$\lambda = 1$	$\lambda = 2$	$\lambda = 3$	$\lambda = 1$	$\lambda = 2$	$\lambda = 3$
BLESS	1.0000	1.0000	1.0000	0.9873	0.9973	0.9968
BSGLMM (uncorrected)	1.0000	1.0000	1.0000	0.8776	0.8956	0.8982
BSGLMM (corrected)	1.0000	1.0000	1.0000	0.9084	0.9252	0.9272
Firth (uncorrected)	1.0000	1.0000	1.0000	0.9516	0.9548	0.9515
Firth (corrected)	1.0000	1.0000	1.0000	0.9702	0.9743	0.9716
	**FPR**			**FDR**		
**N=500**	$\lambda = 1$	$\lambda = 2$	$\lambda = 3$	$\lambda = 1$	$\lambda = 2$	$\lambda = 3$
BLESS	0.0334	0.0178	0.0106	0.0480	0.0192	0.0107
BSGLMM (uncorrected)	0.1783	0.1619	0.1616	0.1502	0.1383	0.1379
BSGLMM (corrected)	0.1340	0.1196	0.1188	0.1171	0.1059	0.1051
Firth (uncorrected)	0.0388	0.0449	0.0472	0.0447	0.0435	0.0450
Firth (corrected)	0.0802	0.0652	0.0540	0.1089	0.0632	0.0511
**N=1,000**	$\lambda = 1$	$\lambda = 2$	$\lambda = 3$	$\lambda = 1$	$\lambda = 2$	$\lambda = 3$
BLESS	0.0282	0.0120	0.0065	0.0287	0.0118	0.0064
BSGLMM (uncorrected)	0.1674	0.1551	0.1410	0.1422	0.1332	0.1227
BSGLMM (corrected)	0.1243	0.1132	0.1009	0.1094	0.1007	0.0908
Firth (uncorrected)	0.0438	0.0470	0.0469	0.0423	0.0447	0.0446
Firth (corrected)	0.0619	0.0413	0.0372	0.0594	0.0393	0.0356
**N=5,000**	$\lambda = 1$	$\lambda = 2$	$\lambda = 3$	$\lambda = 1$	$\lambda = 2$	$\lambda = 3$
BLESS	0.0129	0.0027	0.0032	0.0127	0.0027	0.0032
BSGLMM (uncorrected)	0.1407	0.1175	0.1141	0.1224	0.1044	0.1018
BSGLMM (corrected)	0.1019	0.0815	0.0790	0.0916	0.0748	0.0728
Firth (uncorrected)	0.0512	0.0476	0.0511	0.0484	0.0452	0.0485
Firth (corrected)	0.0309	0.0265	0.0293	0.0298	0.0257	0.0284

Table 8: Evaluation of inference results from the methods, BLESS, BSGLMM and Firth Regression via TPR, TDR, FPR and FDR for parameter estiamte $\hat{\beta}_1$.

	TPR			TDR		
**N=500**	$\lambda = 1$	$\lambda = 2$	$\lambda = 3$	$\lambda = 1$	$\lambda = 2$	$\lambda = 3$
BLESS	0.5448	0.8336	0.9344	0.9288	0.9805	0.9930
BSGLMM (uncorrected)	0.9981	1.0000	1.0000	0.6659	0.6777	0.6889
BSGLMM (corrected)	0.9951	1.0000	1.0000	0.7871	0.7969	0.8094
Firth (uncorrected)	0.8260	0.9840	0.9978	0.8791	0.8800	0.8801
Firth (corrected)	0.4798	0.9357	0.9897	0.6342	0.8321	0.8670
**N=1,000**	$\lambda = 1$	$\lambda = 2$	$\lambda = 3$	$\lambda = 1$	$\lambda = 2$	$\lambda = 3$
BLESS	0.8888	0.9942	0.9998	0.9683	0.9909	0.9957
BSGLMM (uncorrected)	1.0000	1.0000	1.0000	0.6820	0.6883	0.7078
BSGLMM (corrected)	1.0000	1.0000	1.0000	0.8037	0.8078	0.8221
Firth (uncorrected)	0.9848	1.0000	1.0000	0.8862	0.8726	0.8733
Firth (corrected)	0.9388	0.9993	1.0000	0.8417	0.8937	0.9134
**N=5,000**	$\lambda = 1$	$\lambda = 2$	$\lambda = 3$	$\lambda = 1$	$\lambda = 2$	$\lambda = 3$
BLESS	1.0000	1.0000	1.0000	0.9851	0.9970	0.9984
BSGLMM (uncorrected)	1.0000	1.0000	1.0000	0.7222	0.7394	0.7599
BSGLMM (corrected)	1.0000	1.0000	1.0000	0.8400	0.8594	0.8763
Firth (uncorrected)	1.0000	1.0000	1.0000	0.8723	0.8710	0.8727
Firth (corrected)	1.0000	1.0000	1.0000	0.9499	0.9556	0.9565
	**FPR**			**FDR**		
**N=500**	$\lambda = 1$	$\lambda = 2$	$\lambda = 3$	$\lambda = 1$	$\lambda = 2$	$\lambda = 3$
BLESS	0.0139	0.0056	0.0022	0.0712	0.0195	0.0070
BSGLMM (uncorrected)	0.1692	0.1610	0.1523	0.3341	0.3223	0.3111
BSGLMM (corrected)	0.0914	0.0870	0.0800	0.2129	0.2031	0.1906
Firth (uncorrected)	0.0382	0.0451	0.0456	0.1209	0.1200	0.1199
Firth (corrected)	0.0908	0.0636	0.0515	0.3658	0.1679	0.1330
**N=1,000**	$\lambda = 1$	$\lambda = 2$	$\lambda = 3$	$\lambda = 1$	$\lambda = 2$	$\lambda = 3$
BLESS	0.0098	0.0031	0.0014	0.0317	0.0091	0.0043
BSGLMM (uncorrected)	0.1579	0.1527	0.1396	0.3180	0.3117	0.2922
BSGLMM (corrected)	0.0833	0.0807	0.0736	0.1963	0.1922	0.1779
Firth (uncorrected)	0.0425	0.0490	0.0488	0.1138	0.1274	0.1267
Firth (corrected)	0.0600	0.0409	0.0324	0.1583	0.1063	0.0866
**N=5,000**	$\lambda = 1$	$\lambda = 2$	$\lambda = 3$	$\lambda = 1$	$\lambda = 2$	$\lambda = 3$
BLESS	0.0051	0.0010	0.0005	0.0149	0.0030	0.0016
BSGLMM (uncorrected)	0.1298	0.1188	0.1066	0.2778	0.2606	0.2401
BSGLMM (corrected)	0.0646	0.0554	0.0477	0.1600	0.1406	0.1237
Firth (uncorrected)	0.0492	0.0497	0.0490	0.1277	0.1290	0.1273
Firth (corrected)	0.0179	0.0157	0.0153	0.0501	0.0444	0.0435

Table 9: Evaluation of inference results from the methods, BLESS, BSGLMM and Firth Regression via TPR, TDR, FPR and FDR for parameter estiamte $\hat{\beta}_2$.

8 Simulation Study: Varying Spatial Priors

	$\boldsymbol{\lambda = 1}$			$\boldsymbol{\lambda = 2}$			$\boldsymbol{\lambda = 3}$		
**N=500**	β_0	β_1	β_2	β_0	β_1	β_2	β_0	β_1	β_2
Truth	-2.0868	0.5882	0.5586	-1.7933	0.6559	0.6237	-1.6089	0.7051	0.6685
BLESS (CAR)	-1.8840	0.1218	0.1155	-1.6392	0.2978	0.2811	-1.5691	0.6123	0.5579
BLESS (PxCAR)	-2.0216	0.3912	0.3712	-1.7546	0.5469	0.5126	-1.5947	0.6693	0.6126
BLESS (MCAR)	-2.0564	0.4799	0.3941	-1.7836	0.6202	0.5482	-1.6036	0.6910	0.6231
**N=1,000**	β_0	β_1	β_2	β_0	β_1	β_2	β_0	β_1	β_2
Truth	-2.0868	0.5882	0.5586	-1.7933	0.6559	0.6237	-1.6089	0.7051	0.6685
BLESS (CAR)	-1.9952	0.3810	0.3368	-1.7900	0.6428	0.6013	-1.6061	0.6948	0.6529
BLESS (PxCAR)	-2.0559	0.5115	0.4555	-1.7916	0.6461	0.6051	-1.6065	0.6954	0.6542
BLESS (MCAR)	-2.0852	0.5725	0.5088	-1.7952	0.6520	0.6113	-1.6083	0.6982	0.6573
**N=5,000**	β_0	β_1	β_2	β_0	β_1	β_2	β_0	β_1	β_2
Truth	-2.0868	0.5882	0.5586	-1.7933	0.6559	0.6237	-1.6089	0.7051	0.6685
BLESS (CAR)	-2.0858	0.5807	0.5514	-1.7928	0.6508	0.6183	-1.6055	0.6977	0.6637
BLESS (PxCAR)	-2.0859	0.5809	0.5520	-1.7928	0.6509	0.6186	-1.6056	0.6977	0.6639
BLESS (MCAR)	-2.0874	0.5826	0.5555	-1.7933	0.6516	0.6200	-1.6060	0.6982	0.6653

Table 10: Comparison of parameter estimates from the methods, BLESS with three different types of spatial prior, specifically three formulations of a conditional autoregressive prior, BSGLMM and Firth Regression with true coefficient values.

**Parameter Estimate: $\hat{\boldsymbol{\beta}}_0$**		**Bias**			**Variance**			**MSE**	
**N=500**	$\lambda=1$	$\lambda=2$	$\lambda=3$	$\lambda=1$	$\lambda=2$	$\lambda=3$	$\lambda=1$	$\lambda=2$	$\lambda=3$
BLESS (CAR)	0.2076	0.1523	0.0283	-0.4831	-0.3658	-0.0789	-0.4855	-0.3560	-0.0934
BLESS (PxCAR)	0.0601	0.0294	0.0021	-0.1909	-0.0983	-0.0194	-0.1925	-0.0943	-0.0344
BLESS (MCAR)	0.0242	0.0014	-0.0043	-0.0961	-0.0237	-0.0009	-0.1661	-0.0584	-0.0284
**N=1,000**	$\lambda=1$	$\lambda=2$	$\lambda=3$	$\lambda=1$	$\lambda=2$	$\lambda=3$	$\lambda=1$	$\lambda=2$	$\lambda=3$
BLESS (CAR)	0.0862	-0.0065	-0.0055	-0.2035	0.0027	0.0013	-0.2238	0.0016	0.0010
BLESS (PxCAR)	0.0217	-0.0078	-0.0056	-0.0641	0.0057	0.0014	-0.0884	0.0045	0.0011
BLESS (MCAR)	-0.0054	-0.0090	-0.0059	-0.0031	0.0082	0.0019	-0.0373	0.0058	0.0013
**N=5,000**	$\lambda=1$	$\lambda=2$	$\lambda=3$	$\lambda=1$	$\lambda=2$	$\lambda=3$	$\lambda=1$	$\lambda=2$	$\lambda=3$
BLESS (CAR)	-0.0036	-0.0030	-0.0004	0.0030	0.0038	-0.0011	-0.0002	0.0014	-0.0002
BLESS (PxCAR)	-0.0036	-0.0030	-0.0004	0.0030	0.0038	-0.0011	-0.0002	0.0014	-0.0002
BLESS (MCAR)	-0.0038	-0.0030	-0.0004	0.0032	0.0039	-0.0011	-0.0002	0.0014	-0.0002

**Parameter Estimate: $\hat{\boldsymbol{\beta}}_1$**		**Bias**			**Variance**			**MSE**	
**N=500**	$\lambda=1$	$\lambda=2$	$\lambda=3$	$\lambda=1$	$\lambda=2$	$\lambda=3$	$\lambda=1$	$\lambda=2$	$\lambda=3$
BLESS (CAR)	0.0020	0.0020	0.0020	0.0002	0.0007	0.0017	0.0001	0.0004	0.0008
BLESS (PxCAR)	0.0020	0.0020	0.0020	0.0010	0.0015	0.0019	0.0005	0.0008	0.0009
BLESS (MCAR)	0.0020	0.0020	0.0020	0.0014	0.0019	0.0020	0.0006	0.0009	0.0009
**N=1,000**	$\lambda=1$	$\lambda=2$	$\lambda=3$	$\lambda=1$	$\lambda=2$	$\lambda=3$	$\lambda=1$	$\lambda=2$	$\lambda=3$
BLESS (CAR)	0.0010	0.0010	0.0010	0.0006	0.0010	0.0010	0.0003	0.0005	0.0005
BLESS (PxCAR)	0.0010	0.0010	0.0010	0.0008	0.0010	0.0010	0.0004	0.0005	0.0005
BLESS (MCAR)	0.0010	0.0010	0.0010	0.0010	0.0010	0.0010	0.0005	0.0005	0.0005
**N=5,000**	$\lambda=1$	$\lambda=2$	$\lambda=3$	$\lambda=1$	$\lambda=2$	$\lambda=3$	$\lambda=1$	$\lambda=2$	$\lambda=3$
BLESS (CAR)	0.0002	0.0002	0.0002	0.0002	0.0002	0.0002	0.0001	0.0001	0.0001
BLESS (PxCAR)	0.0002	0.0002	0.0002	0.0002	0.0002	0.0002	0.0001	0.0001	0.0001
BLESS (MCAR)	0.0002	0.0002	0.0002	0.0002	0.0002	0.0002	0.0001	0.0001	0.0001

Table 11: Evaluation of parameter estimates from the methods, BLESS, BSGLMM and Firth Regression via bias, variance and MSE of the spatially-varying coefficients $\hat{\beta}_0$ and $\hat{\beta}_1$.

**Parameter Estimate:** $\hat{\boldsymbol{\beta}}_2$	**Bias**			**Variance**			**MSE**		
**N=500**	$\lambda = 1$	$\lambda = 2$	$\lambda = 3$	$\lambda = 1$	$\lambda = 2$	$\lambda = 3$	$\lambda = 1$	$\lambda = 2$	$\lambda = 3$
BLESS (CAR)	0.0451	0.0252	0.0028	0.2336	0.1345	0.0079	0.2358	0.1271	0.0096
BLESS (PxCAR)	0.0056	0.0029	0.0020	0.0375	0.0112	0.0023	0.0376	0.0097	0.0021
BLESS (MCAR)	0.0026	0.0020	0.0020	0.0106	0.0024	0.0020	0.0282	0.0043	0.0018
**N=1,000**	$\lambda = 1$	$\lambda = 2$	$\lambda = 3$	$\lambda = 1$	$\lambda = 2$	$\lambda = 3$	$\lambda = 1$	$\lambda = 2$	$\lambda = 3$
BLESS (CAR)	0.0084	0.0010	0.0010	0.0420	0.0010	0.0010	0.0504	0.0005	0.0005
BLESS (PxCAR)	0.0015	0.0011	0.0010	0.0049	0.0010	0.0010	0.0082	0.0005	0.0005
BLESS (MCAR)	0.0010	0.0011	0.0010	0.0010	0.0011	0.0010	0.0019	0.0005	0.0005
**N=5,000**	$\lambda = 1$	$\lambda = 2$	$\lambda = 3$	$\lambda = 1$	$\lambda = 2$	$\lambda = 3$	$\lambda = 1$	$\lambda = 2$	$\lambda = 3$
BLESS (CAR)	0.0002	0.0002	0.0002	0.0002	0.0002	0.0002	0.0001	0.0001	0.0001
BLESS (PxCAR)	0.0002	0.0002	0.0002	0.0002	0.0002	0.0002	0.0001	0.0001	0.0001
BLESS (MCAR)	0.0002	0.0002	0.0002	0.0002	0.0002	0.0002	0.0001	0.0001	0.0001

**Predictive Performance:** $\hat{\boldsymbol{y}}$	**Bias**			**Variance**			**MSE**		
**N=500**	$\lambda = 1$	$\lambda = 2$	$\lambda = 3$	$\lambda = 1$	$\lambda = 2$	$\lambda = 3$	$\lambda = 1$	$\lambda = 2$	$\lambda = 3$
BLESS (CAR)	-0.0229	-0.0296	-0.0103	0.0010	0.0034	0.0032	0.0037	0.0090	0.0062
BLESS (PxCAR)	0.0297	0.0239	0.0092	0.0010	0.0017	0.0014	0.0012	0.0019	0.0014
BLESS (MCAR)	-0.0078	-0.0052	-0.0032	0.0011	0.0017	0.0018	0.0022	0.0031	0.0034
**N=1,000**	$\lambda = 1$	$\lambda = 2$	$\lambda = 3$	$\lambda = 1$	$\lambda = 2$	$\lambda = 3$	$\lambda = 1$	$\lambda = 2$	$\lambda = 3$
BLESS (CAR)	-0.0103	0.0003	-0.0014	0.0008	0.0006	0.0007	0.0019	0.0011	0.0012
BLESS (PxCAR)	0.0110	0.0006	0.0002	0.0004	0.0003	0.0004	0.0005	0.0003	0.0004
BLESS (MCAR)	-0.0015	0.0007	-0.0013	0.0004	0.0005	0.0007	0.0008	0.0010	0.0012
**N=5,000**	$\lambda = 1$	$\lambda = 2$	$\lambda = 3$	$\lambda = 1$	$\lambda = 2$	$\lambda = 3$	$\lambda = 1$	$\lambda = 2$	$\lambda = 3$
BLESS (CAR) -0.0007	-0.0000	0.0007	0.0001	0.0001	0.0001	0.0001	0.0002	0.0002	
BLESS (PxCAR)	-0.0001	-0.0004	0.0003	0.0000	0.0000	0.0001	0.0000	0.0000	0.0001
BLESS (MCAR)	-0.0006	-0.0000	0.0008	0.0001	0.0001	0.0001	0.0001	0.0002	0.0003

Table 12: Evaluation of parameter estimates $\hat{\beta}_2$ and the predictive performance $\hat{y}$ from the methods, BLESS, BSGLMM and Firth Regression via bias, variance and MSE.

	TPR			TDR		
**N=500**	$\lambda = 1$	$\lambda = 2$	$\lambda = 3$	$\lambda = 1$	$\lambda = 2$	$\lambda = 3$
BLESS (CAR)	0.1193	0.3630	0.8372	0.9867	0.9997	0.9993
BLESS (PxCAR)	0.5060	0.7654	0.9395	0.9714	0.9953	0.9987
BLESS (MCAR)	0.6635	0.9080	0.9770	0.9520	0.9808	0.9893
**N=1,000**	$\lambda = 1$	$\lambda = 2$	$\lambda = 3$	$\lambda = 1$	$\lambda = 2$	$\lambda = 3$
BLESS (CAR)	0.5667	0.9850	0.9994	0.9987	0.9997	0.9997
BLESS (PxCAR)	0.8111	0.9923	0.9997	0.9970	0.9997	0.9998
BLESS (MCAR)	0.9458	0.9983	1.0000	0.9713	0.9882	0.9936
**N=5,000**	$\lambda = 1$	$\lambda = 2$	$\lambda = 3$	$\lambda = 1$	$\lambda = 2$	$\lambda = 3$
BLESS (CAR)	1.0000	1.0000	1.0000	0.9997	0.9999	0.9999
BLESS (PxCAR)	1.0000	1.0000	1.0000	0.9996	0.9999	0.9999
BLESS (MCAR)	1.0000	1.0000	1.0000	0.9873	0.9973	0.9968
	**FPR**			**FDR**		
**N=500**	$\lambda = 1$	$\lambda = 2$	$\lambda = 3$	$\lambda = 1$	$\lambda = 2$	$\lambda = 3$
BLESS (CAR)	0.0014	0.0001	0.0006	0.0133	0.0003	0.0007
BLESS (PxCAR)	0.0150	0.0036	0.0012	0.0286	0.0047	0.0013
BLESS (MCAR)	0.0334	0.0178	0.0106	0.0480	0.0192	0.0107
**N=1,000**	$\lambda = 1$	$\lambda = 2$	$\lambda = 3$	$\lambda = 1$	$\lambda = 2$	$\lambda = 3$
BLESS (CAR)	0.0010	0.0003	0.0003	0.0013	0.0003	0.0003
BLESS (PxCAR)	0.0024	0.0003	0.0002	0.0030	0.0003	0.0002
BLESS (MCAR)	0.0282	0.0120	0.0065	0.0287	0.0118	0.0064
**N=5,000**	$\lambda = 1$	$\lambda = 2$	$\lambda = 3$	$\lambda = 1$	$\lambda = 2$	$\lambda = 3$
BLESS (CAR)	0.0003	0.0001	0.0001	0.0003	0.0001	0.0001
BLESS (PxCAR)	0.0004	0.0001	0.0001	0.0004	0.0001	0.0001
BLESS (MCAR)	0.0129	0.0027	0.0032	0.0127	0.0027	0.0032

Table 13: Evaluation of inference results from the methods, BLESS, BSGLMM and Firth Regression via TPR, TDR, FPR and FDR for parameter estiamte $\hat{\beta}_1$.

	TPR			TDR		
**N=500**	$\lambda = 1$	$\lambda = 2$	$\lambda = 3$	$\lambda = 1$	$\lambda = 2$	$\lambda = 3$
BLESS (CAR)	0.1192	0.3757	0.8221	0.9718	0.9995	0.9983
BLESS (PxCAR)	0.5064	0.7681	0.9227	0.9352	0.9893	0.9974
BLESS (MCAR)	0.5448	0.8336	0.9344	0.9288	0.9805	0.9930
**N=1,000**	$\lambda = 1$	$\lambda = 2$	$\lambda = 3$	$\lambda = 1$	$\lambda = 2$	$\lambda = 3$
BLESS (CAR)	0.5446	0.9845	0.9995	0.9972	0.9989	0.9994
BLESS (PxCAR)	0.7805	0.9912	0.9998	0.9946	0.9991	0.9995
BLESS (MCAR)	0.8888	0.9942	0.9998	0.9683	0.9909	0.9957
**N=5,000**	$\lambda = 1$	$\lambda = 2$	$\lambda = 3$	$\lambda = 1$	$\lambda = 2$	$\lambda = 3$
BLESS (CAR)	1.0000	1.0000	1.0000	0.9994	0.9997	0.9999
BLESS (PxCAR)	1.0000	1.0000	1.0000	0.9994	0.9997	0.9999
BLESS (MCAR)	1.0000	1.0000	1.0000	0.9851	0.9970	0.9984
	**FPR**			**FDR**		
**N=500**	$\lambda = 1$	$\lambda = 2$	$\lambda = 3$	$\lambda = 1$	$\lambda = 2$	$\lambda = 3$
BLESS (CAR)	0.0011	0.0001	0.0005	0.0282	0.0005	0.0017
BLESS (PxCAR)	0.0117	0.0028	0.0008	0.0648	0.0107	0.0026
BLESS (MCAR)	0.0139	0.0056	0.0022	0.0712	0.0195	0.0070
**N=1,000**	$\lambda = 1$	$\lambda = 2$	$\lambda = 3$	$\lambda = 1$	$\lambda = 2$	$\lambda = 3$
BLESS (CAR)	0.0006	0.0003	0.0002	0.0028	0.0011	0.0006
BLESS (PxCAR)	0.0014	0.0003	0.0002	0.0054	0.0009	0.0005
BLESS (MCAR)	0.0098	0.0031	0.0014	0.0317	0.0091	0.0043
**N=5,000**	$\lambda = 1$	$\lambda = 2$	$\lambda = 3$	$\lambda = 1$	$\lambda = 2$	$\lambda = 3$
BLESS (CAR)	0.0002	0.0001	0.0000	0.0006	0.0003	0.0001
BLESS (PxCAR)	0.0002	0.0001	0.0000	0.0006	0.0003	0.0001
BLESS (MCAR)	0.0051	0.0010	0.0005	0.0149	0.0030	0.0016

Table 14: Evaluation of inference results from the methods, BLESS, BSGLMM and Firth Regression via TPR, TDR, FPR and FDR for parameter estiamte $\hat{\beta}_2$.

9 Simulation Study: Varying Slab Variances

	$\boldsymbol{\lambda = 1}$			$\boldsymbol{\lambda = 2}$			$\boldsymbol{\lambda = 3}$		
**N=500**	β_0	β_1	β_2	β_0	β_1	β_2	β_0	β_1	β_2
Truth	-2.0868	0.5882	0.5586	-1.7933	0.6559	0.6237	-1.6089	0.7051	0.6685
BLESS ($\nu_1 = 1$)	-2.0737	0.5197	0.4479	-1.7905	0.6287	0.5780	-1.6036	0.6852	0.6340
BLESS ($\nu_1 = 10$)	-2.0564	0.4799	0.3941	-1.7836	0.6202	0.5482	-1.6036	0.6910	0.6231
BLESS ($\nu_1 = 100$)	-1.9736	0.3059	0.2429	-1.7480	0.5535	0.4576	-1.5882	0.6662	0.5755
**N=1,000**	β_0	β_1	β_2	β_0	β_1	β_2	β_0	β_1	β_2
Truth	-2.0868	0.5882	0.5586	-1.7933	0.6559	0.6237	-1.6089	0.7051	0.6685
BLESS ($\nu_1 = 1$)	-2.0894	0.5752	0.5271	-1.7932	0.6451	0.6099	-1.6067	0.6920	0.6548
BLESS ($\nu_1 = 10$)	-2.0852	0.5725	0.5088	-1.7952	0.6520	0.6113	-1.6083	0.6982	0.6573
BLESS ($\nu_1 = 100$)	-2.0859	0.5799	0.5501	-1.7911	0.6470	0.5993	-1.6061	0.6963	0.6511
**N=5,000**	β_0	β_1	β_2	β_0	β_1	β_2	β_0	β_1	β_2
Truth	-2.0868	0.5882	0.5586	-1.7933	0.6559	0.6237	-1.6089	0.7051	0.6685
BLESS ($\nu_1 = 1$)	-2.0866	0.5806	0.5539	-1.7930	0.6504	0.6197	-1.6057	0.6972	0.6651
BLESS ($\nu_1 = 10$)	-2.0874	0.5826	0.5555	-1.7933	0.6516	0.6200	-1.6060	0.6982	0.6653
BLESS ($\nu_1 = 100$)	-2.0870	0.5822	0.5546	-1.7929	0.6512	0.6191	-1.6056	0.6978	0.6639

Table 15: Comparison of parameter estimates from the methods, BLESS with three different types of spatial prior, specifically three formulations of a conditional autoregressive prior, BSGLMM and Firth Regression with true coefficient values.

**Parameter Estimate: $\hat{\boldsymbol{\beta}}_0$**	**Bias**			**Variance**			**MSE**		
**N=500**	$\lambda = 1$	$\lambda = 2$	$\lambda = 3$	$\lambda = 1$	$\lambda = 2$	$\lambda = 3$	$\lambda = 1$	$\lambda = 2$	$\lambda = 3$
BLESS ($\nu_1 = 1$)	0.0077	-0.0030	-0.0014	-0.0552	-0.0174	-0.0104	-0.1074	-0.0324	-0.0236
BLESS ($\nu_1 = 10$)	0.0242	0.0014	-0.0043	-0.0961	-0.0237	-0.0009	-0.1661	-0.0584	-0.0284
BLESS ($\nu_1 = 100$)	0.1111	0.0371	0.0095	-0.2824	-0.0932	-0.0247	-0.3391	-0.1561	-0.0758
**N=1,000**	$\lambda = 1$	$\lambda = 2$	$\lambda = 3$	$\lambda = 1$	$\lambda = 2$	$\lambda = 3$	$\lambda = 1$	$\lambda = 2$	$\lambda = 3$
BLESS ($\nu_1 = 1$)	-0.0070	-0.0047	-0.0022	-0.0029	-0.0014	-0.0057	-0.0229	-0.0025	-0.0068
BLESS ($\nu_1 = 10$)	-0.0054	-0.0090	-0.0059	-0.0031	0.0082	0.0019	-0.0373	0.0058	0.0013
BLESS ($\nu_1 = 100$)	0.0088	-0.0072	-0.0059	-0.0287	0.0057	0.0022	-0.0783	-0.0012	0.0011
**N=5,000**	$\lambda = 1$	$\lambda = 2$	$\lambda = 3$	$\lambda = 1$	$\lambda = 2$	$\lambda = 3$	$\lambda = 1$	$\lambda = 2$	$\lambda = 3$
BLESS ($\nu_1 = 1$)	-0.0024	-0.0020	0.0004	0.0006	0.0020	-0.0028	-0.0030	-0.0007	-0.0019
BLESS ($\nu_1 = 10$)	-0.0038	-0.0030	-0.0004	0.0032	0.0039	-0.0011	-0.0002	0.0014	-0.0002
BLESS ($\nu_1 = 100$)	-0.0039	-0.0031	-0.0005	0.0034	0.0040	-0.0009	0.0001	0.0016	-0.0000

**Parameter Estimate: $\hat{\boldsymbol{\beta}}_1$**	**Bias**			**Variance**			**MSE**		
**N=500**	$\lambda = 1$	$\lambda = 2$	$\lambda = 3$	$\lambda = 1$	$\lambda = 2$	$\lambda = 3$	$\lambda = 1$	$\lambda = 2$	$\lambda = 3$
BLESS ($\nu_1 = 1$)	0.0020	0.0020	0.0020	0.0018	0.0021	0.0021	0.0008	0.0010	0.0010
BLESS ($\nu_1 = 10$)	0.0020	0.0020	0.0020	0.0014	0.0019	0.0020	0.0006	0.0009	0.0009
BLESS ($\nu_1 = 100$)	0.0020	0.0020	0.0020	0.0007	0.0016	0.0019	0.0003	0.0007	0.0008
**N=1,000**	$\lambda = 1$	$\lambda = 2$	$\lambda = 3$	$\lambda = 1$	$\lambda = 2$	$\lambda = 3$	$\lambda = 1$	$\lambda = 2$	$\lambda = 3$
BLESS ($\nu_1 = 1$)	0.0010	0.0010	0.0010	0.0011	0.0011	0.0010	0.0005	0.0005	0.0005
BLESS ($\nu_1 = 10$)	0.0010	0.0010	0.0010	0.0010	0.0010	0.0010	0.0005	0.0005	0.0005
BLESS ($\nu_1 = 100$)	0.0010	0.0010	0.0010	0.0009	0.0010	0.0010	0.0004	0.0005	0.0005
**N=5,000**	$\lambda = 1$	$\lambda = 2$	$\lambda = 3$	$\lambda = 1$	$\lambda = 2$	$\lambda = 3$	$\lambda = 1$	$\lambda = 2$	$\lambda = 3$
BLESS ($\nu_1 = 1$)	0.0002	0.0002	0.0002	0.0002	0.0002	0.0002	0.0001	0.0001	0.0001
BLESS ($\nu_1 = 10$)	0.0002	0.0002	0.0002	0.0002	0.0002	0.0002	0.0001	0.0001	0.0001
BLESS ($\nu_1 = 100$)	0.0002	0.0002	0.0002	0.0002	0.0002	0.0002	0.0001	0.0001	0.0001

Table 16: Evaluation of parameter estimates from the methods, BLESS, BSGLMM and Firth Regression via bias, variance and MSE of the spatially-varying coefficients $\hat{\beta}_0$ and $\hat{\beta}_1$.

**Parameter Estimate: $\hat{\beta}_2$**	Bias			Variance			MSE		
**N=500**	$\lambda = 1$	$\lambda = 2$	$\lambda = 3$	$\lambda = 1$	$\lambda = 2$	$\lambda = 3$	$\lambda = 1$	$\lambda = 2$	$\lambda = 3$
BLESS ($\nu_1 = 1$)	0.0021	0.0020	0.0020	0.0048	0.0024	0.0022	0.0124	0.0020	0.0016
BLESS ($\nu_1 = 10$)	0.0026	0.0020	0.0020	0.0106	0.0024	0.0020	0.0282	0.0043	0.0018
BLESS ($\nu_1 = 100$)	0.0144	0.0034	0.0021	0.0805	0.0102	0.0025	0.1153	0.0250	0.0066
**N=1,000**	$\lambda = 1$	$\lambda = 2$	$\lambda = 3$	$\lambda = 1$	$\lambda = 2$	$\lambda = 3$	$\lambda = 1$	$\lambda = 2$	$\lambda = 3$
BLESS ($\nu_1 = 1$)	0.0010	0.0010	0.0010	0.0011	0.0011	0.0011	0.0011	0.0005	0.0006
BLESS ($\nu_1 = 10$)	0.0010	0.0011	0.0010	0.0010	0.0011	0.0010	0.0019	0.0005	0.0005
BLESS ($\nu_1 = 100$)	0.0011	0.0011	0.0010	0.0017	0.0010	0.0010	0.0065	0.0005	0.0005
**N=5,000**	$\lambda = 1$	$\lambda = 2$	$\lambda = 3$	$\lambda = 1$	$\lambda = 2$	$\lambda = 3$	$\lambda = 1$	$\lambda = 2$	$\lambda = 3$
BLESS ($\nu_1 = 1$)	0.0002	0.0002	0.0002	0.0002	0.0002	0.0002	0.0001	0.0001	0.0001
BLESS ($\nu_1 = 10$)	0.0002	0.0002	0.0002	0.0002	0.0002	0.0002	0.0001	0.0001	0.0001
BLESS ($\nu_1 = 100$)	0.0002	0.0002	0.0002	0.0002	0.0002	0.0002	0.0001	0.0001	0.0001

**Predictive Performance: $\hat{y}$**	Bias			Variance			MSE		
**N=500**	$\lambda = 1$	$\lambda = 2$	$\lambda = 3$	$\lambda = 1$	$\lambda = 2$	$\lambda = 3$	$\lambda = 1$	$\lambda = 2$	$\lambda = 3$
BLESS ($\nu_1 = 1$)	-0.0054	-0.0042	-0.0039	0.0009	0.0012	0.0015	0.0017	0.0024	0.0029
BLESS ($\nu_1 = 10$)	0.0206	0.0107	0.0053	0.0008	0.0011	0.0011	0.0009	0.0012	0.0011
BLESS ($\nu_1 = 100$)	-0.0160	-0.0117	-0.0069	0.0012	0.0024	0.0025	0.0030	0.0048	0.0048
**N=1,000**	$\lambda = 1$	$\lambda = 2$	$\lambda = 3$	$\lambda = 1$	$\lambda = 2$	$\lambda = 3$	$\lambda = 1$	$\lambda = 2$	$\lambda = 3$
BLESS ($\nu_1 = 1$)	-0.0014	-0.0002	-0.0024	0.0003	0.0005	0.0007	0.0006	0.0010	0.0013
BLESS ($\nu_1 = 10$)	0.0037	0.0002	0.0001	0.0002	0.0003	0.0004	0.0002	0.0003	0.0004
BLESS ($\nu_1 = 100$)	-0.0031	0.0003	-0.0013	0.0005	0.0006	0.0007	0.0010	0.0011	0.0012
**N=5,000**	$\lambda = 1$	$\lambda = 2$	$\lambda = 3$	$\lambda = 1$	$\lambda = 2$	$\lambda = 3$	$\lambda = 1$	$\lambda = 2$	$\lambda = 3$
BLESS ($\nu_1 = 1$)	-0.0008	-0.0002	0.0005	0.0001	0.0001	0.0001	0.0001	0.0002	0.0003
BLESS ($\nu_1 = 10$)	-0.0001	-0.0004	0.0003	0.0000	0.0000	0.0001	0.0000	0.0000	0.0001
BLESS ($\nu_1 = 100$)	-0.0006	-0.0000	0.0008	0.0001	0.0001	0.0001	0.0001	0.0002	0.0003

Table 17: Evaluation of parameter estimates $\hat{\beta}_2$ and the predictive performance $\hat{y}$ from the methods, BLESS, BSGLMM and Firth Regression via bias, variance and MSE.

	TPR			TDR		
**N=500**	$\lambda = 1$	$\lambda = 2$	$\lambda = 3$	$\lambda = 1$	$\lambda = 2$	$\lambda = 3$
BLESS ($\nu_1 = 1$)	0.8149	0.9709	0.9957	0.9181	0.9475	0.9615
BLESS ($\nu_1 = 10$)	0.6635	0.9080	0.9770	0.9520	0.9808	0.9893
BLESS ($\nu_1 = 100$)	0.3584	0.7728	0.9259	0.9750	0.9931	0.9969
**N=1,000**	$\lambda = 1$	$\lambda = 2$	$\lambda = 3$	$\lambda = 1$	$\lambda = 2$	$\lambda = 3$
BLESS ($\nu_1 = 1$)	0.9866	0.9998	1.0000	0.9228	0.9548	0.9676
BLESS ($\nu_1 = 10$)	0.9458	0.9983	1.0000	0.9713	0.9882	0.9936
BLESS ($\nu_1 = 100$)	0.8815	0.9893	0.9994	0.9861	0.9971	0.9991
**N=5,000**	$\lambda = 1$	$\lambda = 2$	$\lambda = 3$	$\lambda = 1$	$\lambda = 2$	$\lambda = 3$
BLESS ($\nu_1 = 1$)	1.0000	1.0000	1.0000	0.9744	0.9823	0.9852
BLESS ($\nu_1 = 10$)	1.0000	1.0000	1.0000	0.9873	0.9973	0.9968
BLESS ($\nu_1 = 100$)	1.0000	1.0000	1.0000	0.9936	0.9993	0.9996
	**FPR**			**FDR**		
**N=500**	$\lambda = 1$	$\lambda = 2$	$\lambda = 3$	$\lambda = 1$	$\lambda = 2$	$\lambda = 3$
BLESS ($\nu_1 = 1$)	0.0729	0.0541	0.0401	0.0819	0.0525	0.0385
BLESS ($\nu_1 = 10$)	0.0334	0.0178	0.0106	0.0480	0.0192	0.0107
BLESS ($\nu_1 = 100$)	0.0090	0.0054	0.0029	0.0250	0.0069	0.0031
**N=1,000**	$\lambda = 1$	$\lambda = 2$	$\lambda = 3$	$\lambda = 1$	$\lambda = 2$	$\lambda = 3$
BLESS ($\nu_1 = 1$)	0.0831	0.0476	0.0337	0.0772	0.0452	0.0324
BLESS ($\nu_1 = 10$)	0.0282	0.0120	0.0065	0.0287	0.0118	0.0064
BLESS ($\nu_1 = 100$)	0.0125	0.0029	0.0009	0.0139	0.0029	0.0009
**N=5,000**	$\lambda = 1$	$\lambda = 2$	$\lambda = 3$	$\lambda = 1$	$\lambda = 2$	$\lambda = 3$
BLESS ($\nu_1 = 1$)	0.0264	0.0181	0.0151	0.0256	0.0177	0.0148
BLESS ($\nu_1 = 10$)	0.0129	0.0027	0.0032	0.0127	0.0027	0.0032
BLESS ($\nu_1 = 100$)	0.0065	0.0007	0.0004	0.0064	0.0007	0.0004

Table 18: Evaluation of inference results from the methods, BLESS, BSGLMM and Firth Regression via TPR, TDR, FPR and FDR for parameter estiamte $\hat{\beta}_1$.

	**TPR**			**TDR**		
**N=500**	$\lambda = 1$	$\lambda = 2$	$\lambda = 3$	$\lambda = 1$	$\lambda = 2$	$\lambda = 3$
BLESS ($\nu_1 = 1$)	0.7094	0.9280	0.9798	0.8852	0.9518	0.9741
BLESS ($\nu_1 = 10$)	0.5448	0.8336	0.9344	0.9288	0.9805	0.9930
BLESS ($\nu_1 = 100$)	0.2839	0.6634	0.8451	0.9528	0.9925	0.9978
**N=1,000**	$\lambda = 1$	$\lambda = 2$	$\lambda = 3$	$\lambda = 1$	$\lambda = 2$	$\lambda = 3$
BLESS ($\nu_1 = 1$)	0.9608	0.9990	1.0000	0.9175	0.9589	0.9757
BLESS ($\nu_1 = 10$)	0.8888	0.9942	0.9998	0.9683	0.9909	0.9957
BLESS ($\nu_1 = 100$)	0.7980	0.9759	0.9976	0.9847	0.9974	0.9993
**N=5,000**	$\lambda = 1$	$\lambda = 2$	$\lambda = 3$	$\lambda = 1$	$\lambda = 2$	$\lambda = 3$
BLESS ($\nu_1 = 1$)	1.0000	1.0000	1.0000	0.9702	0.9844	0.9913
BLESS ($\nu_1 = 10$)	1.0000	1.0000	1.0000	0.9851	0.9970	0.9984
BLESS ($\nu_1 = 100$)	1.0000	1.0000	1.0000	0.9920	0.9987	0.9998
	**FPR**			**FDR**		
**N=500**	$\lambda = 1$	$\lambda = 2$	$\lambda = 3$	$\lambda = 1$	$\lambda = 2$	$\lambda = 3$
BLESS ($\nu_1 = 1$)	0.0309	0.0158	0.0087	0.1148	0.0482	0.0259
BLESS ($\nu_1 = 10$)	0.0139	0.0056	0.0022	0.0712	0.0195	0.0070
BLESS ($\nu_1 = 100$)	0.0045	0.0017	0.0006	0.0472	0.0075	0.0022
**N=1,000**	$\lambda = 1$	$\lambda = 2$	$\lambda = 3$	$\lambda = 1$	$\lambda = 2$	$\lambda = 3$
BLESS ($\nu_1 = 1$)	0.0290	0.0144	0.0084	0.0825	0.0411	0.0243
BLESS ($\nu_1 = 10$)	0.0098	0.0031	0.0014	0.0317	0.0091	0.0043
BLESS ($\nu_1 = 100$)	0.0042	0.0009	0.0002	0.0153	0.0026	0.0007
**N=5,000**	$\lambda = 1$	$\lambda = 2$	$\lambda = 3$	$\lambda = 1$	$\lambda = 2$	$\lambda = 3$
BLESS ($\nu_1 = 1$)	0.0103	0.0053	0.0030	0.0298	0.0156	0.0087
BLESS ($\nu_1 = 10$)	0.0051	0.0010	0.0005	0.0149	0.0030	0.0016
BLESS ($\nu_1 = 100$)	0.0027	0.0004	0.0001	0.0080	0.0013	0.0002

Table 19: Evaluation of inference results from the methods, BLESS, BSGLMM and Firth Regression via TPR, TDR, FPR and FDR for parameter estiamte $\hat{\beta}_2$.

10 Simulation Study: Sensitivity Analysis of Neighborhood Structure

In order to ensure the reproducibility of our method and to strengthen the robustness of BLESS we perform an additional sensitivity analysis on the size of the neighborhood structure of the MCAR prior. To illustrate the difference in neighborhood structures we provide the below figure where the left depicts the previous neighborhood structure where the sum of the neighbors of $\theta(s_5)$ on a 2D lattice is calculated by the blue colored set of four neighbors $\partial\theta(s_5) = \{\theta(s_2), \theta(s_4), \theta(s_6), \theta(s_8)\}$. In the sensitivity analysis, we now extend the neighborhood structure to a set of eight neighbors where the set is given by $\partial\theta(s_5) = \{\theta(s_1), \theta(s_2), \theta(s_3), \theta(s_4), \theta(s_6), \theta(s_7), \theta(s_8), \theta(s_9)\}$.

$\theta(s_1)$	$\boldsymbol{\theta(s_2)}$	$\theta(s_3)$
$\boldsymbol{\theta(s_4)}$	$\theta(s_5)$	$\boldsymbol{\theta(s_6)}$
$\theta(s_7)$	$\boldsymbol{\theta(s_8)}$	$\theta(s_9)$

$\boldsymbol{\theta(s_1)}$	$\boldsymbol{\theta(s_2)}$	$\boldsymbol{\theta(s_3)}$
$\boldsymbol{\theta(s_4)}$	$\theta(s_5)$	$\boldsymbol{\theta(s_6)}$
$\boldsymbol{\theta(s_7)}$	$\boldsymbol{\theta(s_8)}$	$\boldsymbol{\theta(s_9)}$

We show that BB-BLESS is robust to changing the tuning parameter which influences the spatial dependence structure of our method. Both the performance of parameter estimates and predictive performance as well as inference results yield very similar results for the extended neighborhood structure, consisting of 8 neighbors, versus the original choice of tuning parameter of 4 neighbors, see Table 20 and 21.

Parameter Estimate: $\hat{\beta}_1$	Bias			Variance			MSE		
**N=500**	$\lambda = 1$	$\lambda = 2$	$\lambda = 3$	$\lambda = 1$	$\lambda = 2$	$\lambda = 3$	$\lambda = 1$	$\lambda = 2$	$\lambda = 3$
BLESS (4 neighbours)	-0.0961	-0.0237	-0.0009	0.0014	0.0019	0.0020	0.0106	0.0024	0.0020
BLESS (8 neighbours)	-0.0945	-0.0205	-0.0001	0.0014	0.0019	0.0020	0.0103	0.0023	0.0020
**N=1,000**	$\lambda = 1$	$\lambda = 2$	$\lambda = 3$	$\lambda = 1$	$\lambda = 2$	$\lambda = 3$	$\lambda = 1$	$\lambda = 2$	$\lambda = 3$
BLESS (4 neighbours)	-0.0031	0.0082	0.0019	0.0010	0.0010	0.0010	0.0010	0.0011	0.0010
BLESS (8 neighbours)	0.0104	-0.0047	0.0088	0.0010	0.0010	0.0010	0.0011	0.0010	0.0011
**N=5,000**	$\lambda = 1$	$\lambda = 2$	$\lambda = 3$	$\lambda = 1$	$\lambda = 2$	$\lambda = 3$	$\lambda = 1$	$\lambda = 2$	$\lambda = 3$
BLESS (4 neighbours)	0.0032	0.0039	-0.0011	0.0002	0.0002	0.0002	0.0002	0.0002	0.0002
BLESS (8 neighbours)	0.0032	0.0039	-0.0011	0.0002	0.0002	0.0002	0.0002	0.0002	0.0002

Predictive Performance: $\hat{\boldsymbol{y}}$	Bias			Variance			MSE		
**N=500**	$\lambda = 1$	$\lambda = 2$	$\lambda = 3$	$\lambda = 1$	$\lambda = 2$	$\lambda = 3$	$\lambda = 1$	$\lambda = 2$	$\lambda = 3$
BLESS (4 neighbours)	-0.0078	-0.0052	-0.0032	0.0011	0.0017	0.0018	0.0022	0.0031	0.0034
BLESS (8 neighbours)	-0.0077	-0.0049	-0.0031	0.0011	0.0016	0.0018	0.0022	0.0031	0.0033
**N=1,000**	$\lambda = 1$	$\lambda = 2$	$\lambda = 3$	$\lambda = 1$	$\lambda = 2$	$\lambda = 3$	$\lambda = 1$	$\lambda = 2$	$\lambda = 3$
BLESS (4 neighbours)	-0.0015	0.0007	-0.0013	0.0004	0.0005	0.0007	0.0008	0.0010	0.0012
BLESS (8 neighbours)	-0.0072	-0.0015	-0.0007	0.0004	0.0005	0.0007	0.0008	0.0009	0.0013
**N=5,000**	$\lambda = 1$	$\lambda = 2$	$\lambda = 3$	$\lambda = 1$	$\lambda = 2$	$\lambda = 3$	$\lambda = 1$	$\lambda = 2$	$\lambda = 3$
BLESS (4 neighbours)	-0.0006	0.0000	0.0008	0.0001	0.0001	0.0001	0.0001	0.0002	0.0003
BLESS (8 neighbours)	-0.0006	0.0000	0.0008	0.0001	0.0001	0.0001	0.0001	0.0002	0.0003

Table 20: Sensitivity analysis evaluating parameter estimates of BLESS with a spatial prior of four and eight neighbours via bias, variance and MSE of the spatially-varying coefficients $\hat{\boldsymbol{\beta}}_{\mathbf{1}}$, and the predictive performance $\hat{\boldsymbol{y}}$. Variations of the neighbourhood in the spatial prior of BLESS leave the results relatively unchanged. Accounting for a larger spatial neighbourhood potentially reduces bias in the parameter estimates and prediction results for lower sample sizes, such as $N = 500$.

	TPR			TDR		
**N=500**	$\lambda = 1$	$\lambda = 2$	$\lambda = 3$	$\lambda = 1$	$\lambda = 2$	$\lambda = 3$
BLESS (4 neighbours)	0.6635	0.9080	0.9770	0.9520	0.9808	0.9893
BLESS (8 neighbours)	0.6679	0.9140	0.9788	0.9512	0.9795	0.9881
**N=1,000**	$\lambda = 1$	$\lambda = 2$	$\lambda = 3$	$\lambda = 1$	$\lambda = 2$	$\lambda = 3$
BLESS (4 neighbours)	0.9458	0.9983	1.0000	0.9713	0.9882	0.9936
BLESS (8 neighbours)	0.9499	0.9989	1.0000	0.9665	0.9768	0.9915
**N=5,000**	$\lambda = 1$	$\lambda = 2$	$\lambda = 3$	$\lambda = 1$	$\lambda = 2$	$\lambda = 3$
BLESS (4 neighbours)	1.0000	1.0000	1.0000	0.9873	0.9973	0.9968
BLESS (8 neighbours)	1.0000	1.0000	1.0000	0.9867	0.9964	0.9960

	FPR			FDR		
**N=500**	$\lambda = 1$	$\lambda = 2$	$\lambda = 3$	$\lambda = 1$	$\lambda = 2$	$\lambda = 3$
BLESS (4 neighbours)	0.0334	0.0178	0.0106	0.0480	0.0192	0.0107
BLESS (8 neighbours)	0.0342	0.0191	0.0119	0.0488	0.0205	0.0119
**N=1,000**	$\lambda = 1$	$\lambda = 2$	$\lambda = 3$	$\lambda = 1$	$\lambda = 2$	$\lambda = 3$
BLESS (4 neighbours)	0.0282	0.0120	0.0065	0.0287	0.0118	0.0064
BLESS (8 neighbours)	0.0331	0.0238	0.0086	0.0335	0.0232	0.0085
**N=5,000**	$\lambda = 1$	$\lambda = 2$	$\lambda = 3$	$\lambda = 1$	$\lambda = 2$	$\lambda = 3$
BLESS (4 neighbours)	0.0129	0.0027	0.0032	0.0127	0.0027	0.0032
BLESS (8 neighbours)	0.0135	0.0037	0.0040	0.0133	0.0036	0.0040

Table 21: Sensitivity analysis evaluating inference results of BLESS with a spatial prior of four and eight neighbours via TPR, TDR, FPR and FDR for parameter estimate $\hat{\beta}_1$. Extending the neighbourhood in the spatial prior of BLESS to eight voxels instead of the original four voxels leaves the inference results fairly unchanged. Sensitivity is slightly increased and specificity is slightly descreased by a larger spatial neighbourhood.

11 Computational Complexity of BLESS-VI

	N=500			N=1,000			N=5,000		
**Model**	$\lambda = 1$	$\lambda = 2$	$\lambda = 3$	$\lambda = 1$	$\lambda = 2$	$\lambda = 3$	$\lambda = 1$	$\lambda = 2$	$\lambda = 3$
BLESS-Gibbs	13:57:00	13:58:00	14:02:00	14:43:00	12:50:00	14:58:00	18:04:00	21:49:00	21:42:00
BB-BLESS	00:02:41	00:02:44	00:02:46	00:04:01	00:03:56	00:03:11	00:09:33	00:09:25	00:10:03
BLESS-VI	00:09:26	00:09:47	00:09:32	00:12:26	00:12:01	00:12:04	00:31:32	00:31:30	00:31:47
BSGLMM	06:44:00	07:44:00	08:23:00	08:26:00	08:35:00	08:14:00	15:14:00	15:49:00	15:04:00
Firth	00:01:16	00:01:11	00:01:08	00:01:36	00:01:29	00:01:25	00:03:47	00:03:33	00:03:19

Table 22: Average computational times (in hours) for all methods, BB-BLESS, BLESS-VI, BLESS-Gibbs, BSGLMM, and Firth Regression, across 100 simulated datasets. The average time for BB-BLESS is given by the average over all datasets as well as the average over all bootstraps considering that the method is parallelisable for each bootstrap.

Quantity	Dimensions	Space Complexity
$q(\boldsymbol{Z})$	$N \times M$	$\mathcal{O}([N \times (P+1) \times M]^2)$
$q(\boldsymbol{\beta})$	$P \times M$	$\mathcal{O}(N \times P^2 \times M)$
$q(\boldsymbol{\beta_0})$	$M \times 1$	$\mathcal{O}(N \times P \times M + M^2)$
$q(\boldsymbol{\gamma})$	$P \times M$	$\mathcal{O}([P \times M]^2)$
$q(\boldsymbol{\theta})$	$P \times M$	$\mathcal{O}(P^2 \times M)$
$q(\boldsymbol{\xi})$	$P \times M$	$\mathcal{O}(P^2 \times M)$
$q(\boldsymbol{\Sigma^{-1}})$	$P \times P$	$\mathcal{O}(P^2)$
ELBO	1×1	$\mathcal{O}([N \times M]^2)$

Table 23: Computational complexity of BLESS-VI algorithm for each variational update and the calculation of the ELBO.

12 Simulation Study with Realistic Lesion Masks

The results of this section are based on a simulation study framework developed by Kindalova et al. (2021). In their work, they simulate artificial lesion maps with realistic lesion patterns by generating a voxel-wise distribution of lesions across age which uses the age effect on lesion probability estimated from a subset of the UK Biobank population. The simulation study framework yields realistic lesion masks by matching brain lesion summaries, such as total lesion volume, average lesion size, and lesion count, across the reference dataset from the UK Biobank and the simulated datasets. We simulate a dataset of 1,000 subjects within the simulation framework provided by Kindalova et al. (2021) on their Github page (https://github.com/petyakindalova/LesionMaskSimulation) for the evaluation of our methods (BLESS-Gibbs, BB-BLESS, and BLESS-VI) compared to other approaches (BSGLMM and Firth Regression). The ground truth spatially varying regression coefficients were obtained from the reference dataset (13,000 subjects) estimated regression coefficients for the age effect where the effect map was thresholded to have both null and real effects. We do this by running the command *'cluster'* in the neuroimaging software *FSL* with a cluster-defining threshold of 0.05.

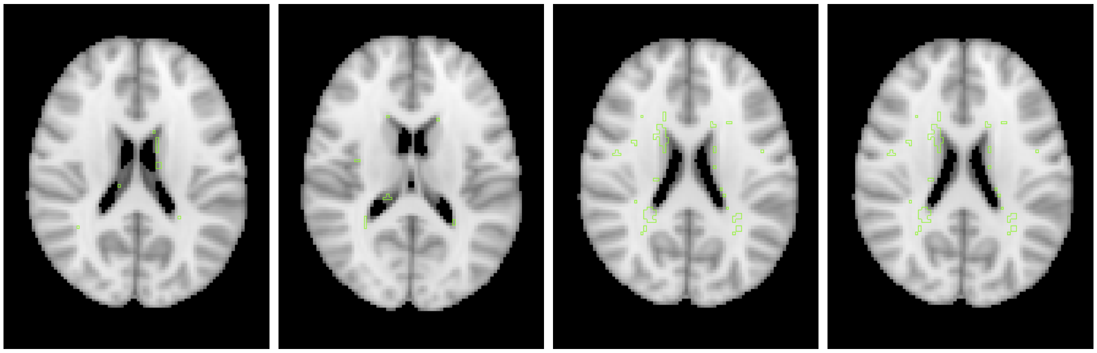

Figure 16: Lesion masks generated via simulation framework based on UK Biobank summary statistics. Two most left-hand lesion masks stem from younger subjects with fewer lesions opposed to the two right-hand lesion masks which were generated for older subjects. Lesion masks are overlayed on the MNI template and lesion borders are depicted by green outlines.

	Parameter Estimate: $\hat{\boldsymbol{\beta}}_1$			Predictive Performance: $\hat{\boldsymbol{y}}$		
	Bias	Variance	MSE	Bias	Variance	MSE
BLESS-Gibbs	0.00073	0.00024	0.00035	-0.00020	0.00544	2.19 $\times 10^{-5}$
BB-BLESS	0.00095	0.00011	0.00046	-0.00023	0.00544	2.25 $\times 10^{-5}$
BLESS-VI	0.00083	0.00005	0.00045	-0.00022	0.00543	2.21 $\times 10^{-5}$
BSGLMM	-0.00227	0.00027	0.00069	-0.00050	0.00544	2.30 $\times 10^{-5}$
Firth	-0.00209	0.00041	0.00050	0.00082	0.00542	6.90 $\times 10^{-7}$

Table 24: Evaluation of parameter estimates for the effect of age from various methods where truth is determined by a simulation framework which utilizes UK Biobank summary statistics by Kindalova et al. (2021).

**Inference Results**	TPR	TDR	FPR	FDR
BLESS-Gibbs	0.9278	1.0000	0.0000	0.0000
BB-BLESS	0.8910	1.0000	0.0000	0.0000
BLESS-VI	0.9049	1.0000	0.0000	0.0000
BSGLMM	0.8990	0.9932	0.0031	0.0068
Firth	0.7393	0.9959	0.0015	0.0041

Table 25: Evaluation of inference results from various methods where truth is determined by a simulation framework which utilizes UK Biobank summary statistics by Kindalova et al. (2021).

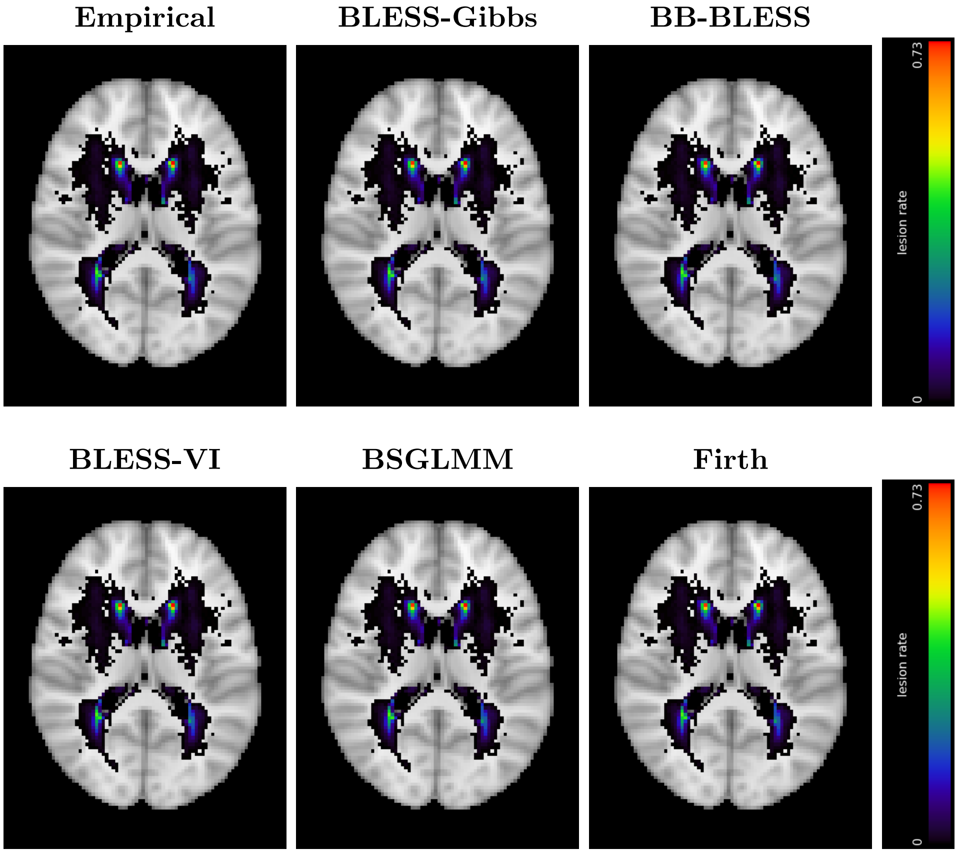

Figure 17: True empirical lesion rate map and predicted lesion rate maps (BLESS-Gibbs, BB-BLESS, BLESS-VI, BSGLMM, and Firth Regression).

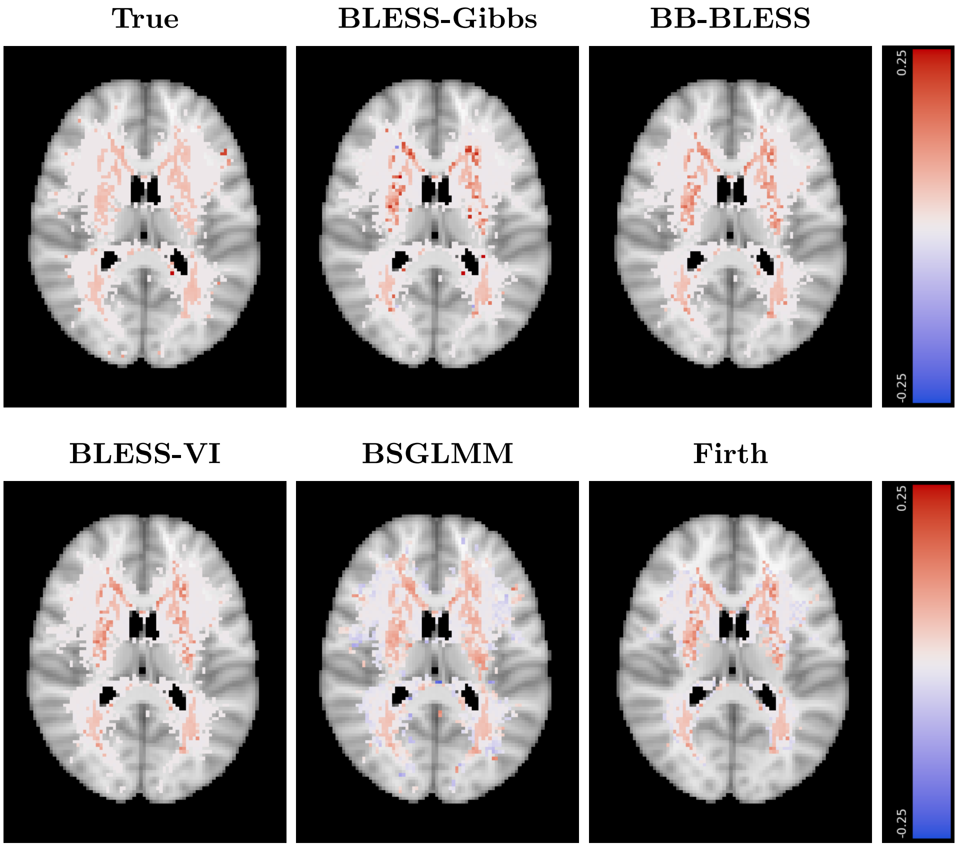

Figure 18: True parameter map and estimated parameter maps for the covariate age via realistic lesion mask simulation framework for the methods, BLESS-Gibbs, BB-BLESS, BLESS-VI, BSGLMM, and Firth Regression.

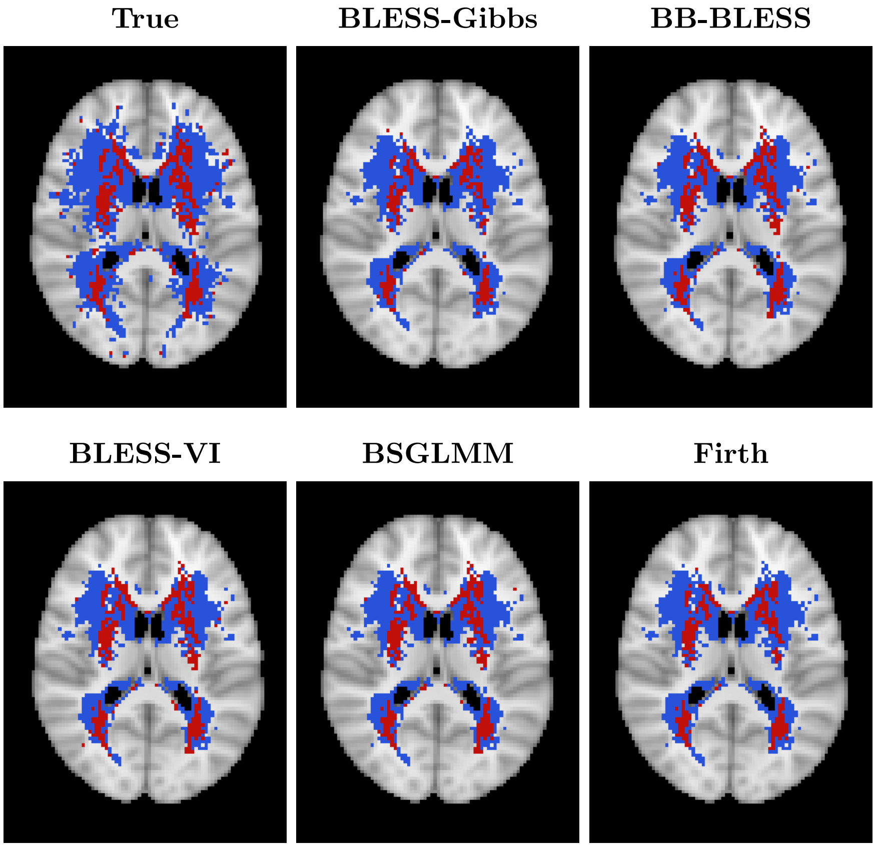

Figure 19: True binary significance map and estimated binary significance maps for the covariate age via realistic lesion mask simulation framework for the methods, BLESS-Gibbs, BB-BLESS, BLESS-VI, BSGLMM, and Firth Regression.

13 Literature Review on Bayesian Spatial Models Designed for Neuroimaging Applications

In the literature, there are various Bayesian spatial approaches for scalar-on-image, image-on-scalar and image-on-image regression models that tackle complex problems within the area of neuroimaging applications. Lesion modeling is less common in the literature; however, most of the below listed methods, implemented on applications, such as structural, task-based, or resting-state functional MRI, could easily be extended to binary lesion mapping problems which were originally derived from structural MRI scans.

For scalar-on-image regression problems, Goldsmith J (2014) have proposed imposing an Ising prior on the indicator variables, which determine variable selection, and an intrinsic Gaussian Markov Random Field prior on the non-zero coefficients in order to induce spatial smoothing. Utilizing an Ising model poses challenges as it suffers from unstable transitions between sparse and non-spare model configurations depending on the selection of model hyperparameters (Li and Zhang, 2010a). Kang et al. (2018) on the other hand propose a Bayesian nonparametric model with piecewise-smooth, sparse and continuous spatially varying coefficient functions with an application to electroencephalogram (EEG) data. The approach performs well in scenarios where the number of parameters is larger than the number of subjects. However, the Metropolis-Hastings steps required to perform inference on some of the parameters within the model is prohibitive to scaling the model to larger sample sizes. Other models discussing Bayesian solutions to scalar-on-image regressions are found in the work by Wang et al. (2017) and Xue et al. (2018).

In our work we propose a model where lesion masks are considered as the output and scalar covariates are provided as the input to our model which is considered an image-on-scalar regression problem. Other Bayesian approaches besides ours have been suggested to model the spatial dependence found in neuroimaging applications more accurately and to introduce sparsity to the model. For example, Zeng et al. (2022) build a Bayesian hierarchical Gaussian Process model that is able to perform global and local variable selection. The model proposed by Li et al. (2021) tackles the spatial heterogeneity of covariate effects on image responses by introducing bivariate spline functions over triangulation. Another example of Bayesian spatial models for image-on-scalar regression problems is given by Whiteman (2022) who combines spatially varying regression coefficient functions via

Gaussian processes and introduces a nonstationary model for the error process to achieve a better data-adaptive smoothing property. The references listed here are certainly non exhaustive; however, for more Bayesian spatial image-on-scalar regression models, see the work by Derado et al. (2013), Chen et al. (2016) and Roy et al. (2021).

Lastly, we give an overview of methods that have been proposed for applications addressing image-on-image regression problems. Guo et al. (2022) suggest a spatial Bayesian latent factor model to address the complex spatial dependence structure between image covariates and image outcomes via Gaussian processes. Moreover, Roy et al. (2021) provide a general framework for using their so-called product independent Gaussian process priors that easily extends to any of the three proposed regression structures (scalar-on-image, image-on-scalar, image-on-image).

14 Extended Introduction on Bayesian Variable Selection and Approximate Posterior Sampling

14.1 Bayesian Variable Selection

We utilize Bayesian variable selection to improve brain lesion mapping by shrinking small coefficients towards zero, thus helping with prediction, interpretation and reduction of spurious associations in high-dimensional settings. A commonly applied technique for Bayesian variable selection is spike-and-slab regression which aims to identify a selection of predictors within a regression model. The original formulation of the spike-and-slab mixture prior places a mixture of a point mass at zero and a diffuse distribution on the coefficients (Mitchell and Beauchamp, 1988). George and McCulloch (1993, 1997) have increased the computational feasibility of spike-and-slab regression problems by introducing a continuous mixture of Gaussian formulation where the spike distribution is defined by a normal distribution with a small variance rather than a point mass prior. The binary latent variable, sampled from a Bernoulli distribution with inclusion probability, determines which mixture component a variable belongs to and enables variable selection. Other versions of spike-and-slab priors include non-local prior densities (Johnson and Rossell, 2010), spike-and-slab LASSO priors (Ročková and George, 2018), and global-local priors, such as the horseshoe prior (Carvalho et al., 2009). Overall, the options of continuous shrinkage priors in the literature are large, see Piironen and Vehtari (2017) for a comparison of different methods. We focus on continuous spike-and-slab priors due to their computationally tractability resulting from the continuity of the spike distribution. Moreover, Bayesian variable selection with traditional spike-and-slab priors relies on MCMC methods whereas shrinkage priors include all covariates in the model and only regularize regression coefficients close to zero.

14.1.1 Spatial Spike-and-Slab Regression

The spike-and-slab regression is also able to incorporate spatial information, replacing the exchangeable Bernoulli prior on the inclusion indicator variables, with a structured spatial prior using a vector of inclusion probabilities. Previous examples of introducing structure within a spike-and-slab regression include the placement of a logistic regression product prior (Stingo et al., 2010) on the latents in order to group biological information for a

genetics application or an Ising prior which incorporates structural information for a high-dimensional genomics application (Li and Zhang, 2010b). Andersen et al. (2014) on the other hand have developed a structured spike-and-slab distribution which models sparsity by placing a spatial Gaussian process on a parameter within the inclusion probabilities. Note that they do not place a structured prior on the probability of inclusion directly but rather on a sparsity parameter and then ensure values between 0 and 1 as inclusion probabilities by transforming the parameter with a suitable injective function. We combine some of these approaches by utilizing the logistic regression prior approach by Stingo et al. (2010) and by placing a spatial prior on the sparsity parameter which influences the probability of inclusion. Specifically, we choose a multivariate conditional autoregressive (MCAR) prior to account for the spatial dependence within the neuroimaging data (Gelfand and Vounatsou, 2003). Furthermore, we avoid an Ising prior on the binary latent indicators which often exhibits the issue of phase transitions. As noted by Ročková and George (2014) and Li and Zhang (2010b), the occurrence of phase transitions, especially when the sparsity parameters exhibit a complex spatial structure, is problematic because they introduce a drastic change in the amount of active voxels due to only a very small change in hyperparameters.

14.2 Approximate Posterior Inference and Sampling

The gold standard of parameter estimation and inference for spike-and-slab regression with a continuous mixture of Gaussians prior is Gibbs sampling (George and McCulloch, 1993). However, in high-dimensional regression settings as well as large sample size scenarios other more scalable approximate methods are required due to the intense computational burden.

Expectation propagation (EP) (Minka, 2001) or variational inference (Jordan et al., 1999) algorithms redefine the problem of approximating densities through optimization (Blei et al., 2017). Both of these methods have been extensively studied for spike-and-slab regression problems (Hernández-Lobato et al., 2013; Carbonetto and Stephens, 2012). The EP algorithm however poses several challenges as it is computationally intensive for even moderate sample sizes, there is no guarantee of convergence, and its poor performance for multimodal posteriors due to the problematic need to incorporate all modes in its approximation (Bishop, 2006). Poor variational approximations can arise due to slow convergence, a simplistic choice of variational families, or due to underestimation of the

posterior variance as the KL-divergence tends to under-penalize thin tails (Yao et al., 2018).

In neuroimaging applications, however, we require accurate uncertainty estimates and hence we use approximate posterior sampling which captures the marginal posterior density more accurately than variational densities while remaining to be highly scalable due to embarrassingly parallel implementations (Fong et al., 2019). The cornerstone of these methods lies in the Bayesian bootstrap (Rubin, 1981) and the Weighted Likelihood Bootstrap (WLB) (Newton and Raftery, 1994). The WLB randomly re-weights the likelihood with Dirichlet weights for the observations and maximizes this likelihood with respect to the parameter of interest. Using WLB, Lyddon et al. (2018) and Fong et al. (2019) developed Bayesian nonparametric learning (BNL) routines which utilize parametric models to achieve posterior sampling through the optimization of randomized objective functions.

Our focus lies on the recently introduced method by Nie and Ročková (2022) which combines Bayesian bootstrap methods with a new class of jittered spike-and-slab LASSO priors and obtains samples via optimization of many independently perturbed datasets by re-weighting the likelihood and by jittering the prior with a random mean shift. This procedure is equivalent to adding pseudo-samples from a prior sampling distribution as in the case of BNL (Fong et al., 2019). We argue that for high-dimensional datasets with large samples, where memory allocation is already a computational concern, the approach by Nie and Ročková (2022) is favorable as it merely requires storing a set of mean shift parameters compared to an arbitrarily large number of pseudo-samples (Fong et al., 2019).

Another advantage of using bootstrapping techniques is that we can acquire posteriors on complex imaging statistics, such as cluster size. In neuroimaging, a cluster is a contiguous set of voxels with voxelwise statistics that exceed some cluster-defining threshold (Poline and Mazoyer, 1993). Cluster-wise inference, is declaring clusters significant when they exceed a given size, has been found to be more sensitive to detect true positive effects than voxelwise inference (Friston et al., 1996), albeit having less spatial specificity as the null hypothesis is specified at the level of clusters. These existing methods can only produce p-values, while we are able to compute credible intervals for cluster size or any other spatial feature of interest by incorporating bootstrapping into our model.

References

Michael Riis Andersen, Ole Winther, and Lars Kai Hansen. Bayesian inference for structured spike and slab priors. *Advances in Neural Information Processing Systems*, 2: 1745–1753, 2014.

Christopher M. Bishop. *Pattern Recognition and Machine Learning*. 2006.

David M. Blei, Alp Kucukelbir, and Jon D. McAuliffe. Variational Inference: A Review for Statisticians. *Journal of the American Statistical Association*, 112(518):859–877, 2017.

Valentina Bordin, Ilaria Bertani, Irene Mattioli, Vaanathi Sundaresan, Paul McCarthy, Sana Suri, Enikő Zsoldos, Nicola Filippini, Abda Mahmood, Luca Melazzini, Maria Marcella Laganà, Giovanna Zamboni, Archana Singh-Manoux, Mika Kivimäki, Klaus P. Ebmeier, Giuseppe Baselli, Mark Jenkinson, Clare E. Mackay, Eugene P. Duff, and Ludovica Griffanti. Integrating large-scale neuroimaging research datasets: Harmonisation of white matter hyperintensity measurements across whitehall and uk biobank datasets. *NeuroImage*, 237:118189, 8 2021.

Peter Carbonetto and Matthew Stephens. Scalable variational inference for bayesian variable selection in regression, and its accuracy in genetic association studies. *Bayesian Analysis*, 7(1):73–108, 2012. ISSN 19360975. doi: 10.1214/12-BA703.

Carlos M. Carvalho, Nicholas G. Polson, and James G. Scott. Handling sparsity via the horseshoe. volume 5 of *PMLR*, pages 73–80, 2009.

Yao Chen, Xiao Wang, Linglong Kong, and Hongtu Zhu. Local region sparse learning for image-on-scalar regression, 2016.

Gordana Derado, F DuBois Bowman, Lijun Zhang, , and the Alzheimer's Disease Neuroimaging Initiative. Predicting brain activity using a bayesian spatial model. *Statistical Methods in Medical Research*, 22(4):382–397, 2013.

Edwin Fong, Simon Lyddon, and Chris Holmes. Scalable nonparametric sampling from multimodal posteriors with the posterior bootstrap. volume 97 of *Proceedings of Machine Learning Research*, pages 1952–1962, 2019.

K. J. Friston, A. Holmes, J. B. Poline, C. J. Price, and C. D. Frith. Detecting activations in pet and fmri: levels of inference and power. *NeuroImage*, 4:223–235, 1996.

Alan E. Gelfand and Penelope Vounatsou. Proper multivariate conditional autoregressive models for spatial data analysis. *Biostatistics*, 4(1):11–15, 2003.

Edward I. George and Robert E. McCulloch. Variable selection via gibbs sampling. *Journal of the American Statistical Association*, 88(423):881–889, 1993.

Edward I. George and Robert E. McCulloch. Approaches for bayesian variable selection. *Statistica Sinica*, 7(2):339–373, 1997.

Crainiceanu CM. Goldsmith J, Huang L. Smooth scalar-on-image regression via spatial bayesian variable selection. *J Comput Graph Stat*, 23(1):46–64, 2014.

Ludovica Griffanti, Giovanna Zamboni, Aamira Khan, Linxin Li, Guendalina Bonifacio, Vaanathi Sundaresan, Ursula G. Schulz, Wilhelm Kuker, Marco Battaglini, Peter M. Rothwell, and Mark Jenkinson. Bianca (brain intensity abnormality classification algorithm): A new tool for automated segmentation of white matter hyperintensities. *NeuroImage*, 141:191–205, 2016.

Cui Guo, Jian Kang, and Timothy D. Johnson. A spatial bayesian latent factor model for image-on-image regression. *Biometrics*, 78(1):72–84, 2022.

Daniel Hernández-Lobato, José Miguel Hernández-Lobato, and Pierre Dupont. Generalized spike-and-slab priors for Bayesian group feature selection using expectation propagation. *Journal of Machine Learning Research*, 14:1891–1945, 2013.

Valen E. Johnson and David Rossell. On the use of non-local prior densities in bayesian hypothesis tests. *Journal of the Royal Statistical Society: Series B (Statistical Methodology)*, 72(2):143–170, 2010.

Michael I. Jordan, Zoubin Ghahramani, Tommi S. Jaakkola, and Lawrence K. Saul. An introduction to variational methods for graphical models. *Mach. Learn.*, 37(2):183–233, 1999.

Jian Kang, Brian J Reich, and Ana-Maria Staicu. Scalar-on-image regression via the soft-thresholded Gaussian process. *Biometrika*, 105(1):165–184, 01 2018.

Petya Kindalova, Ioannis Kosmidis, and Thomas E. Nichols. Voxel-wise and spatial modelling of binary lesion masks: Comparison of methods with a realistic simulation framework. *NeuroImage*, 236:118090, 2021. ISSN 1053-8119. doi: https://doi.org/10.1016/j.neuroimage.2021.118090. URL https://www.sciencedirect.com/science/article/pii/S1053811921003670.

Fan Li and Nancy R. Zhang. Bayesian variable selection in structured high-dimensional covariate spaces with applications in genomics. *Journal of the American Statistical Association*, 105(491):1202–1214, 2010a. ISSN 01621459. doi: 10.1198/jasa.2010.tm08177.

Fan Li and Nancy R. Zhang. Bayesian variable selection in structured high-dimensional covariate spaces with applications in genomics. *Journal of the American Statistical Association*, 105(491):1202–1214, 2010b.

Xinyi Li, Li Wang, Huixia Judy Wang, and for the Alzheimer's Disease Neuroimaging Initiative. Sparse learning and structure identification for ultrahigh-dimensional image-on-scalar regression. *Journal of the American Statistical Association*, 116(536):1994–2008, 2021.

Simon Lyddon, Stephen Walker, and Chris C. Holmes. Nonparametric learning from bayesian models with randomized objective functions. In *Advances in Neural Information Processing Systems 31: Annual Conference on Neural Information Processing Systems 2018, NeurIPS 2018*, pages 2075–2085, 2018.

K. Miller, F. Alfaro-Almagro, N. Bangerter, and David L. Thomas. Multimodal population brain imaging in the uk biobank prospective epidemiological study. *Nature Neuroscience*, (19):1523–1536, 2016.

Thomas E Minka. Expectation propagation for approximate bayesian inference. 2001. doi: 10.5555/2074022.2074067.

T. J. Mitchell and J. J. Beauchamp. Bayesian variable selection in linear regression. *Journal of the American Statistical Association*, 83(404):1023–1032, 1988.

Michael A. Newton and Adrian E. Raftery. Approximate bayesian inference with the weighted likelihood bootstrap. *Journal of the Royal Statistical Society: Series B (Methodological)*, 56(1):3–26, 1994.

Lizhen Nie and Veronika Ročková. Bayesian bootstrap spike-and-slab lasso. *Journal of the American Statistical Association*, 0(0):1–16, 2022.

Juho Piironen and Aki Vehtari. Sparsity information and regularization in the horseshoe and other shrinkage priors. *Electronic Journal of Statistics*, 11(2):5018 – 5051, 2017.

J. B. Poline and B. M. Mazoyer. Analysis of individual positron emission tomography activation maps by detection of high signal-to-noise-ratio pixel clusters. *Journal of Cerebral Blood Flow and Metabolism*, 13:425–437, 6 1993.

Veronika Ročková and Edward I. George. EMVS: The EM approach to Bayesian variable selection. *Journal of the American Statistical Association*, 109(506):828–846, 2014.

Veronika Ročková and Edward I. George. The Spike-and-Slab LASSO. *Journal of the American Statistical Association*, 113(521):431–444, 2018.

Arkaprava Roy, Brian J. Reich, Joseph Guinness, Russell T. Shinohara, and Ana-Maria Staicu. Spatial shrinkage via the product independent gaussian process prior. *Journal of Computational and Graphical Statistics*, 30(4):1068–1080, 2021.

Donald B. Rubin. The Bayesian Bootstrap. *The Annals of Statistics*, 9(1):130 – 134, 1981.

Francesco C. Stingo, Yian A. Chen, Marina Vannucci, Marianne Barrier, and Philip E. Mirkes. A Bayesian graphical modeling approach to microRNA regulatory network inference. *The Annals of Applied Statistics*, 4(4):2024 – 2048, 2010.

Xiao Wang, Hongtu Zhu, and Alzheimer's Disease Neuroimaging Initiative. Generalized scalar-on-image regression models via total variation. *Journal of the American Statistical Association*, 112(519):1156–1168, 2017.

Andrew Whiteman. Bayesian analysis of neuroimage data using gaussian process priors. 2022. URL https://deepblue.lib.umich.edu/handle/2027.42/174640.

Wenqiong Xue, F DuBois Bowman, and Jian Kang. A bayesian spatial model to predict disease status using imaging data from various modalities. *Frontiers in neuroscience*, 12: 184, 2018.

Yuling Yao, Aki Vehtari, Daniel Simpson, and Andrew Gelman. Yes, but did it work?: Evaluating variational inference. *PMLR*, 80:5581–5590, 2018.

Zijian Zeng, Meng Li, and Marina Vannucci. Bayesian Image-on-Scalar Regression with a Spatial Global-Local Spike-and-Slab Prior. *Bayesian Analysis*, pages 1 – 26, 2022.
