# Supplementary material for: Bayesian Lesion Estimation with a Structured Spike-and-Slab Prior
Source: J Am Stat Assoc. 2023 Nov 16;119(545):66–80. doi: 10.1080/01621459.2023.2278201 (PMC11315456; doi:10.1080/01621459.2023.2278201)
Supplement: Supplemental Material [file UASA_A_2278201_SM3036.pdf]

This form documents the artifacts associated with the article (i.e., the data and code supporting the computational findings) and describes how to reproduce the findings.

Part 1: Data

- ☐ This paper does not involve analysis of external data (i.e., no data are used or the only data are generated by the authors via simulation in their code).
- ☒ I certify that the author(s) of the manuscript have legitimate access to and permission to use the data used in this manuscript.

Abstract

The data from the UK Biobank consists of binary lesion masks which indicate the presence or absence of a lesion across the brain. In addition, the data includes subject-specific epidemiological data, such as age, sex, and head size.

- Simulation study: 2D lesion masks generated via data generating process described in Supplementary Materials Sec. 5
- UK Biobank: 3D lesion masks
- X: NxP matrix: subject-specific covariates
- Y: NxM matrix: lesion masks

Availability

- ☒ Data **are** publicly available.
- ☐ Data **cannot be made** publicly available.

If the data are publicly available, see the *Publicly available data* section. Otherwise, see the *Non-publicly available data* section, below.

Publicly available data

- ☐ Data are available online at:
- ☐ Data are available as part of the paper's supplementary material.
- ☒ Data are publicly available by request, following the process described here: https://www.ukbiobank.ac.uk/enable-your-research/apply-for-access
- ☐ Data are or will be made available through some other mechanism, described here:

Non-publicly available data

Description

File format(s)

- ☒ CSV or other plain text.
- ☒ Software-specific binary format (.Rda, Python pickle, etc.): pkcle
- ☐ Standardized binary format (e.g., netCDF, HDF5, etc.):
- ☒ Other (please specify): NIFTI file format (extension: .nii.gz) -> file format used to store MRI scans

Data dictionary

☐ Provided by authors in the following file(s):

☐ Data file(s) is(are) self-describing (e.g., netCDF files)

☒ Available at the following URL:

- Example synthetic data (2D-lesion masks for sample size N=1,000 and base rate intensity lambda=3, .csv files): https://anonymous.4open.science/r/BLESS-D30D/simulation_study/data/N1000lambda3P2
- Example syntehtic data (true parameter estimates & lesion rates for various base rate intensities, .RData files): https://anonymous.4open.science/r/BLESS-D30D/simulation_study/truth
- UK Biobank application (additional files needed for real data analysis, such as example mask for extracting relevant white matter locations across 3D MRI scan, .nii.gz file, or list of neighbors for every masked spatial location, .RData file): https://anonymous.4open.science/r/BLESS-D30D/UK_Biobank/mask

Additional Information (optional)

Part 2: Code

Abstract

Code to run the mass-univariate method Firth regression, which fits a model at each voxel independently, the Bayesian spatial model BSGLMM, and our method BLESS-VI & BB- BLESS. Additionally, we provide all code to generate figures & acquire cluster size distributions via FSL in a shell script.

Description

Code format(s)

☒ Script files
  - ☒ R
  - ☐ Python
  - ☐ Matlab
  - ☐ Other:

☒ Package
  - ☒ R
  - ☐ Python
  - ☐ MATLAB toolbox
  - ☐ Other:

☐ Reproducible report
  - ☐ R Markdown
  - ☐ Jupyter notebook
  - ☐ Other:

☒ Shell script

☒ Other (please specify): FSL (software tool to analyze MRI brain imaging data: https://fsl.fmrib.ox.ac.uk/fsl/fslwiki/FSLeyes)

Supporting software requirements

Version of primary software used R version R version 4.1.1

Libraries and dependencies used by the code

- deldir (1.0-6)
- spatstat (1.4-6)
- MASS (7.3-54)
- hutils (1.7.1)
- scales (1.1.1)
- dplyr (1.0.7)
- reshape2 (1.4.4)
- brglm2 (0.8.0)
- LaplacesDemon (16.1.6)
- logOfGamma (0.0.1)
- latex2exp (0.5.0)
- oro.nifti (0.11.0)

Supporting system/hardware requirements (optional)

We utilize high performance cluster computing for our analyses in order to run our simulation studies in parallel and access data from the UK Biobank.

Parallelization used

☐ No parallel code used
☐ Multi-core parallelization on a single machine/node
  – Number of cores used:
☒ Multi-machine/multi-node parallelization
  – Number of nodes and cores used: maximum at a time 3000 nodes with 1 core

License

☒ MIT License (default)
☐ BSD
☐ GPL v3.0
☐ Creative Commons
☐ Other: (please specify)

Additional information (optional)

Code is available at https://anonymous.4open.science/r/BLESS-D30D/.

Part 3: Reproducibility workflow

Scope

The provided workflow reproduces:

☒ Any numbers provided in text in the paper
☒ The computational method(s) presented in the paper (i.e., code is provided that implements the method(s))
☒ All tables and figures in the paper
☐ Selected tables and figures in the paper, as explained and justified below:

Workflow

Location

The workflow is available:

☐ As part of the paper's supplementary material.
☒ In this Git repository: https://anonymous.4open.science/r/BLESS-D30D/
☐ Other (please specify):

Format(s)

☐ Single master code file
☐ Wrapper (shell) script(s)
☐ Self-contained R Markdown file, Jupyter notebook, or other literate programming approach
☒ Text file (e.g., a readme-style file) that documents workflow
☐ Makefile
☐ Other (more detail in *Instructions* below)

Instructions

Expected run-time

Approximate time needed to reproduce the analyses on a standard desktop machine:

☐ < 1 minute
☐ 1-10 minutes
☐ 10-60 minutes
☐ 1-8 hours
☐ > 8 hours
☒ Not feasible to run on a desktop machine, as described here:

Simulation Study The analysis of a single dataset requires an average run time of 1-4min for Firth regression, 7-15h for BSGLMM for sampling 15,000 iterations and discarding 5,000 as burn-in, 3-6h for BLESS-VI for an DPE evaluation across a range of 15 spike variance values, 1-3min for a single optimization of BB-BLESS which can be run in parallel (if optimizations are run sequentially, then run time averages from 15-50h for B=1,000 bootstrap samples), and 10-20h for BLESS-Gibbs for sampling 15,000 iterations and discarding 5,000 as burn-in (minimum run time: sample size of N=500, maximum run time: sample size N=5,000). **UK Biobank application** The analysis of lesion incidence on the covariates, age, sex, age by sex, and head size scaling factor, for a sample size of N=2,000 has an approx. computational cost of 1h for Firth regression if no parallelization is used and each voxel is evaluated sequentially, 21h for BLESS-VI for an DPE evaluation across a range of 5 spike variance values, and 6h for a single optimization of BB-BLESS which can be run in parallel for B=1,500 bootstrap samples.

Additional information (optional)

Notes (optional)
